# Towards trustworthy AI-driven cuffless blood pressure monitoring

**DOI:** 10.1038/s41746-026-02898-7

**Published:** 2026-06-25

**Authors:** Ana Cisnal, Itilekha Podder, Leoni Grossmann, Kanika Dheman, Mohamed Elgendi, Marco Valgimigli, Diego Paez-Granados

**Affiliations:** 1 Spinal Cord and Artificial Intelligence (SCAI) Lab, D-HEST, ETH Zürich, Zürich, Switzerland; 2 Institute of Advanced Production Technologies, Valladolid University, Valladolid, Spain; 3 Digital Health Care and Rehabilitation, Swiss Paraplegic Research (SPF), Notwill, Switzerland; 4 Faculty of Informatics, Eötvös Loránd University, Budapest, Hungary; 5 Centre for Psychiatry Research, Department of Clinical Neuroscience, Karolinska Institutet, and Stockholm Health Care Services, Stockholm, Sweden; 6 Center for Project Based Learning Lab, ETH Zurich, Zurich, Switzerland; 7 Department of Biomedical Engineering and Biotechnology, Khalifa University of Science and Technology, Abu Dhabi, UAE; 8 Healthcare Engineering Innovation Group (HEIG), Khalifa University of Science and Technology, Abu Dhabi, UAE; 9 Department of Biomedical Sciences, University of Italian Switzerland, Cardiocentro Ticino Institute, Ente Ospedaliero Cantonale, Lugano, Switzerland

**Keywords:** Cardiology, Computational biology and bioinformatics, Engineering, Health care, Mathematics and computing, Medical research

## Abstract

Cuffless blood pressure monitoring is becoming increasingly feasible with the rise of wearable technologies, offering significant promise for preventive cardiovascular care. Yet diverse sensing modalities and modeling strategies have produced fragmented evidence that is difficult to compare. In this systematic review, we introduce a unified multi-axis taxonomy that relates surrogate signals, modeling paradigms, and calibration strategies, providing a coherent structure for the field. Building on this foundation, we analysed current approaches across physiological and wearable sensing and evaluated performance using best machine-learning practices. We highlight key barriers to real-world deployment—including protocol realism, calibration drift, population diversity, and fairness relevant reporting—and translated these findings into an actionable model-card-style reporting framework for cuffless BP studies. Our framework establishes the methodological basis for reliable, equitable, and clinically valid cuffless BP monitoring, a vital step towards democratizing continuous BP monitoring for widespread clinical benefit.

## Introduction

**Fig. 1:**
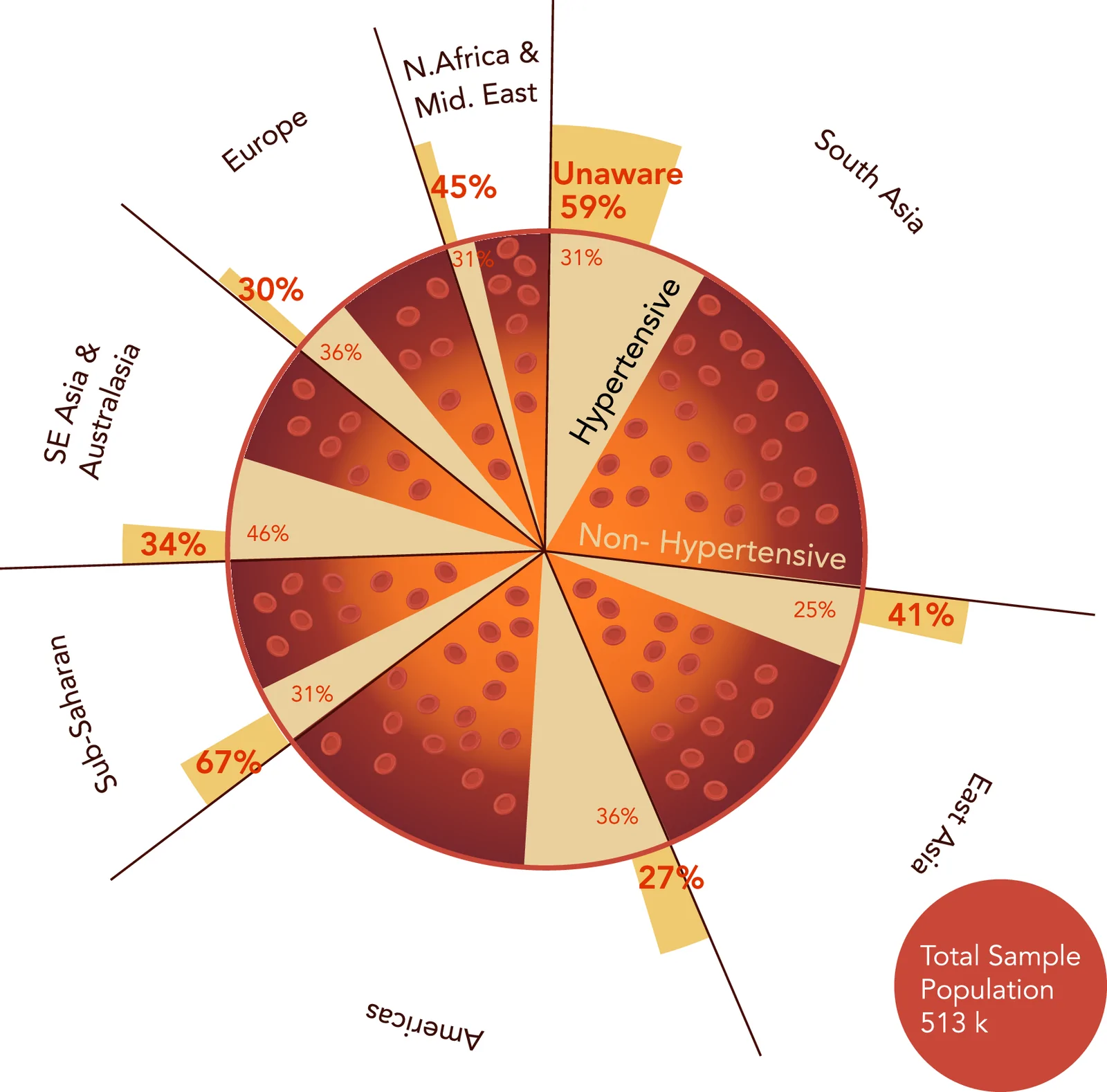
Hypertension prevalence and awareness. 44.3% of hypertensive adults are unaware of their undiagnosed condition.

Accurate blood pressure (BP) measurements are vital in the prevention and treatment of cardiovascular diseases (CVDs), which are the leading cause of death globally^1^. High BP is a modifiable risk factor for CVDs, and its effective management can delay or even prevent the onset of CVD-related morbidity and mortality. The initial step in managing hypertension (HTN) involves detecting pathologically elevated BP levels. Despite the widespread prevalence of HTN, most affected individuals remain unaware of their condition. Out of 1.5M adults screened in ref. ^2^, 32.0% had never had a BP measurement before and 34.0% had HTN, with a total of 41% unaware of their condition (see Fig. 1).

The traditional cuff-based sphygmomanometer remains the state-of-the-art device for BP measurement, primarily used in clinical settings. However, the white coat—a phenomenon where BP changes during a clinical visit^3^. Self-measured home BP and ambulatory BP monitoring are out-of-office BP, and they have been shown to exert more prognostic ability than conventional in-office BP^4–6^. Additionally, BP fluctuates throughout the day, influenced by numerous internal and external factors. BP rises sharply upon waking and falls during sleep, with variations occurring throughout the day. Consequently, major guidelines recommend the use of ambulatory or home BP measurement in the diagnosis of HTN^7,8^. Beyond its greater predictive power, home BP can substantially refine conventional in-office BP-based risk stratification^9,10^.

In this context, consumer-grade devices (smartwatches) aim at measuring BP using surrogate vital sensing, such as, photoplethysmography (PPG) and/or electrocardiography (ECG). Some devices even meet medical device grades as outlined in the International Organization for Standardization (ISO) 81060-2:2018 for noninvasive sphygmomanometers and have been cleared by the United States Food and Drug Administration and the European Union (CE-label for Conformity Marketing).

However, the algorithms and validation studies for these devices are not openly disclosed, and most of them only utilize data collected from healthy individuals^11^. Due to the currently limited validation of the technology and the low reliability of smartwatch-based BP measurements^12^, their usage is not yet recommended for monitoring treatment response in hypertensive patients or those with cardiovascular comorbidities^13^. Moreover, current validation standards for cuff BP devices are not suitable for data-driven approaches^14,15^. Until specific standards, education, and clinical translation are proven for data-based cuffless BP devices, and their accuracy and clinical utility thoroughly examined, these devices should not be recommended for medical decision-making, diagnosis, or treatment^16,17^.

Despite more than 86 published methods evaluated in this work, no generalizable, clinically validated cuffless BP system currently exists. Existing reviews have provided valuable summaries of sensor technologies, model architectures, and signal-processing pipelines in cuffless BP estimation and hypertension monitoring. However, most prior reviews remain primarily descriptive or engineering-oriented: they summarize what sensors and algorithms have been used, but provide limited empirical evidence on whether reported models are evaluated in ways that support generalization, regulatory alignment, or clinical translation^18–20^. In particular, key machine learning (ML)-specific questions remain insufficiently addressed: whether validation is subject-independent, whether calibration-based and calibration-free approaches are evaluated under appropriate assumptions, whether reported errors are robust across study designs and realistic ambulatory conditions, whether subgroup-level reporting is sufficient for future fairness evaluation, and whether published results can be compared transparently across heterogeneous datasets and protocols.

To position our contribution relative to this literature, Table 1 summarizes existing reviews across key methodological, evaluative, and translational dimensions. Among recent works, Cheung et al.^21^ is the most closely related, combining systematic review with quantitative benchmark analysis and calibration-aware evaluation. Their work makes an important contribution by adopting a hardware-to-algorithm perspective, organizing the field by pipeline stage, and introducing explained deviation as a study-level normalization metric. However, it does not provide formal random-effects meta-analytic pooling, publication-bias diagnostics, study-level risk-of-bias assessment, or a reusable reporting instrument tailored to ML-based cuffless BP evaluation. Other recent reviews and statements provide important methodological or clinical guidance, but do not jointly integrate an ML-oriented taxonomy, quantitative synthesis of reported error, validation-design subgroup analysis, demographic and subgroup reporting-gap assessment, and a model-card-style reporting framework.

**Table 1:** Gap analysis of prior reviews and guidance relevant to cuffless blood pressure monitoring

Prior work	Review type	Taxonomy	Meta analysis	Validation audit	Calibration-aware eval.	Fairness reporting	Clinical translation	Structured framework
El-Hajj et al.^18^	Narrative review	○	○	○	◐	○	◐	○
Martínez-Ríos et al.^20^	Narrative review	○	○	○	◐	○	◐	○
Mukkamala et al.^149^	Methodological perspective	○	○	●	●	○	●	◐
Qin et al.^182^	Methodological review	●	○	◐	◐	○	◐	○
Sastimoglu et al.^183^	Systematic review + meta-analysis	◐	●	◐	◐	◐	●	○
Cheung et al.^21^	Systematic review + benchmark analysis	●	◐	●	●	◐	◐	◐
Min et al.^58^	Narrative review	●	○	◐	◐	◐	●	○
Cohen et al.^184^	Scientific statement	○	○	●	●	◐	●	◐
**This review**	**Systematic review** **+** **meta-analysis**	●	●	●	●	◐	◐	●

Symbols indicate the depth of coverage: ● = substantively addressed; ◐ = partially addressed; ○ = not addressed. Ratings reflect scope of coverage rather than completeness.

Taxonomy = explicit methodological or machine-learning taxonomy; Validation audit = explicit audit of validation design and generalizability/leakage risk; Calibration-aware eval. = explicit distinction between calibration-based and calibration-free evaluation logic; Fairness reporting = assessment of demographic, clinical, and subgroup-level reporting, including whether studies report subgroup-stratified performance required for future fairness evaluation; Clinical translation = explicit discussion of real-world clinical applicability, deployment constraints, or guideline relevance; Structured framework = reusable reporting framework, checklist, reporting card, or model-card-like guidance.

Therefore, the added value of the present review is that it links methodological classification, empirical evidence synthesis, and reporting guidance into a single evaluation-oriented framework. Specifically, we make four contributions. First, we introduce a three-axis orthogonal taxonomy that separates the signal-to-target relationship, modeling paradigm, and calibration strategy, thereby distinguishing fundamentally different algorithmic claims that are often conflated in prior work. Second, we perform a random-effects meta-analysis of reported SBP mean error with validation-strategy subgroup analyses, sensitivity analyses, and publication-bias diagnostics, allowing heterogeneity and potential optimistic reporting to be examined empirically rather than discussed only qualitatively. Third, we assess demographic, clinical, and subgroup-level reporting practices relevant to future fairness evaluation, clarifying that the current literature supports a reporting-gap analysis. Fourth, we translate these findings into the BP-Card, a model-card-inspired reporting framework that separates calibration-free and calibration-based evaluation pathways and operationalizes minimum reporting requirements for cohort description, validation design, explicit baseline-model comparison to contextualize predictive performance, standardized metrics, and subgroup-relevant reporting.

In this work, we provide a systematic review and meta-analysis of cuffless BP estimation methods beyond PPG, evaluating the quality, robustness, and generalizability of reported results in ML-based approaches in relation to clinical guidelines and emerging validation expectations. By combining an ML-oriented taxonomy, quantitative evidence synthesis, validation-design analysis, fairness-relevant reporting-gap assessment, and the BP-Card framework, this review provides a structured basis for interpreting the current evidence and identifying the methodological and reporting factors that limit comparability across studies. The resulting synthesis clarifies how validation strategy, calibration paradigm, cohort reporting, and performance reporting shape the interpretation of reported accuracy, and proposes practical reporting guidance for future cuffless BP studies.

## Results

**Fig. 2:**
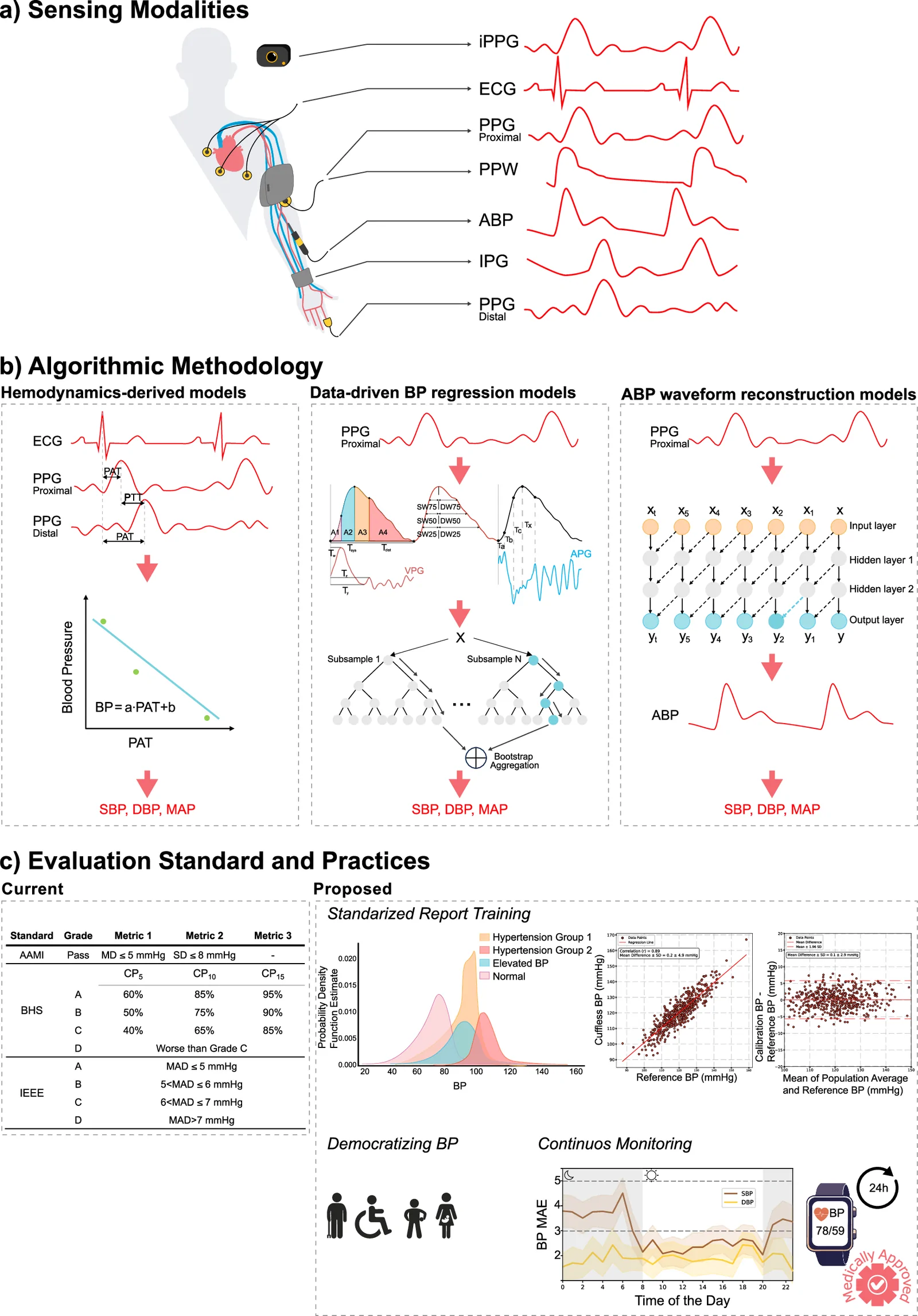
Conceptual overview of cuffless BP estimation. Signals, algorithms, and evaluation. **a** Overview of commonly used physiological waveforms for cuffless BP estimation and their temporal alignment, including Imaging Photoplethysmography (iPPG), Electrocardiography (ECG), proximal and distal Photoplethysmography (PPG), pulse pressure wave (PPW), and Arterial Blood Pressure (ABP), Impedance plethysmography (IPG). Pulse Arrival Time (PAT) and Pulse Transit Time (PTT) are also indicated. **b** Categories of BP estimation algorithms based on the proposed taxonomy: hemodynamics-derived models, data-driven BP regression models, and ABP waveform reconstruction models. **c** We propose to standardize these data and the population behind the different modeling types with the goal of achieving equitable algorithms for continuous BP monitoring.

In this section, we present the main findings of our systematic review, structured to reflect the three key components of cuffless BP estimation: sensing modalities, algorithmic methodology and evaluation standards and practices (Fig. 2). We begin by summarizing the sensing modalities and databases used in the literature, highlighting trends in physiological signals, data sources and population characteristics. We then analyses algorithmic methodologies using our proposed multi-axes taxonomy. Additionally, we review validation practices and performance reporting across studies, followed by a quantitative meta-analysis assessing reproducibility, robustness, and methodological consistency.

Table 2 provides an overview of all 86 studies included in the review, summarizing data source, sensing modality, device location, sample size, reference BP device, validation strategy, and whether the proposed approach is calibration-free. These characteristics are further analyzed in the following subsections.

**Table 2:** Summary of the 86 studies included in the systematic review, grouped by algorithm category

Ref	Year	Data source^a^	Sensor	Device location^b^	Participants	Reference^c^	Evaluation method	CF^d^
**Hemodynamics-derived models**								
^79^	2017	Own data	ECG, PPG	ECG L&R arm; PPG finger	33^e^	Cuff	Subject-specific calibration^f^	No
^72^	2017	Own data	ECG, 2 PPGs	PPG finger; ECG L&R hand	32	Cuff (mercury BPMD)	Leave-one-pair-out^f^	No
^77^	2017	Own data	ECG, PPG	Ear	14	Cuff (BP monitor)	Hold-out 30:30 min^f^	No
^78^	2018	MIMIC I	ECG, PPG	-	23	ABP (invasive)	Leave-one-pair-out^f^	No
^59^	2020	Own data	ECG, PPG	PPG finger; ECG both hands	12	Cuff (BP monitor)	CV^f^	No
^69^	2021	Own data	IPG, PPG	Wrist	30	Cuff (BP monitor)	Two-step calibration	No
^81^	2021	Own data	6 PPGs	Wrist	8	Cuff (volume finger clamp)	-^f^	No
^80^	2022	Own data	2 PPGs	Finger	26^e^	Cuff	Hold-out 75:25, 5-fold CV	No
^55^	2022	SHIMMER/MIMIC-III	ECG, PPG	-	6/NA	Cuff/ABP (invasive)	Hold-out 75:25	No
^83^	2022	Own data	PTT	NA	32^e^	Cuff (aneroid BPMD)	-	Yes
^50^	2023	Own data/MIMIC-III	PPG	Finger	150	Cuff (BPMD), ABP (invasive)	Subject-specific calibration^f^	No
^32^	2023	Own data	PPG, PZT	Same wrist	129^e^	Cuff (BPMD)	Individual estimations	Yes
^85^	2023	Own data	PPG, BCG	NA	22	Cuff (volume clamping)	Subject-specific calibration^f^	No
^26^	2023	CTBP	iPPG	Face	93^e^	Cuff (electronic BPMD)	10-fold CV^f^	Yes
**Data-driven BP regression models**								
^91^	2017	Own data	ECG, PPG	ECG L&R arm, R leg; PPG finger	73^e^	Cuff (BP monitor)	Hold-out	No
^116^	2018	Own data	ECG, PZT	Hand	65	Cuff (BP monitor)	Hold-out^f^	No
^115^	2018	Own data	ECG	Chest	51	Cuff (electronic BPMD)	Hold-out 60:10:30	No
^33^	2018	Own data	ECG, PPG, SPO2	Ear	18	Cuff (BP monitor)	5-fold CV	No
^125^	2018	Own data	ECG, PPG	PPG finger; ECG R&L leg, R arm	12^e^	Cuff (volume clamping)	Hold-out 80:20, 10-fold CV	Yes
^121^	2019	UCI	ECG, PPG	-	942	ABP (invasive)	Hold-out 70:10:20	No
^128^	2020	MIMIC-II	ECG	-	NA	ABP (invasive)	Hold-out 80:10:10	Yes
^52^	2020	MIMIC-II & UQVS	PPG	-	283	ABP (invasive)	Hold-out 50:50	No
^147^	2020	Own data	ECG, PPG	ECG L/R arm, leg; PPG finger	85	Cuff (BP monitor)	Subject-specific calibration^f^	No
^129^	2020	MIMIC-II	PPG	-	1557	ABP (invasive)	10-fold CV	Yes
^127^	2020	Own data	ECG, PPG	Wrist	110^e^	Smart wristwatch	Hold-out 60:40^f^	No
^53^	2021	PPG-BP/MIMIC-II	PPG	-	NA	ABP (invasive)	Hold-out	Yes
^109^	2021	Own data	ECG, PPG	Chest and wrist	26^e^	Cuff (pulse rate monitor)	10-fold CV	No
^113^	2021	VitalDB	ECG, PPG	Chest and wrist	NA^e^	ABP (invasive)	10-fold CV	No
^130^	2021	MIMIC-I	ECG, PPG	-	45	ABP (invasive)	10-fold CV	Yes
^131^	2021	MIMIC-II	ECG	-	NA	ABP (invasive)	Hold-out 80:10:10	Yes
^99^	2021	UCI	ECG, PPG	-	8000^e^	ABP (invasive)	Hold-out 70:30	Yes
^102^	2021	UCI	PPG	-	NA^e^	ABP (invasive)	Hold-out 60:20:20	Yes
^86^	2022	UCI	ECG, PPG	-	3295	ABP (invasive)	CV	Yes
^118^	2022	Y. Liang et al.	PPG	Finger	219^e^	Cuff (mercury BPMD)	Hold-out 80:20	Yes
^101^	2022	UCI	PPG	-	348^e^	ABP (invasive)	Hold-out 85:15, 5-fold CV	No
^119^	2022	Own data	PPG	Wrist	33^e^	Cuff (electronic BPMD)	Hold-out 70:15:15	yes
^126^	2022	MIMIC-II	PPG	-	9000^e^	ABP (invasive)	Hold-out 70:10:20	Yes
^132^	2022	MMIC-II	PPG	-	912	ABP (invasive)	10-fold CV	Yes
^87^	2022	MIMIC-I	ECG, PPG	-	100	ABP (invasive)	Hold-out	Yes
^133^	2022	Own data	iPPG	Face	129^e^	Cuff (electronic BPMD)	Hold-out 60:20:20	Yes
^134^	2022	UCI	PPG	-	942^e^	ABP (invasive)	Hold-out 80:10:10, 10-fold CV	No
^124^	2022	MIMIC-III	PPG	-	100^e^	ABP (invasive)	5-fold CV^f^	No
^135^	2022	MIMIC-III	PPG	-	150^e^	ABP (invasive)	Hold-out 50:50, 10-fold CV	Yes
^27^	2022	Own data	iPPG	Face	814^e^	Cuff (electronic BPMD)	Hold-out 80:20, 5-fold CV	Yes
^88^	2022	MIMIC-II	ECG, PPG	-	1551	ABP (invasive)	Hold-out, 10-fold CV	Yes
^122^	2022	MIMIC-II	ABP, ECG	-	NA^e^	ABP (invasive)	Hold-out 80:10:10	No
^89^	2022	MIMIC-III	PPG	-	255^e^	ABP (invasive)	Hold-out 80:20^f^	No
^110^	2023	Own data	PPG	Finger	40^e^	ABP (invasive)	Hold-out 80:20^f^	No
^136^	2023	Own data	OMW	Wrist, upper arm	150^e^	Cuff (Omron monitor)	LOOCV^f^	No
^114^	2023	MIMIC-III	PPG	-	510	ABP (invasive)	-	Yes
^107^	2023	Own data	iPPG	Face	220^e^	Cuff (electronic BPMD)	Hold-out, 5-fold CV^f^	Yes
^137^	2023	VitalDB	PPG	-	4185^e^	Cuff	Hold-out 70:10:20^f^	No
^138^	2023	Own data	PPG	Finger	476^e^	Cuff (BPMDs)	Hold-out 5-fold CV^f^	Yes
^49^	2023	Own data	ECG, MWPPG	Wrist	3077^e^	Cuff (mercury BPMD)	5-fold CV^f^	Yes
^105^	2023	Own data	ECG, PPG	-	62^e^	ABP (noninvasive)	LOOCV^f^	No
^123^	2023	MIMIC-III	PPG	-	NA	ABP (invasive)	5-fold CV	Yes
^117^	2023	Own data	IPG	-	8^e^	Cuff	5-fold CV^f^	No
^51^	2023	Own data	ECG, PPG	Finger, chest	23	Cuff (mercury BPMD)	-	No
^120^	2023	Own data	Capacitance PG	Wrist or foot	306	Cuff	10-fold CV	Yes
^100^	2023	MMIC-II	PPG	-	4000	ABP (invasive)	Nested 10-fold CV	Yes
^148^	2023	Own data	PPG	Arm	30^e^	Cuff (BPMD)	Hold-out	Yes
^108^	2023	Own data	iPPG	Face	1138^e^	Cuff (mercury BPMD)	Hold-out	No
^84^	2023	Own data	PPG	Finger	40^e^	Cuff	LOOCV^f^	No
^185^	2024	Own data	iPPG	Face	24^e^	ABP (invasive)	LOOCV^f^	Yes
^95^	2024	VitalDB	PPG, ECG	-	1675^e^	Cuff	Hold-out 4:1	Yes
^179^	2024	PPG-BP	PPG	Finger	219^e^	ABP (invasive)	Hold-out 60:15:25	Yes
^34^	2024	CAS-BP	PPG, ECG, PPW	Wrist	1272^e^	Cuff	Hold-out 60:20:20	No
^186^	2024	MIMIC-II	PPG, ABP	-	1690^e^	ABP (invasive)	10-fold CV	Yes
^94^	2024	Own data & MIMIC II	PPG	Finger	683^e^	ABP (invasive)	Hold-out 80:10:10^f^	Yes
^93^	2024	AuroraBP	PPG	Wrist	523^e^	Cuff	LOOCV^f^	Yes
^90^	2024	UCI	ECG, PPG	Finger, chest	NA	ABP (invasive)	Hold-out 4:1	No
^92^	2024	PulseDB	PPG	Finger	5^e^	ABP (invasive)	Hold-out 80:10:10	Yes
^35^	2024	Own data	PPG, iPPG, PPW, Demographic	Wrist	2073^e^	Cuff (electronic BPMD)	Hold-out 9:1	Yes
^106^	2025	HRSDs (Own data)	PPG (single-channel)	Upper arm	139^e^	Cuff (mercury BPMD)	Hold-out 8.5:1.5	Yes
^103^	2025	PulseDB	ECG, PPG	-	1231^e^	ABP (invasive)	Hold-out	No
^104^	2025	PulseDB	ECG, PPG	-	1293^e^	ABP (invasive)	Hold-out	No
^139^	2025	PulseDB	ECG, PPG, ABP	-	5533^e^	ABP (invasive)	Hold-out 80:20:20, 5-fold CV	No
**ABP waveform reconstruction models**								
^140^	2020	Own data	ABP, PPG	PPG finger, ABP cuff	18	ABP (noninvasive)	Hold-out 85:15, CV	No
^54^	2021	UCI/UQVS	PPG	-	NA	ABP (invasive)	LOOCV^f^	No
^154^	2021	MIMIC-II	ABP, PPG	-	5289	ABP (invasive)	Hold-out 70:10:20	Yes
^141^	2021	MIMIC-II	PPG	-	3183^e^	ABP (invasive)	Hold-out 75:15:15	Yes
^144^	2022	Own Data/MIMIC	PPG	-	467/NA	ABP (invasive)	Hold-out 70:15:15^f^	No
^143^	2023	Own data	rBP	Face	1032^e^	ABP (noninvasive)	Hold-out 2:1	Yes
^146^	2024	MIMIC-II	PPG, ABP	-	8746^e^	ABP (invasive)	Hold-out 70:10:20	Yes
^142^	2024	MIMIC-III	PPG, ABP	-	15^e^	ABP (invasive)	Hold-out	No
^145^	2025	MIMIC-III	PPG	-	732^e^	ABP (invasive)	Hold out 80:20, 5-fold CV	Yes

For each study, we report publication year, data source, sensing modality, device location, number of participants, reference BP measurement, evaluation method, and whether the approach is calibration-free (CF).

*L* left, *R* right, *CV* Cross-validtaion, *LOOCV* leave-one-out CV.

^a^ When multiple databases are utilized, “/” denotes independent usage, while “&” denotes combined usage.

^b^When utilizing a public database, “-” indicates its usage.

^c^BPMD: Blood pressure monitoring device. ABP: arterial blood pressure.

^d^CF refers to whether the proposed method is calibration-free.

^e^Indicates that data on the distribution of blood pressure within the cohort are provided.

^f^Indicates that the proposed method includes subject-wise validation.

### Sensing modalities and data sources

Accurate BP estimation with non-invasive ambulatory sensors depends on the careful pre-processing of physiological signals, which typically involves baseline drift correction, proper signal alignment, and artifact removal. Signal quality can be significantly affected by both biological factors (e.g., tissue composition, respiration, vasomotor activity, or autonomic dysregulation) and external influences (e.g., light exposure or motion)^22^. To address this, signal processing techniques such as filtering, normalization, and segmentation are commonly applied. Although many advanced ML algorithms are capable of extracting meaningful features directly from raw physiological signals, in the reviewed literature, only 31% (*n* = 27 out of 86) of studies utilize raw signals, while the remaining works rely on features derived from preprocessed data.

Current common cuffless methods for measuring hemodynamic activity through waveform recording are imaging photoplethysmography (iPPG), ECG, PPG, impedance plethysmography (IPG) and pulse pressure waveform (PPW) (Fig. 2a). Unlike traditional arterial blood pressure (ABP) measurement, these methods allow for continuous BP monitoring. Although these methods show promising results, it is essential to understand and validate their estimations against traditional cuff-based BP monitors, as they offer indirect and proxy-data of the actual BP^12,23^.

PPG is a non-invasive optical technique used to quantify the arterial volume pulse by measuring dynamic variations in light absorption or reflection as blood circulates through peripheral vessels. A PPG sensor emits light into the underlying tissue, and the blood in nearby vessels absorbs part of this light while the rest is scattered back to the sensor^24^. PPG can be performed in reflectance or transmission modes^22^. Transmission-mode is limited to body sites where the optical light source and photodetector can enclose a pulsating blood vessel, such as the earlobe, fingertip and toes, while reflectance-mode offers more placement flexibility but is more susceptible to motion artifacts^25^. PPG signal amplitude is influenced by sensor positioning and contact pressure. Factors like ambient light, motion, skin color, keratinization, underlying adiposity and follicular density also impact signal quality.

iPPG, also known as remote photoplethysmography (rPPG), is a non-contact optical imaging method^26^. iPPG is based on capturing 2-D images of the uncovered skin using a high-definition digital camera^27^, detecting fluctuations in reflected light caused by heart volume pulse. It enables unobtrusive monitoring and flexible positioning, though its accuracy is strongly affected by motion and lighting artifacts.

ECG is a non-invasive technique that monitors heart activity through electrodes to capture electrical impulses produced during cardiac cycles. It is widely used for heart rate and rhythm monitoring. However, like PPG, it is prone to motion artifacts and depends on proper electrode placement. Its role in BP estimation is less direct, often requiring combination with other signals.

IPG measures bio-impedance to track volumetric changes in blood flow, typically using a tetrapolar electrode setup^28^. While promising, it is highly susceptible to motion artifacts, pressure, or temperature changes, and has a low signal-to-noise ratio, especially in individuals with poor circulation or health conditions, limiting its broader applicability.

Tonometry measures arterial wall pressure by applying controlled external force with a contact sensor to partially flatten the artery, thereby recording the resulting deformation and acquiring the PPW. Tonometric devices may incorporate optical, mechanical, laser-based or PZTs. Although more invasive than PPG or IPG due to the need for direct skin contact, tonometry offers relatively higher accuracy for BP estimation^29,30^.

**Fig. 3:**
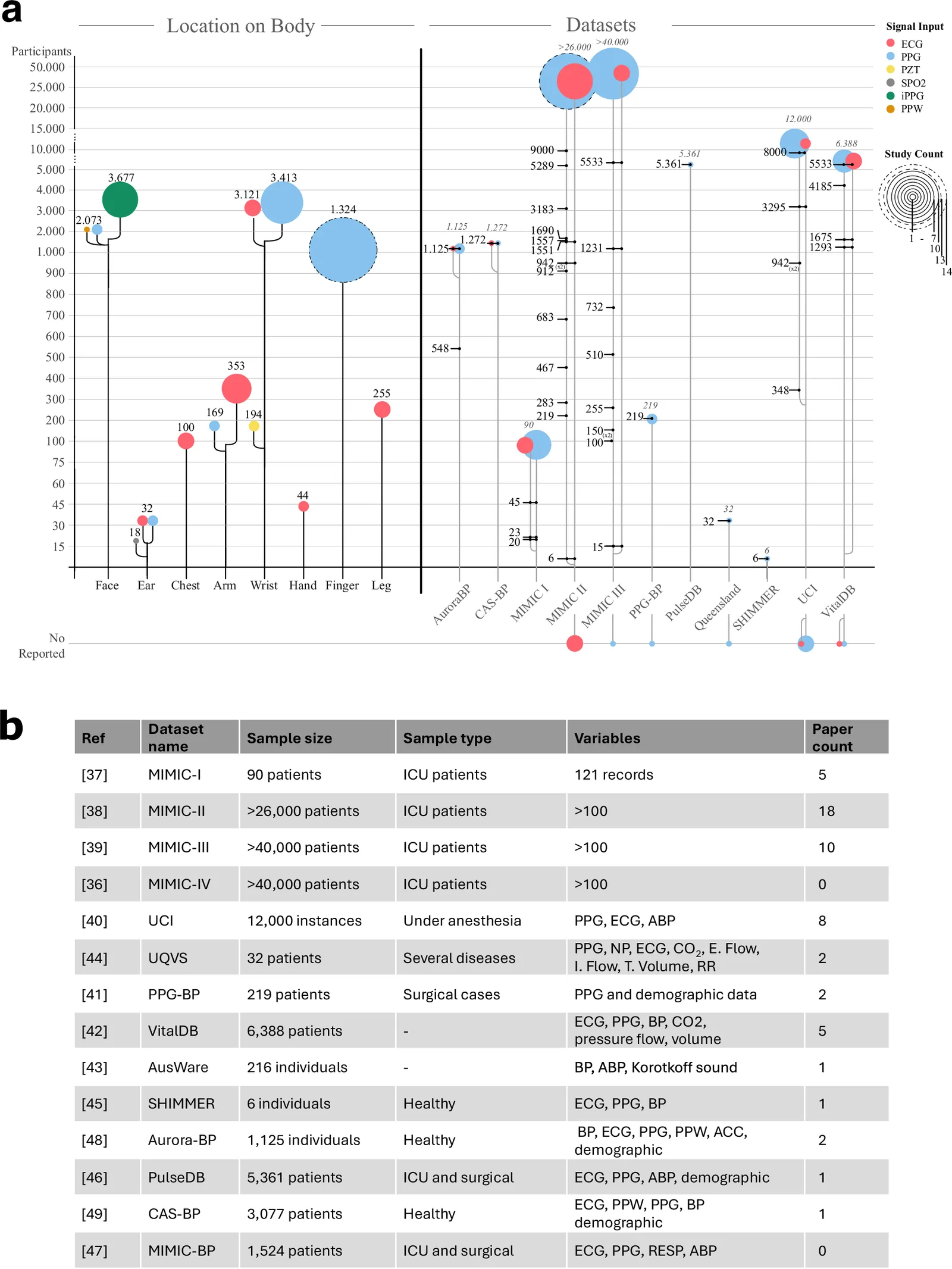
Overview of sensors and datasets used for BP estimation. **a** Sensor location, signals and datasets. Left part displays the locations of cuffless devices and the measured signals, including electrocardiogram (ECG), photoplethysmography (PPG), piezoelectric transducer (PZT), blood oxygen saturation (SPO2), imaging photoplethysmography (iPPG), and pulse pressure waveform (PPW). Signal type distribution is shown for each measurement location in the identified studies. The right part displays the number of articles that utilized public databases and the number of subjects included. Circle size represents the population size. **b** Summary of public datasets used in the literature.

Table 2 and Fig. 3a summarizes the signal modalities used in the reviewed studies, indicating their measurement location and sample size. We found the majority of studies (*n* = 70 out of 86) used PPG sensors, with 37 relying solely on PPG. PPG was most commonly combined with ECG, observed in 29 studies. In contrast, ECG was rarely used alone, appearing in only 4 studies. This pattern is consistent with the comparative analysis by Finnegan et al.^31^. They systematically evaluated ECG- and PPG-derived features for estimating changes in blood pressure (ΔBP) and found that PPG-based models consistently outperformed ECG-only models, while adding ECG features to PPG did not yield appreciable gains. Physiologically, the ECG–BP relationship is mediated by mechano-electric coupling, a cyclical interaction between cardiac electrical activity and mechanical contraction that is strongly influenced by autonomic, hormonal, and environmental factors and is therefore less specific to BP. In contrast, PPG directly captures peripheral blood volume changes and wave reflections, providing a more direct haemodynamic surrogate^31^.

A total of 35 publications employed two or more signals in combination, highlighting the dominance of either PPG-only approaches or multimodal sensing strategies. iPPG, one of the most recent methods, was used independently in 6 studies, while IPG was used alone in only one. No reviewed studies employed PPW or Peripheral Capillary Oxygen Saturation (SPO2) signals by themselves. The Piezoelectric Transducer (PZT) sensors were used in conjunction with ECG in a single study^32^, while SPO2 was included in only one study alongside ECG and PPG^33^. PPW was employed in two studies: one in combination with ECG and PPG^34^, and another with PPG and iPPG^35^. From all identified studies, the finger was the most common location for BP estimation using PPG sensors (*n* = 18), followed by the wrist (*n* = 11). In contrast, locations such as the face, ear, or chest were used less frequently, primarily in the context of recent sensor modalities of iPPG and ECG.

We also systematically reviewed all databases referenced across the included studies and summarized their main characteristics in Fig. 3b. To contextualize how these datasets are used in practice, Fig. 3a provides an overview of database usage and sample sizes across studies. Among these, the Medical Information Mart for Intensive Care (MIMIC) database series was the most commonly used, appearing in 31 studies in total, specifically, MIMIC-I in 3, MIMIC-II (Multi-parameter Intelligent Monitoring for Intensive Care) in 18, and MIMIC-III (Medical Information Mart for Intensive Care) in 10. MIMIC-IV^36^, which was released in March 2021, had not yet been adopted in any of the reviewed studies, likely due to its recent release at the time.

On the other hand, the UC Irvine (UCI) database, a meticulously preprocessed subset of MIMIC, found its place in 8 articles. VitalDB appeared in 5 articles, while University of Queensland Vital Signs (UQVS), PPG-BP, and AuroraBP were each used in 2 studies. CAS-BP, PulseDB, and AuscWave appeared less frequently, with each being used in only one study. Noteworthy finding was a proportion of published works 17% (13 out of 78), where authors fail to specify the number of subjects used in their studies, particularly when utilizing public databases.

The original MIMIC (1992–1999)^37^ included recorded data from over 90 emergency care unit patients. Afterwards, MIMIC-II^38^ and MIMIC-III^39^ were released, which are many times the size of the original database. Additionally, the “Cuffless Blood Pressure Estimation Data Set”^40^, which is freely available on UCI Machine Learning Repository, contains preprocessed and validated ECG, PPG and ABP signals from MIMIC. The PPG-BP database^41^ contains 657 segments of PPG signals, each lasting 2.1 seconds, from 219 subjects, including individuals with conditions such as HTN and diabetes. Three good-quality PPG signal segments at rest position were saved from each subject, along with one measurement of reference BP. VitalDB ^42^ contains time-synchronized bio-signals (ABP, ECG, and PPG) from 6,388 patients (13,489 h recorded time). AusWare^43^ includes 350 upper arm BP records from 150 individuals, collected using a multi-parameter clinical monitoring unit, with reference BP values derived from automated processing of Korotkoff sounds. UQVS dataset^44^ comprises monitoring data and vital signs collected from 32 surgical cases involving anesthesia. This SHIMMER database^45^ consists of 50 recordings, each around 20 min, from 6 healthy subject at rest. ECG signals were captured using three electrodes for left arm - right arm derivation, while PPG signals were recorded with clip on the left index finger. Additionally, BP readings were taken every minute using the OMRON HeartGuide smartwatch worn on the right wrist.

PulseDB^46^ introduced in 2023 emerged as the largest cleaned dataset with 5,245,454 high-quality 10-s segments of ECG, PPG, and ABP waveforms from 5361 subjects. The data sourced from a subset of the MIMIC-III and the VitalDB databases, also includes demographic information and characteristic points of ECG and PPG signals, making it an excellent resource for benchmarking BP estimation models. Despite its scale, only one study has utilized PulseDB so far. In 2024, another curated subset of MIMIC-III was released as MIMIC-BP^47^, offering 380 hours of recordings from 1524 subjects. Each subject has 30 segments of 30-s recordings, including PPG, ECG and RESP signals. Unlike UCI dataset, MIMIC-BP retains subjects’ identifiers to prevent data leakage between training and testing sets. Demographic data is not included, but can be accessed in the original MIMIC dataset. MIMIC-BP provides a balanced representation of BP values, making it a promising benchmark dataset for BP estimation models, but it has not yet been used in any of the reviewed studies.

Aurora-BP^48^ was released in 2022 as the first large-scale study to assess the performance of wearable tonometric, ECG, and PPG devices on both healthy and hypertensive adults in an ambulatory settings. This marks a significant advancement for the development of ABP estimation models compared to other public databases, which are in-clinic. In 2023, the CAS-BP dataset^49^ was introduced through a large-scale validation study. It includes over 29,000 high-quality BP recordings collected from 3077 adults using a smartwatch equipped with ECG, multi-wavelength PPG, and PPW sensors. Reference BP was measured using mercury sphygmomanometers and dual-observer auscultation. The dataset captures a broad range of BP values, including 36% hypertensive individuals.

A critical aspect of BP estimation models is the inclusion of diverse BP ranges. Combining databases offers access to a larger and more varied pool of data, covering a wider BP spectrum. For instance, while the MIMIC database primarily features normotensive and hypertensive measurements, the UQVS dataset includes hypotensive data, thereby enhancing its suitability for comprehensive BP estimation research. Several studies adopted a strategy of using multiple databases. For example^50^, combined their own data with MIMIC-III^51^, integrated their data with MIMIC I, and^52^ combined MIMIC-II with UQVS data. Other studies validated their algorithms across different datasets; ^53^ undertook independent validation with PPG-BP and MIMIC-II, while^54^ used either UCI or UQVS.

Lastly, of the studies reviewed, 68% relied on openly accessible datasets, promoting research advancement and enhancing reproducibility. The remaining 25 studies (32%) employed their own collected data. Among these, only one study^55^ shared its data publicly (SHIMMER database^45^), promoting transparency and data sharing within the scientific community, while the rest had restricted access. Beyond our included studies, further publicly available resources include the ambulatory ECG-PPG dataset by Wong et al.^56^ and the 56-subject PPG database by Argüello-Prada and Castaño Mosquera^57^. These additional datasets, while not part of our meta-analysis, illustrate the growing but still limited availability of open data for cuffless BP research.

### Estimation models and algorithmic approaches

Once the physiological signals are acquired, the next fundamental step is selecting an appropriate modeling strategy to infer blood pressure from these surrogate measurements. Successful cuffless BP estimation relies not only on the sensing technology but also on the methodological framework used to process, transform, and interpret the data to generate BP estimates. Previous works have typically classified BP methods according to the underlying algorithmic framework, distinguishing between hemodynamic, ML, and DL models^20,23,58^. However, we found this classification insufficient to capture the multidimensional landscape of current developments in the field.

**Fig. 4:**
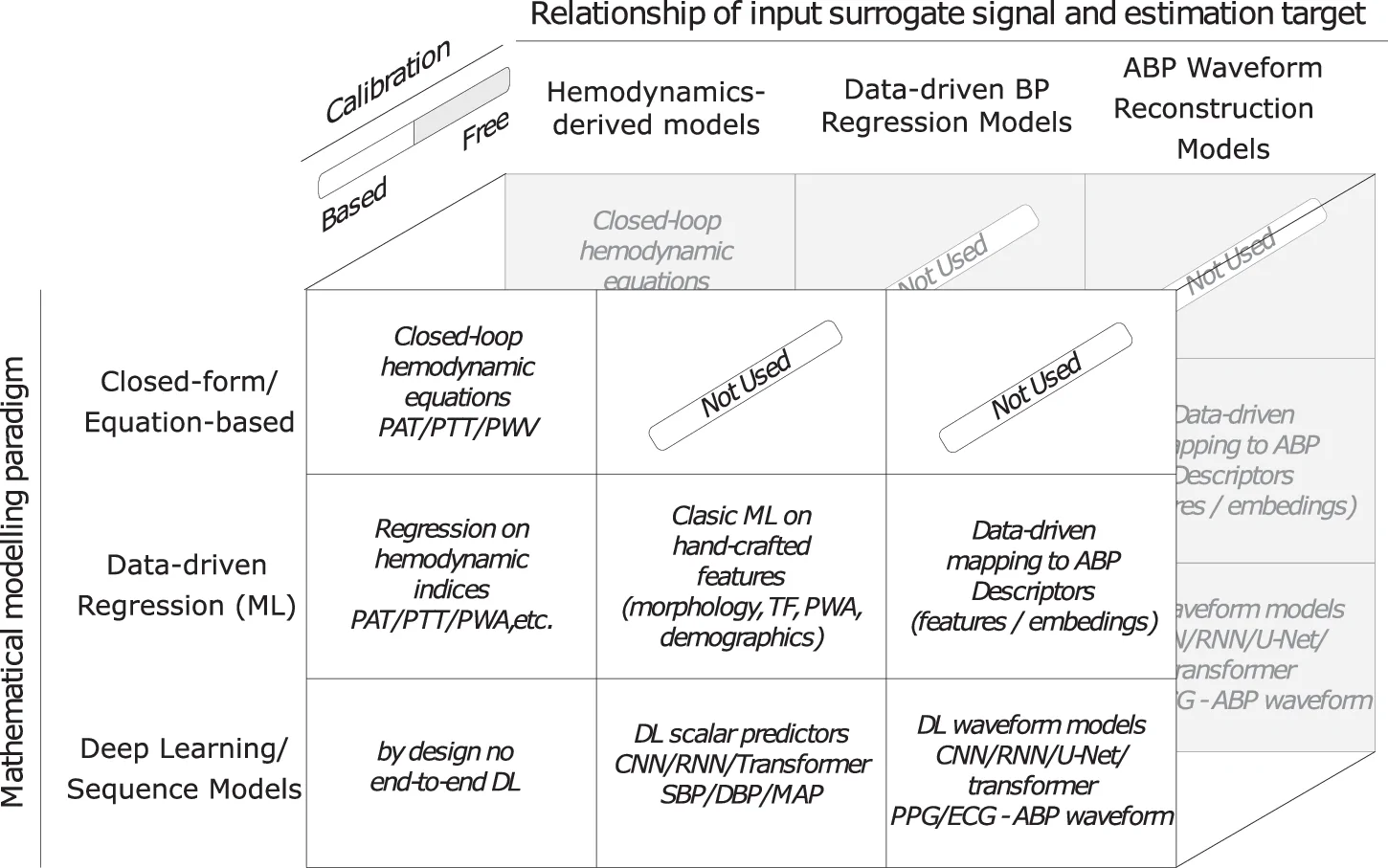
Three-dimensional taxonomy of cuffless BP algorithms. The horizontal axis groups methods according to the relationship between the surrogate signal and the estimation target; the vertical axis groups them by mathematical modeling paradigm; and the depth axis represents the calibration attribute, distinguishing calibration-based and calibration-free algorithms. Closed-form equations appear only within hemodynamics-derived models. Data-driven regression spans all three families, while deep learning models appear in both data-driven BP regression and ABP waveform reconstruction.

To address this limitation, we introduce a taxonomy organized along three orthogonal axes (Fig. 4). The first axis describes the relationship between the surrogate input signal and the estimation target; the second axis defines the mathematical modeling paradigm; and the third axis distinguishes the information used for personalization or calibration. Along the first axis—linking surrogate signals to estimation targets—we propose three categories: hemodynamics-derived models, data-driven BP regression models, and ABP waveform reconstruction models. These categories differ fundamentally in their estimation targets: the first two focus on scalar BP values, whereas the third reconstructs the full ABP waveform. Importantly, the categories are agnostic to the sensing modality; instead, they reflect the expected output of the algorithm. Hemodynamics-derived models use physiology-grounded features such as Pulse Arrival Time (PAT), Pulse Transit Time (PTT), or Pulse Wave Velocity (PWV) to estimate scalar systolic BP (SBP), diastolic BP (DBP), or mean arterial pressure (MAP). Data-driven BP regression models learn a mapping from either raw biosignals or derived features—including morphological and statistical descriptors, time–frequency (TF) representations, pulse-wave analysis (PWA) indices, and demographic variables—to predict scalar BP. ABP waveform reconstruction models are typically end-to-end approaches that take raw or minimally processed PPG, ECG, or iPPG as input and reconstruct the ABP waveform, from which SBP, DBP, and MAP are subsequently derived.

Furthermore, calibration constitutes the third orthogonal dimension that plays a critical role in both methodological design and performance evaluation. Two primary categories can be distinguished: calibration-based and calibration-free algorithms. Calibration-based methods are tailored to individual subjects and require periodic calibration using a conventional cuff-based device. In contrast, calibration-free aims to eliminate dependence on external calibration inputs—making accurate BP estimation more challenging. Calibration-free methods may or may not incorporate demographic information such as age or gender.

Applying this taxonomy to the reviewed literature showed a substantial 73% (*n* = 63 out of 86) focused on data-driven BP regression models, while hemodynamics-derived models were employed in 16% (*n* = 14 out of 86). The remaining 11% (*n* = 10 out of 86) focused on ABP waveform reconstruction models.

Hemodynamics-derived models rely on closed-form equations to fit their coefficients to an expected hemodynamics behavior^59^. Many of these formulations originate from classical physiological models such as the Bramwell-Hill or Moens-Kortweg models^60^, and appear in various forms, including logarithmic^61^, proportional^62–65^, inverse^66,67^, and inverse square^68^ equations. These models are typically calibration-based, requiring an initial calibration and periodic recalibration.

A key aspect of these methods is the use of timing biomarkers such as PAT, PTT, and PWV, all of which demonstrate a correlation with BP variations. PAT is calculated as the time difference between the R-peak of an ECG signal and a fiducial point on the PPG waveform, while PTT is measured between the same fiducial point extracted from two PPG signals recorded at different anatomical sites (Fig. 2). Although PPG is the most common modality, PTT can also be derived from other pulse-wave signals (e.g., IPG, iPPG, PPW), provided they also capture the arterial pulse-wave arrival^48,69^. PWV represents the speed of propagation of the arterial pressure wave and is computed as the arterial path length divided by PTT.

The optimal fiducial point for determining these biomarkers is not clearly defined^70,71^. Common options include the foot, systolic peak, and maximum first derivative^72^, while less frequently used fiducials include the diastolic minimum time, the intersecting target point, and the maximum second derivative on the systolic part. Several methods for fiducial points determiantion has been proposed^73^, with derivative-based pulse wave analysis (PWA) being the most common^74^. The first derivative, known as velocity plethysmogram (VPG), reflects the velocity of blood volume change, while the second derivative, the acceleration plethysmogram (APG), represents the acceleration of the change, which relates to BP gradient. However, this derivative-based approach is highly prone to noise and artifacts, making it essential to remove respiratory-induced artifacts and incorrect beat detection for accurate calculations^75,76^.

Two modeling dimensions coexist across the literature: the biomarker used as input and the physiological formulation used to map this biomarker to BP. Accordingly, diverse hemodynamic models have been reported, including PAT-based^51,59,71,72,77,78^, PTT&PIR-based^79^, PWV-based^32,69,80–82^, PWV&PWA-based^81^, PTT&HR based^55^, Moens-Korteweg equation^32,83^, Windkessel model^26,50,84^, PTT-BP calibration model^85^, Bramwell–Hill model^69^, TPS model^50^ as well as, other customized solutions.

Recent evidence highlights that SBP and DBP often require distinct hemodynamic formulations, as the optimal PAT-based model differs between targets. Likewise, the choice of PPG fiducial point affects SBP and DBP estimation differently, since each fiducial emphasizes different aspects of cardiovascular dynamics^71^. Together, these results underscore the importance of target-specific physiological modeling when using timing biomarkers for cuffless BP estimation.

Data-driven BP regression models utilize a broad set of features to capture complex, nonlinear patterns for BP estimation. These algorithms can extract BP-related variations from biosignals and biometrics, effectively analyzing multi-dimensional physiological data to handle intricate and continuous variations^49,86–89^. Physiological signals contain rich information about cardiovascular dynamics that is often difficult to characterize using traditional hemodynamics models. They exhibit patterns and fluctuations associated with arterial stiffness, vascular compliance, and blood circulation - properties that ML algorithms can exploit to improve BP estimation.

Data-driven BP regression models aim to learn complex, nonlinear relationships between physiological signals and BP. Unlike hemodynamics-derived approaches—which depend on explicit physiological equations—these models rely on statistical learning to extract BP-related information from biosignals and biometrics. Physiological waveforms contain rich information about arterial stiffness, vascular compliance and circulatory dynamics that is often difficult to model analytically but can be effectively captured by ML methods^49,86–89^.

A broad range of handcrafted features have been proposed to characterize the dynamics of the ECG, PPG and other pulsatile signals. Timing descriptors such as PAT, PPT, and PWM^90^ are derived from synchronized ECG and other pulsatile signal. PWA provides morphological features that include amplitude- and intensity-based features, temporal-based features such as pulse width indices and ratios derived from these values^91–95^. Derivative-based representations of PPG, such as VPG and APG, have also been used, to enrich feature extraction ^57,96–98^. These higher-order signals enhance sensitivity to morphology-related variations and can provide complementary information. Complementary frequency-domain features derived from Fourier or wavelet analyses—including semi-classical and Schrödinger-based formulations—capture additional hemodynamic information^99,100^.

Beyond traditional descriptors, growing interest has focused on image-based encodings of PPG segments, including spectrograms, scalograms, visibility graphs and other morphological maps, as well as ABP-derived waveform features, which enable synergy with computer-vision models^101–104^. Graph-based representations, including causal-graph descriptors and topology-aware features, have also been explored to model interactions within cardiovascular signals^105,106^. Many recent studies demonstrate that combining multiple feature families yields more accurate models than relying on a single descriptor set^53,102^.

Remote PPG (rPPG) brings additional methodological considerations, since raw observations consist of images or video frames rather than one-dimensional signals. These approaches typically require region-of-interest selection, pixel aggregation and illumination-artifact removal before feature extraction or direct learning^27,35,107,108^. Data scarcity has motivated the use of generative models, such as GAN-based PPG synthesis^108^, and the development of in silico simulators that generate physiologically plausible PPG sequences conditioned on BP trajectories^84^.

A wide spectrum of ML algorithms has been applied for cuffless BP regression. Classical approaches include support vector regression^53,91,106^, linear and penalized regression models^86,109,110^, Gaussian process regression^57,111,112^, random forests^53,113,114^, and boosting methods^49,53,93,100,105^. Hybrid classification–regression pipelines have also been explored, including k-nearest neighbors or decision-tree classifiers followed by regression refinements^52,115^. Additional approaches include multi-parameter fusion models for PPW-derived features^116^, confidence-aware filtering frameworks^117^ and scenario-adaptive formulations and cardiovascular coupling effects^51^.

DL has become increasingly prominent in recent years because it can automatically extract discriminative features from complex physiological signals, removing the need for manual feature engineering. These models are particularly valued for their ability to learn hierarchical representations and capture both spatial and temporal dependencies, substantially improving the modeling of BP-related dynamics. Reported architectures include artificial neural networks (ANNs)^106,118–120^, CNNs^101,108,121–123^, recurrent neural networks (RNNs)^95,124^, deep neural networks (DNNs)^125–127^, bayesian neural networks^84,107^, feed-forward neural network^99^, bidirectional LSTM (BiLSTM) with attention layer^89^ are extensively adopted. Additionally, hybrid DL models^27,35,51,88,92,102–104,108,122–124,127–139^. Optimization strategies such as AMSGrad and specialized neural topologies like dendritic neural regression have also been reported to refine BP predictions^87^. Transfer learning is widely adopted, with models pre-trained on large public datasets and fine-tuned for personalized BP estimation^89,101,106,124^.

Combining physiological signals with demographic and biometric information (e.g., as age, gender, weight, or BMI) further enhances BP estimation by accounting for inter-individual variability in cardiovascular health ^27,89,118,119^. Notably, recent studies indicate that SBP and DBP rely on distinct physiological descriptors, suggesting that target-specific modeling may be necessary for optimal performance^57,93^.

Overall, data-driven models offer significant advantages for continuous BP monitoring. They can integrate heterogeneous data sources and adapt over time by learning from new observations, allowing them to track evolving physiological conditions and maintain stable performance in long-term use. When embedded into wearable devices, these models enable real-time BP estimation, providing a practical approach for managing hypertension and other cardiovascular conditions outside traditional clinical settings. Despite these benefits, their performance remains highly dependent on data quality and diversity, and their generalizability is often restricted to populations similar to those represented in the training datasets. Moreover, issues of transparency and interpretability are frequently overlooked, limiting the clinical adoption of DL-based methods.

ABP waveform reconstruction models aim to estimate the full ABP waveform, which SBP and DBP can be derived. Recovering the complete waveform provides richer hemodynamic information—including pulse pressure, arterial stiffness, and beat-to-beat variability—which cannot be captured by scalar BP estimation alone. This makes waveform-based approaches particularly valuable for continuous, cuffless cardiovascular monitoring.

A variety of architectures have been proposed for this task. Reconstruction-based models include deep convolutional autoencoders^140^, GRNN-based models^141^, recurrent networks such as LSTMs^90^ and BiGRU-attention variants^142^, as well as temporal convolutional networks and Informer-style long-sequence forecasters^143,144^. A second family of methods adopts generative modeling, including GAN-based formulations^54^ and diffusion models^145^. A third line of research employs attention-based and Transformer architectures^142,146^. Unlike traditional feature-engineering pipelines, these models learn hierarchical representations directly from raw signals, eliminating the need for handcrafted features and enabling the extraction of morphology-rich patterns automatically.

Although these models demand large, high-quality datasets and are sensitive to motion and alignment artifacts, their ability to reconstruct full beat-to-beat waveforms offers richer hemodynamic information than point-wise regression methods, positioning waveform reconstruction as a rapidly advancing component of cuffless BP monitoring.

### Evaluation of BP estimation models

Validation practices varied widely across the reviewed studies, as summarized in Table 2. A significant portion of the publications are subject-specific (*n* = 34 out of 86; 39.1%), highlighting the need for standardized validation methodologies to assess potential issues such as data leakage, bias, and generalization. In calibration-free methods (*n* = 44 out of 86; 50%), generalization is expected, however, subject-wise splits and subject-wise leave-out-cross validation results should be presented carefully. Moreover, hold-out methods should be clearly reported to contextualize the generalizability of the results.

Several validation techniques have been reported in BP estimation studies, with hold-out methods being the most frequently employed (*n* = 48 out of 86; 55.2%), followed by cross-validation (CV) approaches, including k-fold, leave-one-out CV (LOOCV), and nested CV (*n* = 38 out of 86; 44%). Prior to 2023, these methods were more evenly distributed. However, since 2023, CV-based strategies have become more prevalent (*n* = 22 out of 43; 42.3%), reflecting a shift toward more rigorous and generalizable evaluation practices. However, ensuring proper subject stratification remains crucial to prevent information leakage and reduce bias.

Notwithstanding, 34 publications conducted subject-wise validation. Of these, 7 LOOCV, 2 used leave-one-out, 13 applied hold-out validation, 9 used CV, and 4 performed subject-specific calibration. This indicates that 65.8% (48 out of 73) of studies performing CV or hold-out methods did not employ subject stratification. This might generate better-performing models, but in such scenarios, information leakage introduces bias, which is why the results cannot generalized. Furthermore, 12 publications did not report the ratio of their train-test split, 4 performed random splitting, and 13 applied a sample-wise split rather than a subject-wise approach. Additionally, 4 publications lacked sufficient detail about their data splitting strategy, introducing ambiguity regarding the validity of their evaluation methods.

Authors ^34,50,51,79,81,85,91^ performed subject-stratified calibration steps for the evaluation of their proposed method. Guo et al.^83^ validated their novel calibration-free, subject-wise approach by comparing its BP values with those acquired from a reference aneroid sphygmomanometer. Nonetheless, this method is not suitable for real-time or continuous monitoring. Authors ^32^ used correlation analysis and individual estimation to validate their methodology.

Furthermore, for calibration-based algorithms it is imperative to conduct static and dynamic tests. Static tests assess the baseline conditions, whereas dynamic tests ^72^ replicate real-world BP variation, guaranteeing the model’s flexibility. Publications such as, ^51,79,81,85,89,91,109^ conduct both the tests. Additionally, some works^26,49,79,85,87,89,91,116,127,147,148^ have started to conduct long-term studies, ranging from a few days to months, to evaluate the stability and reliability of their methods. Such comprehensive validation techniques ensure a detailed understanding of the model’s performance in real application. In calibration and recalibration scenarios, it is important to highlight that validated oscillometric devices are typically used, as manual auscultation is impractical. Given the variability in readings between oscillometric device brands and models, and the fact that calibration-based algorithms track BP changes relative to the calibration point, the choice of calibration device significantly impacts BP estimation accuracy ^149^. Moreover, for calibration-free algorithms validation among non-normotensive populations crucial to ensure effectiveness across diverse BP ranges. However, despite claims of superiority, several studies researches ^77,91,99,116,141^ fail to demonstrate applicability across all populations.

Beyond the choice of validation methodology, the standards and performance metrics used to assess BP estimation algorithms are equally critical. There are well-established standards for evaluating automatic cuff-based BP measurement devices, including the American National Standards Institute (ANSI)/American Association for the Advancement of Medical Instrumentation (AAMI) SP 10-1987 standard^150^, IEEE Standard 1708a-2019^151^ and British Hypertension Society (BHS) protocol^152^, as shown in Fig. 2c. These standards clearly define the criteria for reporting and visualizing results. ANSI/AAMI recommends reporting mean deviation ± standard deviation (*MD* ± *SD*), whereas the BHS protocol emphasizes the cumulative error percentage (*CP*) at 5, 10 and 15 mmHg (*CP*_5_, *CP*_10_, and *CP*_15_). To provide a consistent and comparable evaluation, IEEE Standard 1708-2014 introduced a new grading system based on mean absolute difference (*MAD*), consistent with AAMI and BHS standards reporting. It recommends using multiple metrics, including *MAD*, mean absolute percentage difference (*MAPD*), *MD* ± *SD*, *CP*_5_, *CP*_10_, and *CP*_15_.

Additionally, IEEE Standard 1708a-2019^151^ recommends using graphical tools such as Bland-Altman plots, scatter plots, and BP histograms. Bland-Altman plots are vital for identifying systematic biases and defining limits of agreement, ensuring cuffless BP readings align with standard cuff-based measurements. Scatter plots of predicted versus true BP values visualize model accuracy and highlight potential inconsistencies or biases. Moreover, separate histograms of BP changes and population demographics provide crucial insights into model performance. Histograms of BP changes illustrate the distribution and stability of BP readings over time, showing how well the device tracks fluctuations in BP. Meanwhile, histograms of population demographics, such as age, gender, or health status, reveal whether the cuffless BP estimation models generalize well across different groups. This distinction ensures that the device performs accurately and consistently, not only within specific demographic groups but also across a broad patient population. Studies such as^149^ highlight the importance of these visualizations in validating both the accuracy and clinical applicability of cuffless BP devices. However, these protocols are designed specifically for validating automatic cuff devices and are unsuitable for cuffless BP devices. Cuffless devices present unique validation challenges, which have only recently been acknowledged in the field^149,153^.

**Fig. 5:**
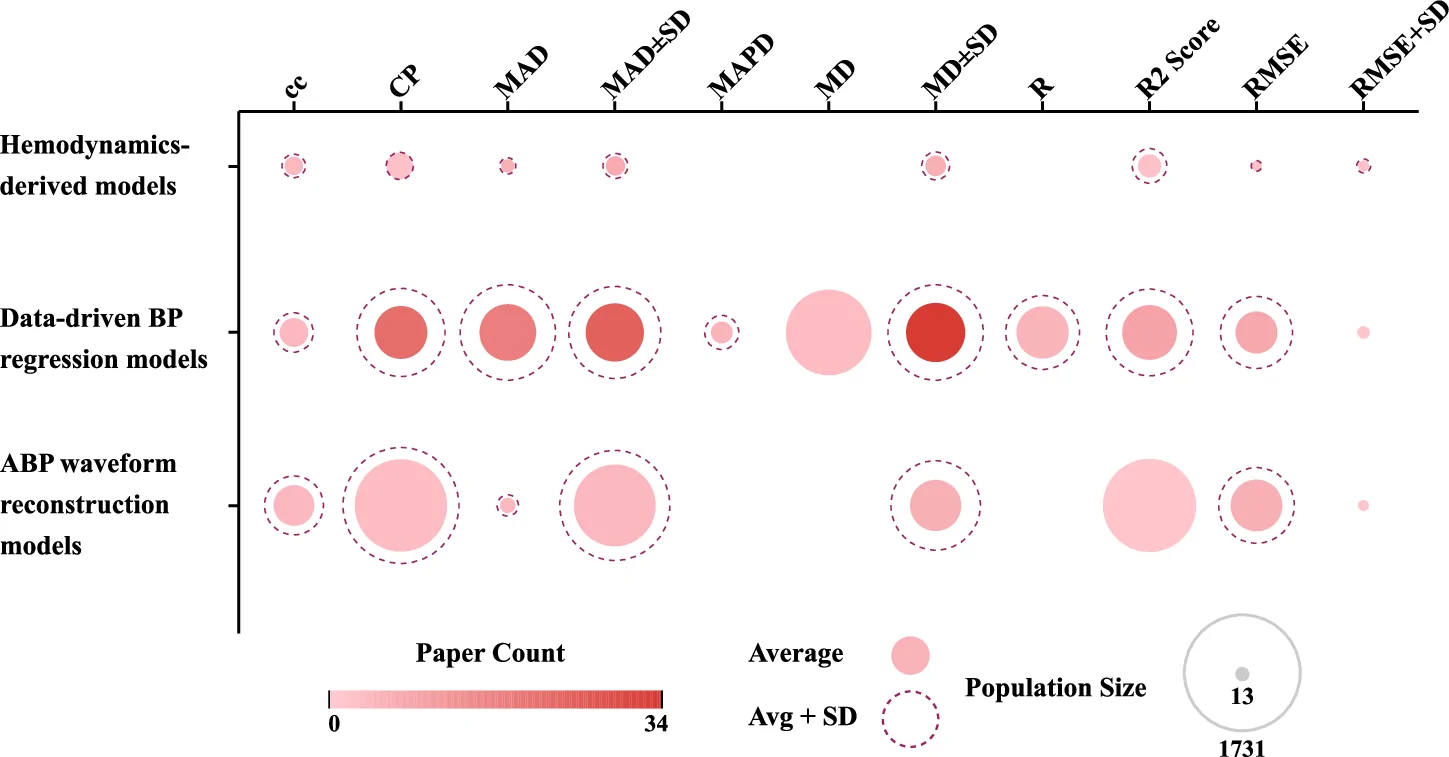
Summary of evaluation metrics in BP studies. Highlighting the missing consistency in the reporting of BP studies. Circle size represents the population size, indicating the average number of subjects or test sets in each study. Color intensity indicates algorithm usage frequency.

**Fig. 6:**
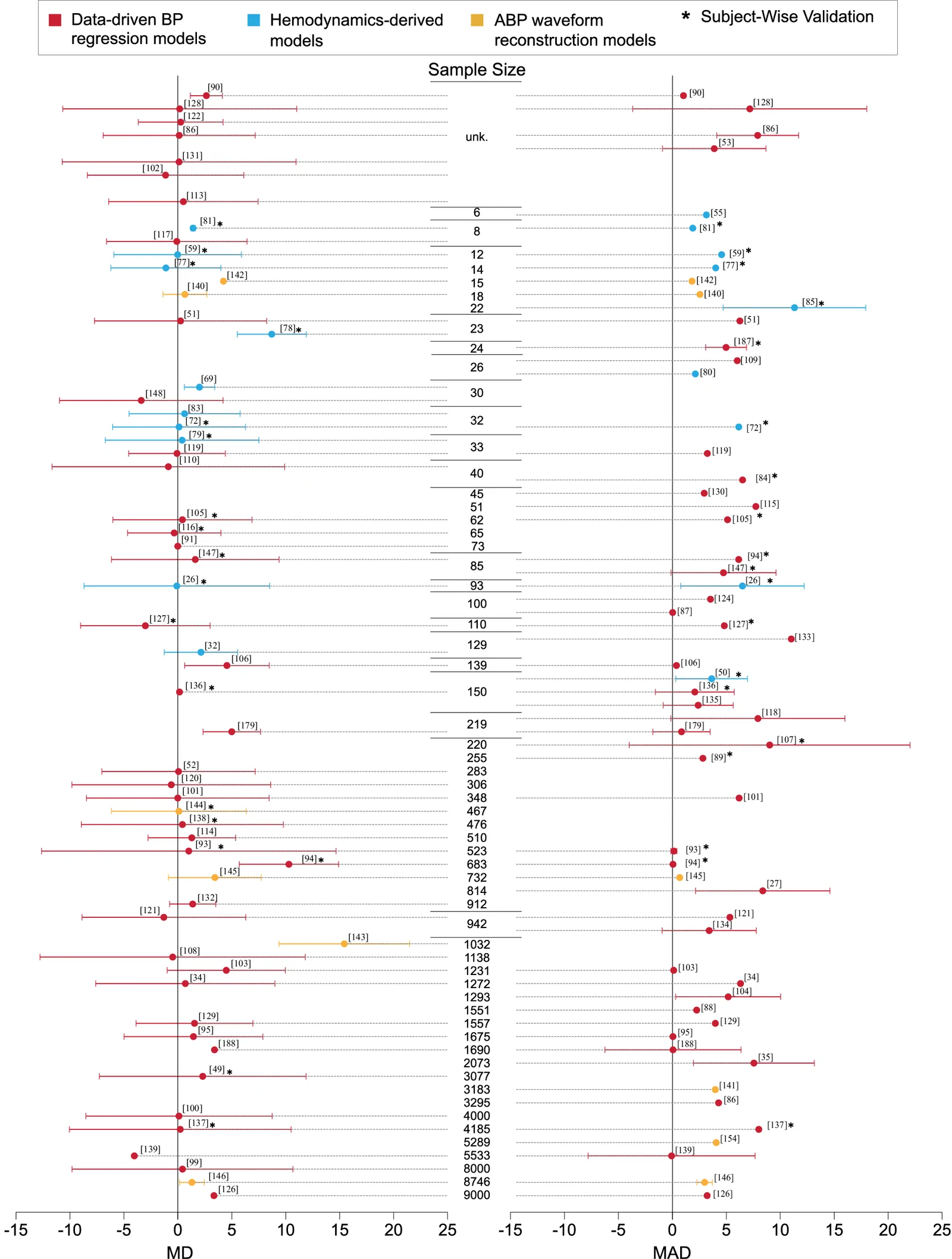
Performance in blood pressure studies. Summary of reported performance metrics considering mean error (MD) and mean absolute error (MAD) with standard deviation (SD) for systolic blood pressure (SBP). The *y*-axis represents the training set sample size, while colors correspond to the algorithm type. Star markers denote models that were validated using subject-wise evaluation methods.

Figure 5 illustrates the evaluation metrics reported across the review studies, grouped by algorithm category. The color intensity of each circle is directly proportional to the number of publications utilizing each metric. Furthermore, the size of each circle represents the average population size. If a subject size or test size is not provided, a default value of 1 is assumed. As shown in Fig. 5, the most frequently used metrics are *MAD* and *MD*, often accompanied by their associated *SD*. Figure 6 provides a complementary view by displaying the distribution of SBP errors reported in each publication. The x-axes correspond to *MD* and *MAD*, while the error bars reflect the resorted variability (*SD*). The y-axis denotes the training set sample size, allowing visual comparison of model performance relative to dataset scale. Additionally, color indicates the category of BP estimation method. Out of 87 identified publications, 64 have reported the *MAD* values for both SBP and DBP. Additionally, 53 publications have reported only *MD* ± *SD* for both SBP and DBP except^126^ (authors only reported *MD*, not the *SD*). Furthermore, 37 publications have reported both *MAD* and *ME* ± *SD* except^126^ for both SBP and DBP.

Although many works,^27,52,55,59,69,77–80,83,85–88,90,95,99,101,113,114,116–119,123,126,128,130,134,136,140–142,148,154^ have correctly reported current suggested evaluation metrics, their methodologies are not robust enough due to lack of diverse BP cohort, missing to report underlying population distributions or not satisfying amount of required subjects recommended in the IEEE standard. Despite clearly established guidelines of reporting as per the IEEE standards and others, many of the studies^33,53,54,72,81,84,91^ failed to provide comprehensive evaluation metrics and did not fulfill the aforementioned standards and/or model evaluation. Some studies have inconsistently reported adherence to standards such as AAMI, with several papers misreporting key metrics like *MAD* and *MD*. For example, several works used *MAD* ± *SD* instead of *MD* ± *SD*, or cited incorrect thresholds for AAMI compliance^53,130,131,141,154^. This highlights the need for greater consistency and accuracy in reporting evaluation metrics in the field. Furthermore, although many studies included Bland–Altman plots as part of their evaluation, none of them reported verifying whether the differences between reference and estimated BP values followed a normal distribution—an essential assumption for the correct application of Bland–Altman analysis. The omission of this verification step raises concerns about the validity of the agreement analysis presented in these works.

Direct comparison of reported performance across studies is not feasible: population characteristics, sensing modalities, preprocessing pipelines, calibration strategies, and validation schemes vary substantially across publications. This heterogeneity fundamentally biases error metrics and makes them non-comparable. Accordingly, we perform a meta-analysis to contextualize reported results and assess robustness across diverse methodological settings.

Table 3 shows that the pooled SBP *ME* across all validation types is small and positive (1.12 mmHg; 95% CI [0.76, 1.47]), indicating minor average overestimation. Despite the small average signed bias, the very high heterogeneity (*I*^2^ = 98.8%) indicates that reported signed-bias estimates vary substantially across studies. The pooled *ME* should therefore not be interpreted as a representative performance benchmark for any specific clinical use case; rather, the meta-analytic framework is used here to quantify cross-study inconsistency and to identify validation design as an important source of heterogeneity. In practice, this means that the current literature does not yet support strong claims of consistent or clinically meaningful performance.

**Table 3:** Summary of meta-analytic pooling of SBP mean error (ME, mmHg)

Model/Subgroup	*k*	Method	Pooled ME	95% CI	*τ*^2^	*I*^2^ (%)
*Primary estimate*						
Overall (all studies)	46	DL RE	1.12	[0.76, 1.47]	0.91	98.8
*Subgroup analyses*						
Subject-wise only	25	DL RE	0.81	[0.25, 1.36]	1.27	96.3
Record-wise only	17	DL RE	1.50	[0.43, 2.57]	3.83	99.4
RoB Overall: Unclear	23	DL RE	0.39	[−0.16, 0.94]	1.20	90.8
RoB Overall: High	23	DL RE	1.68	[0.84, 2.51]	2.95	99.3
*Sensitivity analyses*						
REML + HK (robust)	46	REML + HK	0.86	[−0.08, 1.79]	8.28	98.8
Trimmed (∣ME∣≤5 mmHg)	44	DL RE	0.57	[0.32, 0.82]	0.31	96.6
Winsorised (5 mmHg cap)	46	DL RE	0.79	[0.54, 1.05]	0.38	97.1
PET–PEESE adjusted	46	Regression (HC3)	0.76	[−0.12, 1.64]	—	—

Positive values indicate overestimation.

*DL* DerSimonian–Laird random effects, *HK* Hartung–Knapp, *REML* restricted maximum likelihood, *RoB* risk of bias.

**Fig. 7:**
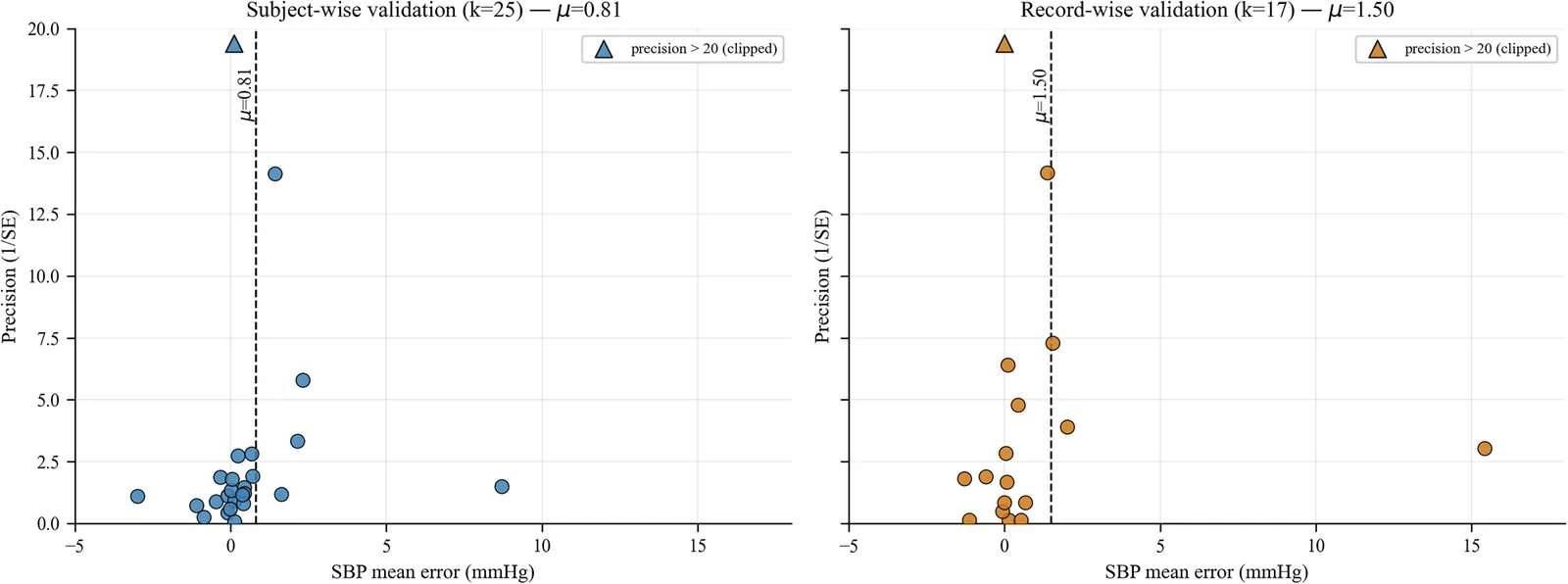
Study effect analysis. Funnel plots of study effects for SBP mean error (ME), shown with identical axes. Each point represents a study’s mean error plotted against its precision (1/SE).

In particular, subject-wise studies exhibit a smaller and more stable signed bias (*μ* ≈ 0.81 mmHg, *τ*^2^ = 1.27), whereas record-wise studies show a larger mean signed bias (*μ* ≈ 1.50 mmHg, *τ*^2^ = 3.83) and substantially wider dispersion (Table 3; Fig. 7, left vs. right panel). This pattern is consistent with potential information leakage or overlap between training and test subjects and provides empirical support for our proposal that subject-wise validation should be treated as the minimum acceptable standard, because record-wise evaluation may yield overly optimistic estimates of generalization performance.

**Fig. 8:**
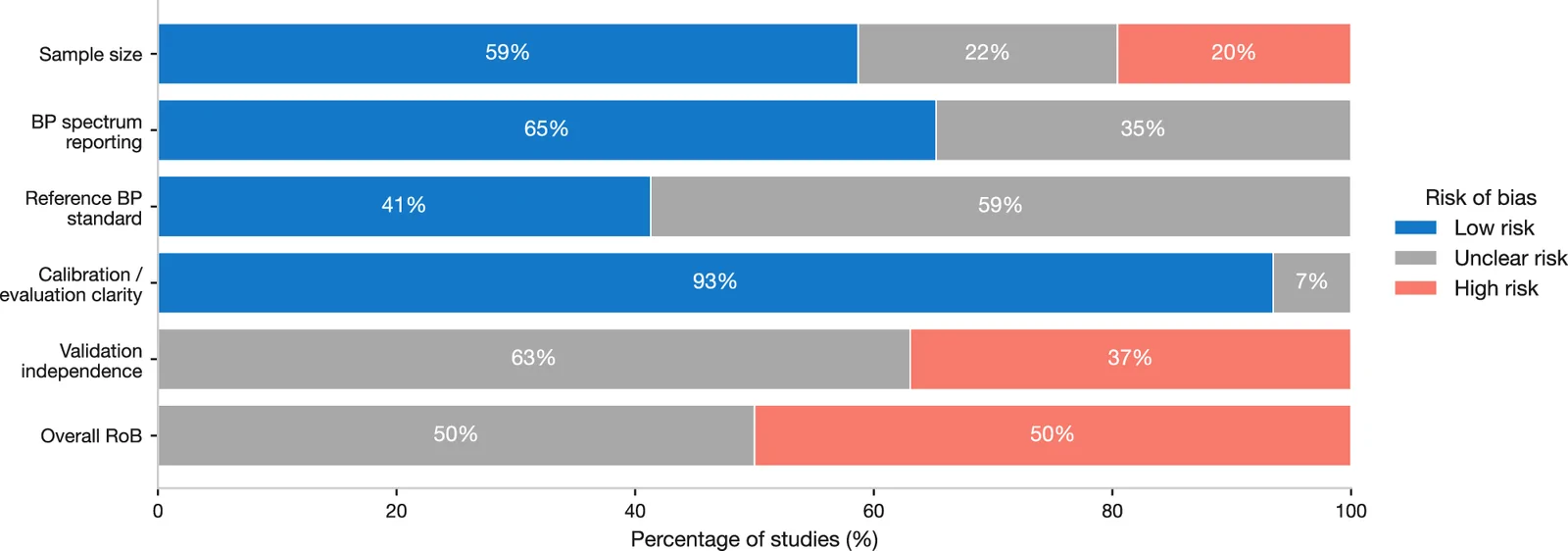
Review bias assessment. Distribution of risk-of-bias ratings by domain. Overall, RoB was assigned using the worst-domain rule; no study achieved a low overall RoB.

To formally characterize the methodological sources of this heterogeneity, we developed a structured five-domain risk-of-bias (RoB) framework adapted from QUADAS-2^155^ and PROBAST^156^. The framework covered sample size adequacy, BP spectrum reporting, reference BP standard, calibration/evaluation clarity, and validation independence. Each study was rated as Low, Unclear, or High risk of bias in each domain, with overall RoB determined by the worst-domain rule. The distribution of domain-level and overall RoB ratings is shown in Fig. 8. Validation independence was the most problematic domain: no study achieved low risk in this domain, while 63% were rated as unclear risk and 37% as high risk. Consequently, no study achieved low overall RoB, reflecting the absence of fully independent external validation cohorts with complete methodological reporting. This converts the previously qualitative concerns regarding sample-wise splitting, unclear validation, and non-subject-wise evaluation into a structured study-level assessment. Stratification by overall RoB further supports this interpretation (Table 3): studies rated at high overall risk of bias yielded a pooled SBP ME of 1.68 mmHg [0.84, 2.51], compared with 0.39 mmHg [−0.16, 0.94] for studies rated at unclear risk. The point estimate was therefore more than four-fold higher in high-RoB studies, with only limited CI overlap, suggesting that a weaker methodological design is associated with larger signed bias.

Trimming and Winsorising indicate that a small number of extreme ME values disproportionately influence pooled estimates, with the central tendency remaining below 1 mmHg when outliers are down-weighted. Notably, all sensitivity analyses, including the conservative REML + HK and the bias-corrected PET–PEESE estimate (0.76 mmHg), are directionally consistent with the primary estimate and do not change its sign or clinical interpretation. These results further illustrate the instability of summary error estimates under inconsistent reporting and heterogeneous study design, and reinforce the importance of standardized reporting of MD ± SD, *MAD*, *CP*_5_, *CP*_10_, *CP*_15_, *MAPD*, and sample size.

Bias diagnostics (Table 4) show a borderline Egger intercept (*p* = 0.069) with no statistically significant funnel asymmetry. PET regression does not detect small-study effects (*p* = 0.284), and the PEESE-adjusted estimate of 0.76 mmHg is *lower* than the unadjusted pooled estimate, indicating that any small-study effects modestly inflate rather than substantially distort the headline result. Consistent with the funnel plots stratified by validation type (Fig. 7), these findings indicate, at most, modest small-study effects rather than strong evidence of systematic publication bias.

**Table 4:** Publication-bias diagnostics for SBP mean error (ME)

Bias test	Model	*k*	*p*-value	Interpretation
Egger regression (HC3)	Standardized ME ~ precision	46	0.069	No significant funnel asymmetry (borderline)
PET regression (HC3)	ME ~ SE	46	0.284	No small-study effect detected
PEESE regression (HC3)	ME ~ SE^2^	46	0.709	Bias-adjusted mean: 0.76 mmHg

HC3: heteroscedasticity-robust covariance (MacKinnon–White correction).

The absence of strong evidence for classical publication bias suggests that the main limitation is not selective publication of positive findings, but rather the prevalence of small, underpowered, and methodologically heterogeneous studies. This directly motivates our proposal for standardized reporting. Overall, at a minimum, studies should clearly describe the dataset and population, specify the validation protocol (e.g., subject-wise vs. record-wise), and report standard performance metrics with uncertainty. Systematic reporting of these items would reduce optimistic interpretation, make cross-study comparisons more transparent, and provide a more reliable basis for judging whether a model’s performance is likely to generalize beyond the original cohort.

## Discussion

Despite reported errors overlapping with cuff-based validation thresholds, current cuffless BP systems remain fragile in real-world settings. Many studies rely on non-ambulatory data, narrow BP distributions, or evaluation choices that risk data leakage and optimistic bias. Such data, which are often ICU, laboratory, or static seated-rest recordings, do not capture the motion, posture changes, behavioral factors, and autonomic fluctuations that dominate daily life. Weber-Boisvert et al.^157^ showed that PPG morphology and feature–BP relationships differ substantially between ICU and controlled environments, leading to sharp drops in cross-dataset performance when models are transported across settings. Similarly, Cisnal et al.^93^ quantified a marked performance gap between in-clinic and ambulatory recordings on the Aurora-BP cohort: despite robust Markov-blanket feature selection and nested CV, ambulatory models exhibited significantly higher errors and frequently failed to meet IEEE/AAMI criteria.

These findings suggest that the performance gap is not only driven by differences in protocol realism, but also by a breakdown of the physiological assumptions underlying cuffless BP estimation. Most approaches rely on surrogate signals such as PPG, which measure peripheral blood volume rather than arterial pressure, leading to an indirect and non-unique mapping to BP^158–160^. In ambulatory settings, this relationship becomes highly non-stationary due to motion artifacts^161^, sensor contact variability^162^, and hydrostatic effects^163–165^, which alter waveform morphology and therefore degrade the reliability of PWA-based features, independently of systemic BP. Simultaneously, autonomic regulation during daily activities induces rapid changes in vascular tone, altering peripheral resistance in ways that are only partially reflected in PPG, as it captures microvascular dynamics influenced by both local and systemic mechanisms^166–168^. In addition, timing-based biomarkers such as PAT are further confounded by variations in pre-ejection period (PEP), which varies with sympathetic activity and breaks the assumed monotonic relationship with BP^169,170^. These effects collectively violate the core assumptions of both hemodynamic models (stable physiological relationships) and some data-driven models (stationary signal–BP mappings), leading to domain shift and reduced generalizability, consistent with the degradation observed when models trained on controlled datasets (e.g., MIMIC) are applied to ambulatory cohorts^171,172^. In line with these findings, real-world reliability remains limited—particularly in ambulatory, hypertensive, elderly, and medicated cohorts—so current systems cannot yet be considered medical-grade without rigorous, standard-conformant validation across diverse populations and conditions.

**Fig. 9:**
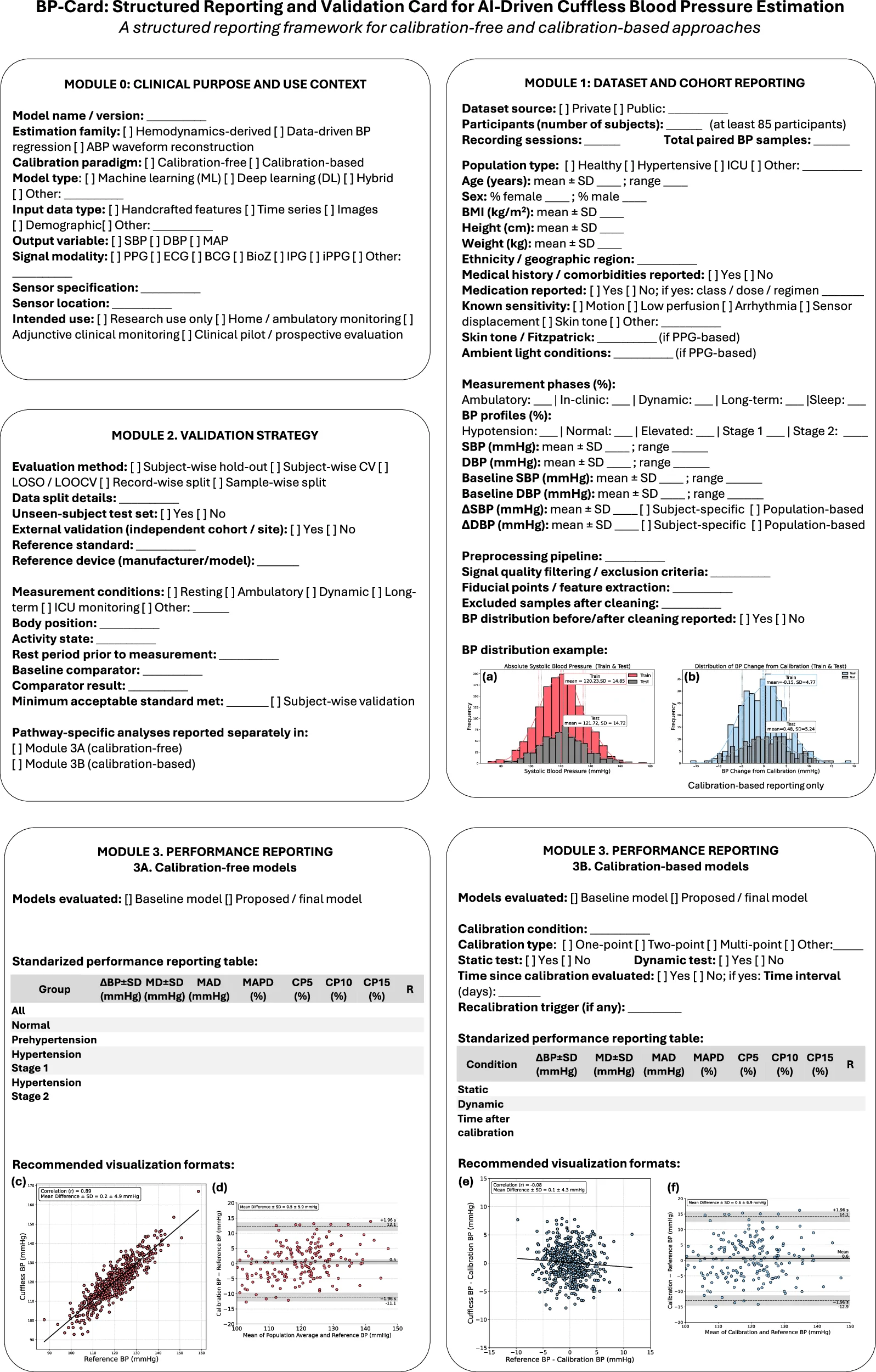
Generic BP-Card template for standardized reporting of cuffless BP estimation studies. Module 0 defines the clinical purpose and intended use context. Module 1 specifies dataset and cohort reporting requirements, illustrated by **a** overall BP distribution and **b** BP change from calibration. Module 2 summarizes validation strategy reporting requirements. Module 3A provides a standardized performance reporting format for calibration-free models, with **c** scatter and **d** Bland–Altman plots for absolute BP agreement. Module 3B provides the corresponding format for calibration-based models, with **e** scatter and **f** Bland–Altman plots for BP change relative to calibration measurements^149^.

**Fig. 10:**
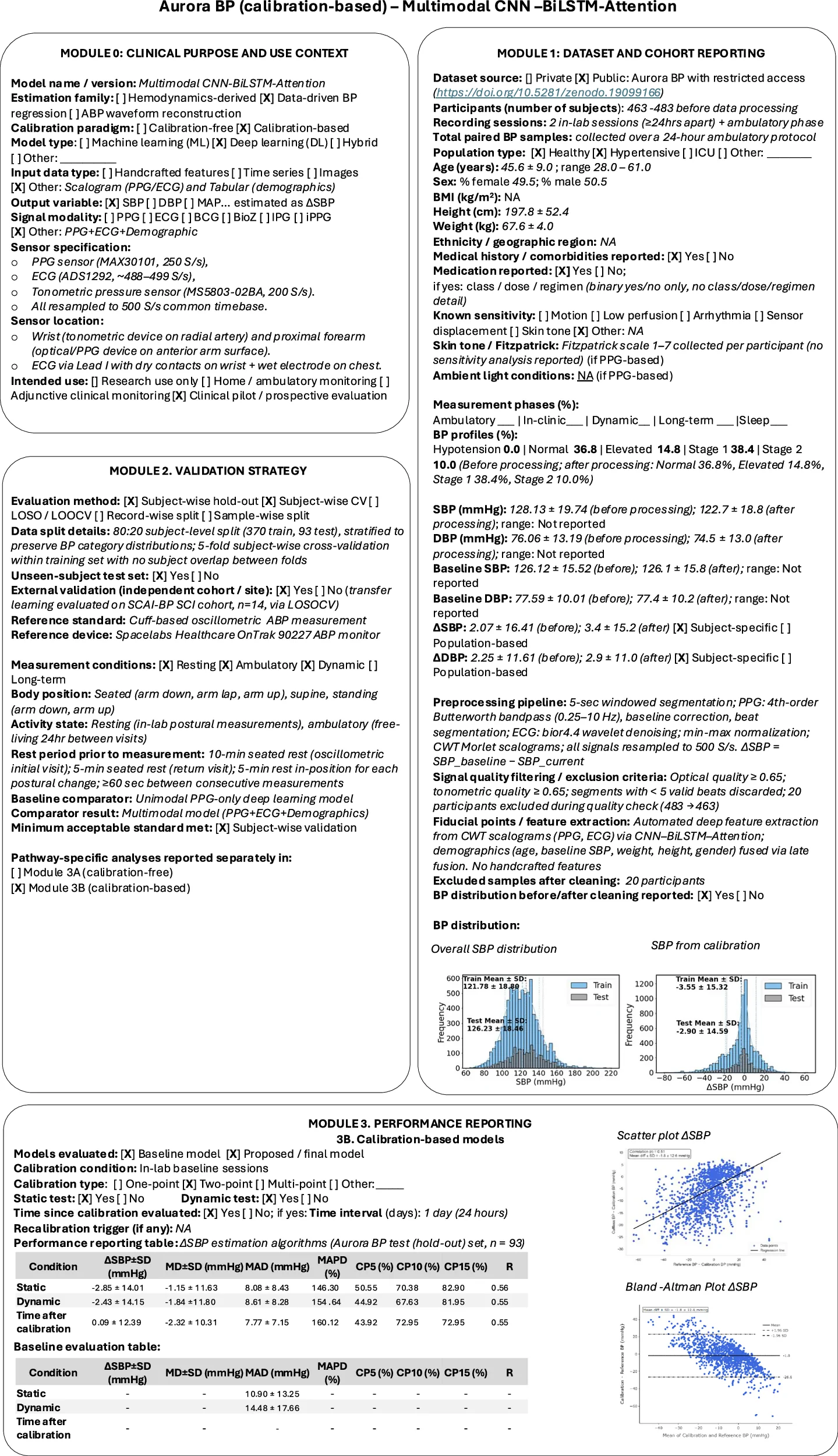
Worked example (based on ref. ^178^) of the proposed BP-Card reporting framework applied to a calibration-based cuffless BP model using the Aurora-BP dataset. The figure illustrates how structured reporting captures clinical purpose and use context (Module 0), dataset and cohort characteristics (Module 1), validation strategy (Module 2), and calibration-specific performance evaluation (Module 3B). Included visualizations show train/test ΔSBP distributions, ΔSBP change from calibration, and agreement analysis using scatter and Bland--Altman plots.

In this context, inconsistent reporting of subject characteristics, lack of standardized metrics, and insufficient validation protocols severely limit the broader validity and comparability of current models. To address these gaps, we consolidate our findings into a structured BP-Card, a model-card-inspired framework that operationalises reporting and evaluation requirements for cuffless BP machine learning studies^173^. The BP-Card is designed to complement the proposed three-axis taxonomy: whereas the taxonomy classifies methods according to estimation family, modeling paradigm, and calibration strategy, the BP-Card translates these distinctions into structured reporting requirements. In particular, the estimation family and modeling paradigm define the methodological identity and intended use of the approach, while the calibration axis determines the appropriate validation and reporting pathway, including the distinction between calibration-free and calibration-based evaluation. The BP-Card is organized into modules covering clinical purpose and intended use, dataset and cohort characteristics, validation strategy, and standardized reporting for both calibration-free and calibration-based models, with explicit attention to bias, fairness-relevant cohort reporting, and equity. Figure 9 summarizes the framework as a generic template for future studies, and Fig. 10 demonstrates its application to a real-world device. Below, we describe each component in detail. Full BP-Card templates for both calibration-free and calibration-based models are provided in Supplementary Figs. S1 and S2.

For cuffless BP systems, intended use strongly affects both the appropriate study design and the interpretation of reported performance. Studies should therefore clearly state the estimation family, modeling paradigm, input and output variables, signal modality, sensor placement, and intended deployment context, such as research use only, home or ambulatory monitoring, or adjunctive clinical use. This information is essential to distinguish fundamentally different modeling objectives and to assess whether the reported validation strategy and performance claims are appropriate for the proposed application. In this sense, Fig. 9 Module 0 operationalises the first two taxonomy axes by documenting what type of method is being studied and in what clinical context it is intended to be used.

Dataset characteristics are a primary determinant of the reliability and generalizability of cuffless BP estimation models. Across the reviewed literature, variability in sample size, BP distribution, and cohort composition substantially limits the comparability of reported results. Proper documentation of the dataset, including sample size and BP distribution, is critical for evaluating model performance. For calibration-based algorithms, documenting BP changes from the calibration point is equally important. Demographic information (age, sex, and medical history) should also be included to better understand the study cohort. Furthermore, post-data cleaning details, such as the amount of removed data or BP distribution before and after cleaning, are often omitted. Clear reporting of cleaning details is essential for reliable comparisons, especially in continuous ABP monitoring with highly variable signal quality. Standardized reporting is essential to advance the field. Fig. 9 Module 1 summarizes these dataset and cohort reporting requirements within the proposed BP-Card. The effect of anti-hypertensive drugs on BP measures validity with cuffless systems has never been investigated and requires dedicated studies and transparent reporting as the main beneficiary users of the technology. In particular, the ongoing medications which affect BP levels, such as diuretics, beta-blockers, angiotensin converting enzyme inhibitors, angiotensin II antagonists, mineralocorticoid drugs, alpha blockers and antagonists ideally should be reported inclusive of total daily dosage and regimens. Recent studies have highlighted that beyond dataset size and diversity, the choice and extraction of physiological features strongly influence performance. In the systematic analysis of PPG features in^174^, it was demonstrated that clear correlations with BP dynamics, underscoring the need to standardize feature-level reporting alongside dataset descriptors.

Validation is a central determinant of whether cuffless BP algorithms can be trusted. Across the reviewed literature, study sizes range from single-subject to cohorts with hundreds of participants, leading to substantial variability in robustness and generalizability. Both calibration-free and calibration-based models face reliability concerns: the former often lack validation across diverse BP distributions, and the latter frequently neglect significant BP variations from the calibration point. This heterogeneity is reflected in the wide dispersion of reported accuracy metrics (e.g., *MAD*: 0.018–11.3 mmHg, *MD*: −3.38 to 15.42 mmHg), underscoring the urgent need for standardized validation and transparent validation protocols. Calibration-based algorithms should not be evaluated solely under static conditions shortly after calibration. If models are calibrated and tested on similar resting measurements from the same individual, the evaluation does not reflect clinical use. Instead, validation must include dynamic conditions that induce BP changes from the calibration point, as well as assessments after a temporal delay to capture drift and physiological variability. IEEE Std 1708a-2019^151^ recommends inducing BP changes from the calibration point within predefined ranges: [−30, 30] mmHg for SBP and [-20, 20] mmHg for DBP. The total number of data points for accuracy analysis is calculated as: *n*_total_ = 255 + (3 × *n*_add_), where *n*_add_ = *n* − 85 represents the extra subjects required due to correlation effects in Phase 1. Each additional subject contributes 3 measurements, increasing the total sample size. Dynamic tests may involve altering device positioning, administering medications, sleep-wake monitoring, or following exercise^175^. In contrast, calibration-free algorithms are designed to generalize across subjects without calibration. Instead of dynamic or long-term evaluations, they require datasets covering a broad BP spectrum. According to IEEE Std 1708a-2019^151^, this includes at least 85 subjects, with a distribution of 21 subjects each for normotension, prehypertension, stage 1 HTN, and stage 2 HTN. Across both paradigms, evaluations should prioritize subject-level metrics to reduce bias and enhance generalizability, as sample-level metrics may disproportionately favor subjects with more data. LOOCV further ensures robust assessment by testing models on unseen individuals.

While CV is useful during development, blind testing on independent datasets is crucial for evaluating real-world generalizability, especially in continuous ABP monitoring, where dynamic conditions and noise may impact accuracy. The evaluation methodology (e.g., k-fold CV, train-test split) must be clearly defined. Finally, recent consensus recommendations for PPG-based BP estimation emphasize harmonized validation frameworks, including transparent reporting of preprocessing pipelines, fiducial point selection, and evaluation protocols^176^. Integrating such signal-level guidance into broader IEEE or ISO-aligned validation standards would substantially improve methodological consistency across the field.

Additionally, the evaluating of baseline models is highly recommended. For calibration-based approaches, baseline predictions are typically obtained using each subject’s calibration BP value, whereas calibration-free approaches uses the population-average BP values and/or demographic information when such variables are included in the model^149^. This comparison is particularly important because BP variability differs substantially across datasets. Models evaluated on datasets with narrow BP distributions may achieve apparently good error metrics despite limited generalization capability. Baseline comparisons therefore help determine whether the reported performance truly reflects the contribution of physiological signal processing. This issue has been emphasized in analyses of the AuroraBP project^48,153^, showing that the absence of baseline comparisons can lead to misleading interpretations of model performance. Therefore, reporting baseline model results provides a more transparent and rigorous assessment of BP estimation accuracy and clarifies the added value of the proposed methodology. To support consistent reporting, Fig. 9 Module 2 provides a table with the essential data elements needed to fully describe the validation strategy.

Adherence to established performance metrics advocated by the BHS, IEEE, and ANSI/AAMI is essential for evaluating BP estimation algorithm. The advantage of employing MD ± SD is that it incorporates all measurement results, but it can be influenced by outliers. Conversely, cumulative percentages are less influenced by outliers, but could yield misleading results in the presence of substantial systematic differences. To address this, the IEEE proposed using *MAD* alongside established metrics. However, the choice of the metric should be distribution-aware. *RMSE* is the maximum-likelihood and most informative summary when residuals are approximately Gaussian, whereas *MAD* is optimal under Laplacian (heavy-tailed) errors. If residuals depart markedly from both, robust alternatives or transformations are preferable^177^. Additionally, *MAPD* was also proposed to account for variability at higher BP levels, which inevitably leads to larger errors. Using relative differences helps reduce the impact of BP level on measurement accuracy. Although, many reviewed articles present *MAD* ± *SD*, this practice is redundant, as *MAD* already captures the average deviation between predicted and observed values. Hence, for a comprehensive presentation of results, it is advisable to include *MD* ± *SD*, *CP*_5_, *CP*_10_, *CP*_15_, *MAD*, *MAPD* and *R*. None of the reviewed studies provided all the aforementioned metrics simultaneously. Without diligent adherence to a standardized reporting protocol, comparative analysis and benchmarking of existing algorithms become markedly challenging.

In the proposed BP-Card, Fig. 9 Module 3A summarizes performance reporting for calibration-free models, including a standardized reporting table and recommended visualization formats for absolute BP agreement in (c) and (d). 9 Module 3B summarizes performance reporting for calibration-based models, including a standardized reporting table and recommended visualization formats for calibration-relative agreement in (e) and (f). For calibration-free algorithms, both overall performance and performance across BP groups should be reported. For calibration-based algorithms, performance should be reported under static and dynamic conditions and, where applicable, across time since calibration. Baseline results should also be included in both modules to support transparent comparison.

Appropriate visualization is essential for comprehensive and transparent BP estimation performance. For dataset reporting, histograms are particularly effective for illustrating the distribution of BP baseline in the study cohort as depicted in Fig. 9a. These histograms should include both the training and test set distributions, clearly highlighting the mean ± SD for each. Calibration-based studies should also include histograms, as shown in Fig. 9b, which depicts BP changes from calibration for both the training and testing sets^151^. To visualize results, we recommend employing Bland-Altman and scatter plots, as per IEEE standard^151^. The Bland-Altman plots illustrate the difference between cuffless and reference BP against their mean, and require explicit verification that these differences follow an approximately normal distribution. Additionally, the limits of agreement should be accompanied by their corresponding 95% confidence intervals (95% CI) to properly quantify the uncertainty around the mean difference and the agreement boundaries. Scatter plot, shown in Fig. 9c illustrates the relationship between changes in cuffless BP and reference BP relative to calibration, emphasizing individual variability in response to calibration. Figure 9d examines BP predictions using population average BP, also excluding cuffless measurements, to assess the reliability of non-individualized baseline approaches. Additionally, for calibration-based algorithms, scatter plots showing cuffless BP minus calibration BP versus reference BP minus calibration BP are valuable. Figure 9e illustrates the correlation strength between cuffless BP and reference BP, showing the overall agreement between these two measurement approaches. Figure 9f highlights BP prediction based solely on calibration BP without using cuffless hemodynamic measurements, serving as a baseline comparison. Together, these visualizations offer insights into the performance and reliability of cuffless BP estimation compared to baseline models and population averages. Including scatter plots that integrate baseline models is also suggested, with all plots reporting the correlation coefficient for a more comprehensive analysis^149^.

While Fig. 9 defines the BP-Card as a generic reporting template, Fig. 10 illustrates how the framework can be operationalised in practice based on the work of Podder et al.^178^. Figure 10 illustrates the Model Card for calibrated algorithms: clinical purpose and use context Fig. 10(Module 0), dataset and cohort characteristics, including pre- and post-cleaning BP distributions Fig. 10(Module 1), and validation strategy with baseline model specification Fig. 10(Module 2), and condition-stratified calibration-based performance reporting (Module 3B). The BP-Card is designed to standardize the reporting of methods, metrics, cohort characteristics, and evaluation conditions; by doing so, it also enables structured comparison across studies and helps determine which approach is most appropriate for a given clinical context. The worked example makes this concrete. Figure 10 Module 1 requires reporting of cohort characteristics and BP distributions before and after data cleaning, showing that 20 participants were excluded during quality checks and that the post-cleaning cohort shifted toward lower mean SBP (122.7 vs. 128.1 mmHg) and a lower proportion of stage 2 hypertension (10.0% vs. 18.4%); without this transparency, performance comparisons across studies may reflect favorable data selection rather than genuine algorithmic differences. Module 2 mandates baseline-comparator reporting across input configurations, revealing a clear performance ranking: PPG alone (MAD 11.24 ± 10.53 mmHg), ECG alone (MAD 10.45 ± 10.70 mmHg), PPG + ECG (MAD 9.67 ± 9.52 mmHg), and PPG + ECG + Demographics (MAD 9.05 ± 9.02 mmHg). Without this systematic comparison, it would not be possible to quantify the incremental gain of each added modality—information that is essential when selecting among competing approaches for a given application. Module 3B requires results to be reported separately by testing condition, showing that accuracy is lower under dynamic ambulatory conditions (MAD 8.61 ± 8.28 mmHg, CP5 44.92%) than under static in-clinic settings (MAD 8.08 ± 8.43 mmHg, CP5 50.55%); this disaggregation ensures that a device’s suitability can be judged for the specific operating context in which it will be deployed, rather than based on a single pooled metric. Together, these observations illustrate that clinically relevant performance differences, modality-specific contributions, and dataset shifts are not apparent from headline summary metrics alone, and instead become visible only when structured, standardized disclosure is applied—enabling the community to compare methods on equal terms and identify which is best suited for a given clinical use case.

Beyond methodological heterogeneity, our supplementary model-card analysis further revealed substantial deficiencies in reporting transparency, even among studies that theoretically fulfilled current IEEE and/or AAMI validation criteria. Among the 86 reviewed studies, only 19 reported performance levels that theoretically satisfied at least one of the IEEE and/or AAMI validation standards. After excluding studies with fewer than 85 participants, only 12 publications remained (7 calibration-free^94,103,129,144–146,179^ and 5 calibration-based^95,106,121,127,147^). The corresponding model cards are provided in the Supplementary Material. Key dataset characteristics are frequently underreported particularly after data preprocessing, including participant count, total paired BP samples and BP distributions. As a result, the true predictive difficulty of the task and the real robustness of the proposed models often remain unclear. This issue is particularly critical because BP variability differs substantially across datasets, meaning that apparently favorable error metrics may simply reflect narrow BP distributions rather than strong predictive capability. Among the five calibration-based studies, only two reported subject-specific ΔBP distributions. Importantly, these studies showed markedly different variability ranges, with reported SBP/DBP ΔBP values of approximately 26/18 mmHg in one case versus 34/21 mmHg in another, illustrating how models evaluated under broader BP fluctuations likely face a substantially more challenging prediction task. However, such variability information is rarely disclosed, limiting meaningful cross-study comparison. Similarly, only one calibration-based study evaluated performance under dynamic conditions and only one additional study assessed model stability over time after calibration, despite both dynamic and long-term testing being essential for assessing robustness under realistic BP fluctuations and prolonged operation. Likewise, in calibration-free approaches, no study reported population-based BP distributions, and only one stratified performance across BP profiles. Notably, none of the 86 reviewed studies reported baseline model results. We only found one study^71^ that explicitly incorporated baseline model comparisons, demonstrating that several hemodynamic models, particularly PAT-based approaches, did not provide statistically significant improvements over calibration-only baseline predictions. These findings further reinforce the importance of baseline models for contextualizing performance and distinguishing genuine physiological signal contribution from population priors or demographic information. The corresponding model card is also provided in the Supplementary Material.

Taken together, these findings indicate that current limitations are not restricted to methodological heterogeneity, but also involve insufficient transparency in dataset characterization and evaluation reporting. Without standardized disclosure, it remains difficult to determine whether reported performances reflect clinically meaningful predictive capability or favorable dataset characteristics. This lack of transparency also hinders fair comparison across studies and slows progress toward robust cuffless BP systems suitable for real-world deployment. Finally, it is noteworthy that nearly all reviewed studies were conducted under in-clinic or controlled conditions, while truly ambulatory evaluations remain scarce. This represents a major translational gap, as the primary motivation for cuffless BP monitoring is precisely its potential for continuous real-world and ambulatory assessment, where conventional cuff-based devices remain impractical despite their superior accuracy in controlled settings. The importance of this limitation has already been demonstrated in previous work^93^, where statistically significant performance degradation was observed between in-clinic and ambulatory measurements, even when evaluated on the same subjects, further emphasizing the difficulty of robust real-world BP estimation. The model card for this study is also included in the Supplementary Material.

Our recommendations below focus on reducing this fragility through dataset breadth, subject-wise evaluation, baselines, and transparent reporting. Before discussing fairness-relevant findings, we distinguish between two related but distinct activities. A formal fairness audit evaluates whether model performance differs systematically across demographic or clinical subgroups and therefore requires subgroup-stratified error metrics, such as *MAD* or *ME* by skin tone, sex, age, BP stratum, comorbidity status, or medication use. A demographic and subgroup reporting-gap analysis, by contrast, evaluates whether the primary literature reports the cohort descriptors and subgroup-level performance information required to conduct such an audit. Because subgroup-stratified performance metrics were reported in only a small minority of the 86 reviewed studies, the prerequisites for a quantitative fairness audit are not met in the current literature. The analysis reported below should therefore be interpreted as a reporting-gap analysis: it characterizes the field’s readiness to support fairness evaluation rather than measuring algorithmic disparities directly. This distinction is important because the absence of subgroup-level evidence is itself a methodological limitation and directly motivates the fairness-relevant fields of the BP-Card.

Bias, fairness-relevant reporting, and equity are central to the clinical viability of cuffless BP estimation. Optical and wearable signals are affected by skin tone, contact pressure, adiposity, motion, and ambient light, which can alter signal-to-noise and downstream accuracy. Several systematic risk factors emerge from the reviewed literature: (i) Skewed datasets (ICU/healthy vs. ambulatory hypertensive) can bias models toward normotension. (ii) Skin-dependent light absorption and sensor contact force alter PPG morphology. (iii) Sex, age, and arterial stiffness modify PWV relationships. (iv) Antihypertensive medications (e.g., beta-blockers, diuretics) influence autonomic and vascular tone. From the 86 studies reviewed, only one reported skin tone without using the Fitzpatrick scale^106^ and two documented ethnicity^106,120^. Performance was stratified by sex in three papers^103,106,120^, by age in two^49,103^, and by hypertension stage in five^49,93^. Additionally, only one study vaguely reported antihypertensive medication status^49^. To mitigate these sources of bias and to enable future fairness audits, we recommend that future studies explicitly document cohort characteristics relevant to fairness and equity, including skin tone (e.g., Fitzpatrick I–VI), sex, age, BMI/arm circumference, ethnicity/region, contact pressure or strap tightness, sensor location, ambient light, activity state, and antihypertensive medication class, dose, and regimen. Model evaluation should move beyond aggregated performance and report subject-wise, stratified results across BP categories (following IEEE 1708a recommendations), using standardized metrics (*MD* ± *SD*, *MAD*, *CP*_5_, *CP*_10_, *CP*_15_, *MAPD*, and *R*). Priority should also be given to public benchmarks with broader diversity (e.g., PulseDB, MIMIC-BP, Aurora-BP), with transparent reporting of inclusion and exclusion criteria and their impact on fairness-relevant evaluation. In this context, multimodal sensing strategies have shown promise: combining multiple PPG modalities has been demonstrated to improve hypertension detection, particularly in populations where single-site PPG is unreliable, and may help mitigate bias arising from skin tone, contact pressure, or motion artifacts^180^.

Out-of-office BP assessment improves cardiovascular risk stratification beyond clinic readings, particularly among those whose in-office BP levels are assumed to carry no or tiny increased cardiovascular risk^9,10^. Reflecting this evidence, the 2017 ACC/AHA and the 2018 ESC/ESH guidelines recommend incorporating home or ambulatory BP monitoring to confirm HTN and to guide titration of therapy, including in conjunction with telehealth support^7,8^. Cuffless monitoring can extend these benefits by reducing discomfort, sleep disturbance, and adherence barriers associated with repeated cuff inflations. These advantages are especially relevant for longitudinal monitoring, nocturnal profiling, and pediatric settings where conventional devices are poorly tolerated. By enabling frequent, passive measurements, cuffless wearables offer a practical route to earlier detection, tighter control, and more personalized management of HTN.

Realizing this clinical potential, however, requires methodological rigor that ensures reproducible and clinically trustworthy results. Prior work indicates that features derived from PPG track BP dynamics, underscoring the need for systematic and transparent reporting of feature sets and preprocessing choices^176^. Consensus guidance further highlights consistent fiducial point selection and robust validation strategies, while complementary evidence shows that multimodal approaches can improve accuracy when single-site PPG is unreliable^180^ and ECG features add robustness and interpretability for HTN assessment^181^. Together, these findings motivate the need for a unified signal-domain consensus that can be embedded within broader evaluation frameworks.

At the same time, evidence standards must align with the intended clinical use: beyond subject-independent internal validation, studies should include external testing across diverse BP ranges and populations, explicitly sampling older adults, individuals with increased arterial stiffness, and patients with multimorbidity, groups that remain underrepresented in current validations. Protocols should induce dynamic BP changes (e.g., posture, activity, or medication timing) and report performance by BP stratum. Ultimately, randomized controlled trials powered for clinical outcomes are needed to determine whether cuffless monitoring improves awareness, increases the likelihood of achieving guideline-recommended BP targets, and reduces adverse cardiovascular events compared with current standard care. In summary, cuffless BP monitoring is well positioned to complement guideline-endorsed out-of-office assessment, but translation into routine clinical practice depends on the adoption of robust, standardized evaluation frameworks and population-aware validation strategies. Community benchmarks that adopt these practices can accelerate safe clinical integration while ensuring benefits are realized equitably across heterogeneous patient groups.

In this review, we identify limited generalizability across populations and real-world conditions as a central limitation of current cuffless BP estimation methods. Our analysis shows that inconsistent cohort characterization, heterogeneous performance reporting, and insufficient validation protocols remain major barriers to clinical translation. In particular, the lack of standardized reporting regarding BP distributions, calibration procedures, baseline comparisons, and validation under realistic ambulatory conditions makes it difficult to determine the true robustness and predictive capability of many published models. Based on current evidence, we distinguish the two dominant paradigms in BP estimation (calibration-free and calibration-based approaches) and clarify their distinct methodological requirements in terms of population diversity, validation strategy, and performance reporting. Beyond summarizing existing approaches, this review highlights critical methodological limitations that are frequently overlooked, including the absence of baseline-model evaluation, insufficient characterization of BP variability, and the limited use of ambulatory validation protocols. Building on this synthesis, we consolidate current knowledge into a structured set of practical recommendations for dataset characterization, evaluation design, and performance reporting through the proposed BP-card reporting framework. We believe that the adoption of harmonized validation standards and transparent reporting practices is essential to improve reproducibility, enable fair comparison across studies, and accelerate the development of clinically meaningful cuffless BP monitoring technologies suitable for continuous real-world and ambulatory use.

## Methods

### Systematic literature search and study selection

A systematic review on noninvasive, continuous cuffless BP estimation methods was conducted, following the Preferred Reporting Items for Systematic Reviews and Meta-Analyses (PRISMA) guidelines. Systematic searches were conducted in PubMed (MEDLINE) and IEEExplore. The inclusion criteria consisted of English, full-text articles published in peer-reviewed journals between January 2017 and March 2025, focusing on noninvasive, continuous cuffless BP estimation methods. Only original research articles were included, while review and perspective pieces were excluded. Studies were also excluded if they lacked methodological details on data collection or BP estimation algorithms, such as those focused primarily on electronic design or signal processing techniques. Two reviewers screened the records to determine whether a study met the inclusion/exclusion criteria based on its title and abstract. Subsequently, two independent reviewers screened the records based on the full text. The search terms used included: *continuous*, *cuffless*, *photoplethysmogram*, *blood pressure*, *estimation*, *algorithm*, and *validation*. Detailed database-specific search commands are provided in Supplementary Note 2.

**Fig. 11:**
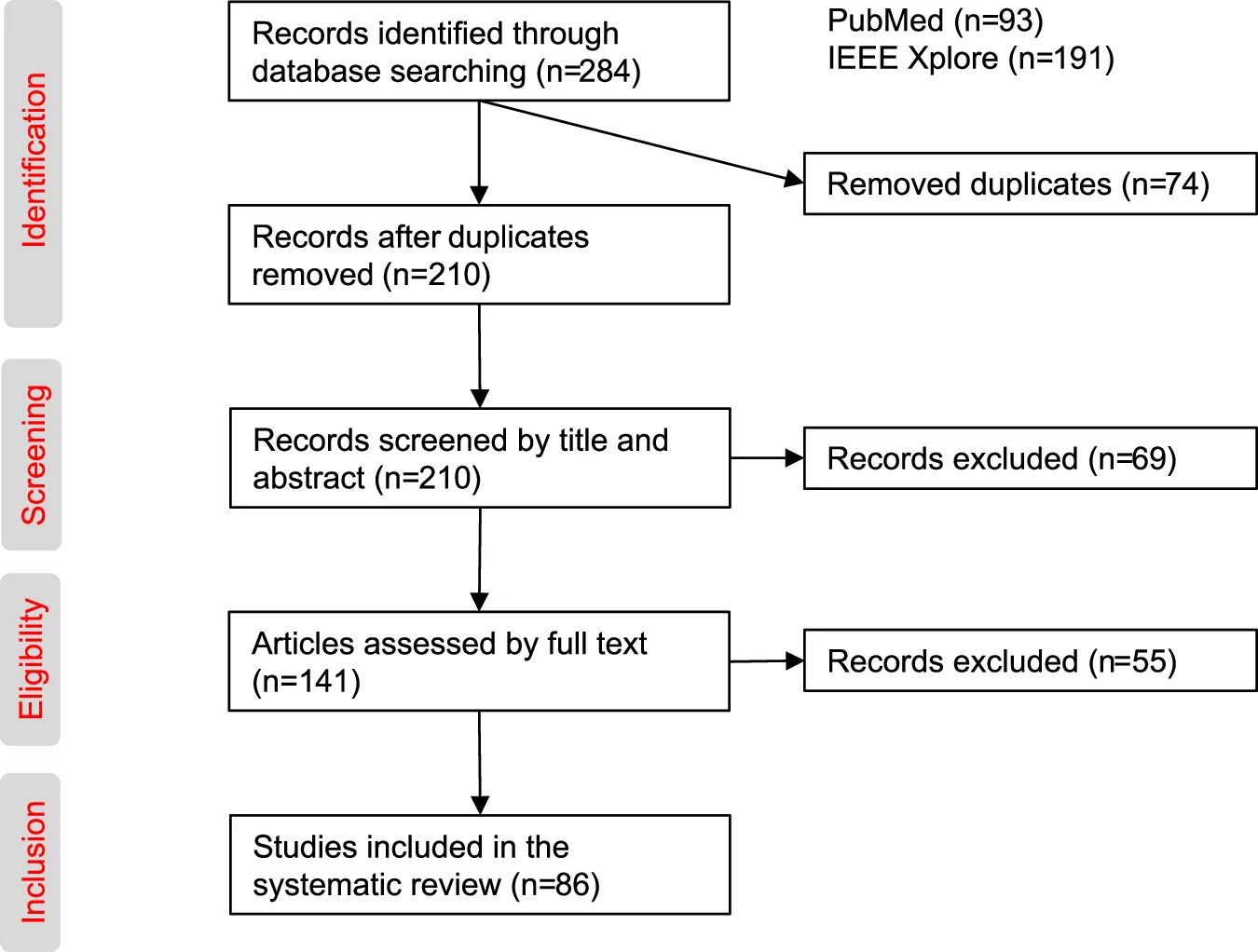
Systematic review process. Study flow diagram for the identification of papers following PRISMA guidelines.

A total of 284 records were identified through database searches, of which 74 were duplicates. After title and abstract screening, 69 records were excluded. Full-text assessment led to the removal of 55 additional articles, resulting in 86 studies being included in the final systematic review (Fig. 11).

### Meta-analysis

We quantitatively pooled SBP mean error (*ME*; positive = overestimation) using random-effects meta-analysis. After applying the inclusion criteria (sample size reporting and non-subject-specific reporting) and excluding entries with missing mean or SE, the final pooled set comprised *k* = 46 effects. For each effect, we recorded the validation protocol (subject-wise, record-wise, LOOCV, etc.), sensor modality, and reference device.

Although the included studies vary in sensor modality, dataset, reference device, calibration protocol, and validation design, all studies estimate the same underlying construct: the signed bias (ME) of a cuffless BP estimator relative to a reference measurement. Pooling is therefore conceptually justified. We used a random-effects model, which does not assume a single common true effect but instead treats each study as estimating a draw from a distribution of true effects, with the variance of that distribution (*τ*^2^) estimated from the data. Under this framework, between-study heterogeneity is a quantity to be estimated and interpreted, not a violation of an assumption. The high *I*^2^ values observed throughout are accordingly reported as a substantive finding about the consistency of the literature, not as a pooling artifact. Pooled effects were estimated using DerSimonian–Laird (DL) random-effects (RE) models for the overall set and for a priori validation subgroups (subject-wise vs. record-wise). Sensitivity to extreme values was examined via (i) trimming (∣ME∣ ≤ 5 mmHg) and (ii) Winsorizing at 5 mmHg. To assess the impact of between-study variance, we also report pooled effects obtained using restricted maximum likelihood (REML) with Hartung–Knapp (HK) small-sample adjustment (REML+HK), which provides more conservative confidence intervals under high heterogeneity. Small-study and selective-reporting biases were examined using Egger’s regression test, the precision-effect test (PET), and the precision-effect estimate with standard error (PEESE), each estimated with heteroscedasticity-consistent HC3 covariance.

### Risk-of-bias assessment

We developed a five-domain framework adapted from QUADAS-2^155^ and PROBAST^156^, using the Low/Unclear/High rating scale. The five domains are: (i) *Sample size*: number of independent participants; (ii) *BP spectrum reporting*: whether the study reports the BP distribution or range of the test cohort; (iii) *Reference BP standard*:invasive arterial or documented validated cuff measurement (Low); undocumented cuff measurement (Unclear); self-reported or unvalidated measurement (High); (iv) *Calibration/evaluation clarity*: whether the calibration procedure and evaluation protocol are described in sufficient detail to be reproducible; and (v) *Validation independence*: fully independent external test cohort (Low); subject-wise internal validation (Unclear); record-, window-, or beat-wise splits, or splits where participant-level separation cannot be confirmed (High). No study in our sample used a fully independent external test cohort, so no study was eligible for Low risk in this domain. The overall study-level rating used the worst-domain rule, following QUADAS-2 convention. Domains (i)–(iv) were rated from explicit metadata fields extracted during data abstraction; domain (v) was rated from the reported validation procedure. The resulting domain-level and overall RoB distributions are reported in Fig. 8.

Fairness-related extraction was limited to the availability of demographic descriptors, clinical subgroup variables, and subgroup-stratified performance reporting. Because subgroup-level performance data were sparse and inconsistently reported, this component was treated as a demographic and subgroup reporting-gap analysis rather than a formal fairness audit.

All acronyms used in the manuscript are defined at first occurrence and consolidated in Supplementary Table 1 for ease of reference.

## Supplementary information

Supplementary information

## Data Availability

Data sharing is not applicable to this article as no datasets were generated or analyzed during the current study. This review article is based on previously published studies and does not contain original research data. All data supporting the findings of this study are available within the article and its references. The study did not involve human participants, and no informed consent was required.

## References

[1] Roth, G. A. et al. Global burden of cardiovascular diseases and risk factors, 1990–2019. *J. Am. Coll. Cardiol.* **76**, 2982–3021 (2020). 10.1016/j.jacc.2020.11.010 PMC7755038 33309175

[2] Beaney, T. et al. May measurement month 2019: the global blood pressure screening campaign of the international society of hypertension. *Hypertension* **76**, 333–341 (2020). 10.1161/HYPERTENSIONAHA.120.14874 32419505

[3] Singh, M. et al. White coat effect: Is it because of the hospital setting, or is it physician-induced? *Cureus* **15**, 1–8 (2023). 10.7759/cureus.38144 PMC10214880 37257162

[4] Yang, W.-Y. et al. Association of office and ambulatory blood pressure with mortality and cardiovascular outcomes. *JAMA* **322**, 409–420 (2019). 10.1001/jama.2019.9811 PMC6822661 31386134

[5] Asayama, K. et al. Cardiovascular outcomes in the first trial of antihypertensive therapy guided by self-measured home blood pressure. *Hypertens. Res.* **35**, 1102–1110 (2012). 10.1038/hr.2012.125 22895063

[6] Ohkubo, T. et al. Home blood pressure measurement has a stronger predictive power for mortality than does screening blood pressure measurement: a population-based observation in ohasama, japan. *J. Hypertens.* **16**, https://journals.lww.com/jhypertension/fulltext/1998/16070/home_blood_pressure_measurement_has_a_stronger.10.aspx (1998). 10.1097/00004872-199816070-00010 9794737

[7] Williams, B. et al. 2018 ESC/ESH guidelines for the management of arterial hypertension. *Eur. Heart J.* **39**, 3021–3104 (2018). 10.1093/eurheartj/ehy339 30165516

[8] Whelton, P. K. et al. 2017 ACC/AHA/AAPA/ABC/ACPM/AGS/APhA/ASH/ASPC/NMA/PCNA guideline for the prevention, detection, evaluation, and management of high blood pressure in adults: a report of the American College of Cardiology/American Heart Association Task Force on clinical practice guidelines. *Hypertension* **71**, e13–e115 (2018). 10.1161/HYP.0000000000000065 29133356

[9] Asayama, K. et al. Risk stratification by self-measured home blood pressure across categories of conventional blood pressure: a participant-level meta-analysis. *PLOS Med.* **11**, 1–16 (2014). 10.1371/journal.pmed.1001591 PMC3897370 24465187

[10] Brguljan-Hitij, J. et al. Risk stratification by ambulatory blood pressure monitoring across JNC classes of conventional blood pressure. *Am. J. Hypertens.* **27**, 956–965 (2014). 10.1093/ajh/hpu002 24572704

[11] Elgendi, M. et al. The use of photoplethysmography for assessing hypertension. *NPJ Digit. Med.* **2**, 60 (2019). 10.1038/s41746-019-0136-7 PMC6594942 31388564

[12] Falter, M. et al. Smartwatch-based blood pressure measurement demonstrates insufficient accuracy. *Front. Cardiovasc. Med.* **9**, 958212 (2022). 10.3389/fcvm.2022.958212 PMC9309348 35898281

[13] Lee, H. Y. et al. Smartphone/smartwatch-based cuffless blood pressure measurement : a position paper from the Korean Society of Hypertension. *Clin. Hypertens.* **27**, 4 (2021). 10.1186/s40885-020-00158-8 PMC7831256 33494809

[14] Stergiou, G. S., Alpert, B. S., Mieke, S., Wang, J. & O’Brien, E. Validation protocols for blood pressure measuring devices in the 21st century. *J. Clin. Hypertens.* **20**, 1096–1099 (2018). 10.1111/jch.13294 PMC8030852 30003697

[15] Stergiou, G. S. et al. A universal standard for the validation of blood pressure measuring devices: association for the Advancement of Medical Instrumentation/European Society of Hypertension/International Organization for Standardization (AAMI/ESH/ISO) Collaboration Statement. *J. Hypertens.* **36**, 472–478 (2018). 10.1097/HJH.0000000000001634 PMC5796427 29384983

[16] Schutte, A. E., Kollias, A. & Stergiou, G. S. Blood pressure and its variability: classic and novel measurement techniques. *Nat. Rev. Cardiol.* **19**, 643–654 (2022). 10.1038/s41569-022-00690-0 PMC9017082 35440738

[17] Stergiou, G. S. et al. 2021 European Society of Hypertension practice guidelines for office and out-of-office blood pressure measurement. *J. Hypertens.* **39**, 1293–1302 (2021). 10.1097/HJH.0000000000002843 33710173

[18] El-Hajj, C. & Kyriacou, P. A. A review of machine learning techniques in photoplethysmography for the non-invasive cuff-less measurement of blood pressure. *Biomed. Signal Process. Control* **58**, 101870 (2020).

[19] Koshimizu, H., Kojima, R. & Okuno, Y. Future possibilities for artificial intelligence in the practical management of hypertension. *Hypertens. Res.* **43**, 1327–1337 (2020). 10.1038/s41440-020-0498-x 32655135

[20] Martinez-Ríos, E., Montesinos, L., Alfaro-Ponce, M. & Pecchia, L. A review of machine learning in hypertension detection and blood pressure estimation based on clinical and physiological data. *Biomed. Signal Process. Control* **68**, 102813 (2021).

[21] Cheung, M. Y., Sabharwal, A., Cote, G. L. & Veeraraghavan, A. Wearable blood pressure monitoring devices: understanding heterogeneity in design and evaluation. *IEEE Trans. Biomed. Eng.* **71**, 3569–3592 (2024). 10.1109/TBME.2024.3434344 PMC11799359 39106139

[22] Park, J., Seok, H. S., Kim, S.-S. & Shin, H. Photoplethysmogram analysis and applications: an integrative review. *Front. physiol.* **12**, 808451 (2022). 10.3389/fphys.2021.808451 PMC8920970 35300400

[23] Hu, J.-R. et al. Validating cuffless continuous blood pressure monitoring devices. *Cardiovasc. Digit. Health J.* **4**, 9–20 (2023). 10.1016/j.cvdhj.2023.01.001 PMC9971997 36865583

[24] Charlton, P. H. et al. Wearable photoplethysmography for cardiovascular monitoring. *Proc. IEEE* **110**, 355–381 (2022). 10.1109/JPROC.2022.3149785 PMC7612541 35356509

[25] Dai, R., Lu, C., Avidan, M. & Kannampallil, T. Respwatch: robust measurement of respiratory rate on smartwatches with photoplethysmography. In *Proc. International Conference on Internet-of-Things Design and Implementation* 208–220 (2021).

[26] Wu, B.-J., Wu, B.-F. & Hsu, C.-P. Camera-based blood pressure estimation via Windkessel model and waveform features. *IEEE Trans. Instrum. Meas.* **72**, 1–13 (2023).

[27] Li, Y. et al. Hybrid D1DCnet using forehead iPPG for continuous and noncontact blood pressure measurement. *IEEE Sens. J.* **23**, 2727–2736 (2023).

[28] Kireev, D. et al. Continuous cuffless monitoring of arterial blood pressure via graphene bioimpedance tattoos. *Nat. Nanotechnol.* **17**, 864–870 (2022). 10.1038/s41565-022-01145-w 35725927

[29] Sato, T., Nishinaga, M., Kawamoto, A., Ozawa, T. & Takatsuji, H. Accuracy of a continuous blood pressure monitor based on arterial tonometry. *Hypertension* **21**, 866–874 (1993). 10.1161/01.hyp.21.6.866 8500866

[30] Cheng, H.-M., Lang, D., Tufanaru, C. & Pearson, A. Measurement accuracy of non-invasively obtained central blood pressure by applanation tonometry: a systematic review and meta-analysis. *Int. J. Cardiol.* **167**, 1867–1876 (2013). 10.1016/j.ijcard.2012.04.155 22622052

[31] Finnegan, E. et al. Features from the photoplethysmogram and the electrocardiogram for estimating changes in blood pressure. *Sci. Rep.* **13**, 986 (2023). 10.1038/s41598-022-27170-2 PMC9849280 36653426

[32] Guo, C.-Y., Huang, C.-H., Chang, C.-C., Wang, K.-J. & Hsieh, T.-L. Combining local PWV and quantified arterial changes for calibration-free cuffless blood pressure estimation: a clinical validation. *IEEE Sens. J.* **23**, 658–668 (2023).

[33] Zhang, B., Wei, Z., Ren, J., Cheng, Y. & Zheng, Z. An empirical study on predicting blood pressure using classification and regression trees. *IEEE Access* **6**, 21758–21768 (2018).

[34] Liu, Z.-D. et al. HGCTNet: handcrafted feature-guided CNN and transformer network for wearable cuffless blood pressure measurement. *IEEE J. Biomed. Health Inform.* **28**, 3882–3894 (2024). 10.1109/JBHI.2024.3395445 38687656

[35] Cen, Y. et al. OVAR-BPnet: a general pulse wave deep learning approach for cuffless blood pressure measurement. *IEEE J. Biomed. health Inform.* **28**, 5829–5841 (2024). 10.1109/JBHI.2024.3423461 38963748

[36] Johnson, A. E. W. et al. MIMIC-IV, a freely accessible electronic health record dataset. *Sci. Data* **10**, 1 (2023). 10.1038/s41597-022-01899-x PMC9810617 36596836

[37] Goldberger, A. L. et al. PhysioBank, PhysioToolkit, and PhysioNet: components of a new research resource for complex physiologic signals. *Circulation* **101**, https://www.ahajournals.org/doi/10.1161/01.CIR.101.23.e215 (2000). 10.1161/01.cir.101.23.e215 10851218

[38] Saeed, M., Lieu, C., Raber, G. & Mark, R. MIMIC II: a massive temporal ICU patient database to support research in intelligent patient monitoring. In *Computers in Cardiology* 641–644. http://ieeexplore.ieee.org/document/1166854/ (IEEE, 2002). 14686455

[39] Johnson, A. E. et al. MIMIC-III, a freely accessible critical care database. *Sci. Data* **3**, 160035 (2016). 10.1038/sdata.2016.35 PMC4878278 27219127

[40] Kachuee, M., Kiani, M. M., Mohammadzade, H. & Shabany, M. Cuffless blood pressure estimation algorithms for continuous health-care monitoring. *IEEE Trans. Biomed. Eng.* **64**, 859–869 (2017). 10.1109/TBME.2016.2580904 27323356

[41] Liang, Y., Chen, Z., Liu, G. & Elgendi, M. A new, short-recorded photoplethysmogram dataset for blood pressure monitoring in China. *Sci. Data* **5**, 180020 (2018). 10.1038/sdata.2018.20 PMC5827692 29485624

[42] Lee, H.-C. & Jung, C.-W. Vital Recorder—a free research tool for automatic recording of high-resolution time-synchronised physiological data from multiple anaesthesia devices. *Sci. Rep.* **8**, 1527 (2018). 10.1038/s41598-018-20062-4 PMC5784161 29367620

[43] Argha, A. *Auscultatory and Oscillometry Blood Pressure Data*. https://ieee-dataport.org/documents/auscultatory-and-oscillometry-blood-pressure-data (2020).

[44] Liu, D., Görges, M. & Jenkins, S. A. University of Queensland vital signs dataset: development of an accessible repository of anesthesia patient monitoring data for research. *Anesth. Analg.* **114**, 584–589 (2012). 10.1213/ANE.0b013e318241f7c0 22190558

[45] Sofia, G. SINTEC project (SHIMMER database). Accessed 11 March 2023. https://github.com/SOFIAGALICI/BP_Evaluation_Shimmer (2022).

[46] Wang, W., Mohseni, P., Kilgore, K. L. & Najafizadeh, L. PulseDB: a large, cleaned dataset based on MIMIC-III and VitalDB for benchmarking cuff-less blood pressure estimation methods. *Front. Digit. Health* **4**, 1090854 (2023). 10.3389/fdgth.2022.1090854 PMC9944565 36844249

[47] Sanches, I. et al. Mimic-bp: a curated dataset for blood pressure estimation. *Sci. Data* **11**, 1233 (2024). 10.1038/s41597-024-04041-1 PMC11568151 39548096

[48] Mieloszyk, R. et al. A comparison of wearable tonometry, photoplethysmography, and electrocardiography for cuffless measurement of blood pressure in an ambulatory setting. *IEEE J. Biomed. Health Inform.* **26**, 2864–2875 (2022). 10.1109/JBHI.2022.3153259 35201992

[49] Liu, Z.-D. et al. Cuffless blood pressure measurement using smartwatches: a large-scale validation study. *IEEE J. Biomed. Health Inform.* **27**, 4216–4227 (2023). 10.1109/JBHI.2023.3278168 37204948

[50] Chakraborty, A., Goswami, D., Mukhopadhyay, J. & Chakrabarti, S. Blood pressure estimation based on photopethysmography for personalized healthcare. *IEEE Trans. Consum. Electron*. 1–1. https://ieeexplore.ieee.org/document/10253681/ (2023).

[51] Qiu, S., Zhang, Y.-T., Lau, S.-K. & Zhao, N. Scenario adaptive cuffless blood pressure estimation by integrating cardiovascular coupling effects. *IEEE J. Biomed. Health Inform.* **27**, 1375–1385 (2023). 10.1109/JBHI.2022.3227235 37015611

[52] Khalid, S. G. et al. Cuffless blood pressure estimation using single channel photoplethysmography: a two-step method. *IEEE Access* **8**, 58146–58154 (2020).

[53] Maqsood, S., Xu, S., Springer, M. & Mohawesh, R. A benchmark study of machine learning for analysis of signal feature extraction techniques for blood pressure estimation using photoplethysmography (PPG). *IEEE Access* **9**, 138817–138833 (2021).

[54] Brophy, E., De Vos, M., Boylan, G. & Ward, T. Estimation of continuous blood pressure from PPG via a federated learning approach. *Sensors* **21**, 6311 (2021). 10.3390/s21186311 PMC8471262 34577518

[55] Figini, V. et al. Improving cuff-less continuous blood pressure estimation with linear regression analysis. *Electronics* **11**, 1442 (2022).

[56] Wong, M. K. F., Hei, H., Lim, S. Z. & Ng, E. Y. K. Applied machine learning for blood pressure estimation using a small, real-world electrocardiogram and photoplethysmogram dataset. *Math. Biosci. Eng.* **20**, 975–997 (2023). 10.3934/mbe.2023045 36650798

[57] Argüello-Prada, E. J. & Castaño Mosquera, C. D. Exploring supervised machine learning models to estimate blood pressure using non-fiducial features of the photoplethysmogram (PPG) and its derivatives. *Phys. Eng. Sci. Med.* **48**, 1399–1414 (2025). 10.1007/s13246-025-01593-3 PMC12511243 40760394

[58] Min, S. et al. Wearable blood pressure sensors for cardiovascular monitoring and machine learning algorithms for blood pressure estimation. *Nat. Rev. Cardiol.* **22**, 629–648 (2025). 10.1038/s41569-025-01127-0 PMC13200057 39966649

[59] Shao, J., Shi, P., Hu, S., Liu, Y. & Yu, H. An optimization study of estimating blood pressure models based on pulse arrival time for continuous monitoring. *J. Healthc. Eng.* **2020**, 1–12 (2020). 10.1155/2020/1078251 PMC7035551 32104555

[60] Vlachopoulos, C., O’Rourke, M. & Nichols, W. W. *McDonald’s Blood Flow in Arteries: Theoretical, Experimental and Clinical Principles* 0 edn. https://www.taylorfrancis.com/books/9781444128789 (CRC Press, 2011).

[61] Proença, J., Muehlsteff, J., Aubert, X. & Carvalho, P. Is pulse transit time a good indicator of blood pressure changes during short physical exercise in a young population? In *2010 Annual International Conference of the IEEE Engineering in Medicine and Biology* 598–601 (2010). 10.1109/IEMBS.2010.5626627 21096104

[62] Wong, M. Y.-M., Poon, C. C.-Y. & Zhang, Y.-T. An evaluation of the cuffless blood pressure estimation based on pulse transit time technique: a half year study on normotensive subjects. *Cardiovasc. Eng.* **9**, 32–38 (2009). 10.1007/s10558-009-9070-7 19381806

[63] Baek, H. J., Kim, K. K., Kim, J. S., Lee, B. & Park, K. S. Enhancing the estimation of blood pressure using pulse arrival time and two confounding factors. *Physiol. Meas.* **31**, 145–157 (2010). 10.1088/0967-3334/31/2/002 20009186

[64] Wibmer, T. et al. Pulse transit time and blood pressure during cardiopulmonary exercise tests. *Physiol. Res*. 287–296. https://www.biomed.cas.cz/physiolres/pdf/63/63_287.pdf (2014). 10.33549/physiolres.932581 24564606

[65] Choi, Y., Zhang, Q. & Ko, S. Noninvasive cuffless blood pressure estimation using pulse transit time and Hilbert–Huang transform. *Comput. Electr. Eng.* **39**, 103–111 (2013).

[66] Ma, H. T. A blood pressure monitoring method for stroke management. *Biomed. Res. Int.* **2014**, 1–7 (2014). 10.1155/2014/571623 PMC4150505 25197651

[67] Masè, M., Mattei, W., Cucino, R., Faes, L. & Nollo, G. Feasibility of cuff-free measurement of systolic and diastolic arterial blood pressure. *J. Electrocardiol.* **44**, 201–207 (2011). 10.1016/j.jelectrocard.2010.11.019 21353067

[68] Fung, P., Dumont, G., Ries, C., Mott, C. & Ansermino, M. Continuous noninvasive blood pressure measurement by pulse transit time. In *The 26th Annual International Conference of the IEEE Engineering in Medicine and Biology Society* Vol. 3, 738–741 http://ieeexplore.ieee.org/document/1403264/ (IEEE, San Francisco, CA, USA, 2004). 10.1109/IEMBS.2004.1403264 17271783

[69] Wang, T.-W., Chen, W.-X., Chu, H.-W. & Lin, S.-F. Single-channel bioimpedance measurement for wearable continuous blood pressure monitoring. *IEEE Trans. Instrum. Meas.* **70**, 1–9 (2021).

[70] Polinski, A. et al. A meta-analysis of pulse arrival time based blood pressure estimation. In *The Annual International Conference of the IEEE Engineering in Medicine and Biology Society* 5822–5825 https://ieeexplore.ieee.org/document/8513605/ (Honolulu, HI, 2018). 10.1109/EMBC.2018.8513605 30441659

[71] Cisnal, A., Páez-Granados, D., Pérez-Turiel, J. & Fraile, J. C. Are pat-based models suitable for continuous blood pressure monitoring in ambulatory settings? In *2025 IEEE 38th International Symposium on Computer-Based Medical Systems (CBMS)*, 1–6 (2025).

[72] Esmaili, A., Kachuee, M. & Shabany, M. Nonlinear cuffless blood pressure estimation of healthy subjects using pulse transit time and arrival time. *IEEE Trans. Instrum. Meas.* **66**, 3299–3308 (2017).

[73] Hemon, M. C. & Phillips, J. P. Comparison of foot finding methods for deriving instantaneous pulse rates from photoplethysmographic signals. *J. Clin. Monit. Comput.* **30**, 157–168 (2016). 10.1007/s10877-015-9695-6 25902897

[74] Hafifah, A. H. & Nazrul Anuar, N. Methods of extracting feature from photoplethysmogram waveform for non-invasive diagnostic applications. *Int. J. Online Biomed. Eng.* **16**, 39 (2020).

[75] Sukor, J. A., Redmond, S. J. & Lovell, N. H. Signal quality measures for pulse oximetry through waveform morphology analysis. *Physiol. Meas.* **32**, 369–384 (2011). 10.1088/0967-3334/32/3/008 21330696

[76] Fischer, C., Domer, B., Wibmer, T. & Penzel, T. An algorithm for real-time pulse waveform segmentation and artifact detection in photoplethysmograms. *IEEE J. Biomed. Health Inform.* **21**, 372–381 (2017). 10.1109/JBHI.2016.2518202 26780821

[77] Zhang, Q., Zeng, X., Hu, W. & Zhou, D. A machine learning-empowered system for long-term motion-tolerant wearable monitoring of blood pressure and heart rate with ear-ECG/PPG. *IEEE Access* **5**, 10547–10561 (2017).

[78] Yoon, Y.-Z. et al. Cuff-less blood pressure estimation using pulse waveform analysis and pulse arrival time. *IEEE J. Biomed. Health Inform.* **22**, 1068–1074 (2018). 10.1109/JBHI.2017.2714674 28613189

[79] Ding, X. et al. Pulse transit time based continuous cuffless blood pressure estimation: a new extension and a comprehensive evaluation. *Sci. Rep.* **7**, 11554 (2017). 10.1038/s41598-017-11507-3 PMC5599606 28912525

[80] Byfield, R., Miller, M., Miles, J., Guidoboni, G. & Lin, J. Towards robust blood pressure estimation from pulse wave velocity measured by photoplethysmography sensors. *IEEE Sens. J.* **22**, 2475–2483 (2022).

[81] Rachim, V. P., Kang, S., Baek, J.-H. & Park, S.-M. Unobtrusive, cuffless blood pressure monitoring using a soft polymer sensor array with flexible hybrid electronics. *IEEE Sens. J.* **21**, 10132–10142 (2021).

[82] Nabeel, P., Karthik, S., Joseph, J. & Sivaprakasam, M. Arterial blood pressure estimation from local pulse wave velocity using dual-element photoplethysmograph probe. *IEEE Trans. Instrum. Meas.* **67**, 1399–1408 (2018).

[83] Guo, C.-Y., Chang, C.-C., Wang, K.-J. & Hsieh, T.-L. Assessment of a calibration-free method of cuffless blood pressure measurement: a pilot study. *IEEE J. Transl. Eng. Health Med.* **11**, 318–329 (2023). 10.1109/JTEHM.2022.3209754 PMC10756135 38163041

[84] Xing, X., Huang, R., Hao, L., Jiang, C. & Dong, W.-F. Temporal complexity in photoplethysmography and its influence on blood pressure. *Front. Physiol.* **14**, 1187561 (2023). 10.3389/fphys.2023.1187561 PMC10513039 37745247

[85] Mousavi, A., Inan, O. T., Mukkamala, R. & Hahn, J.-O. A physical model-based approach to one-point calibration of pulse transit time to blood pressure. *IEEE Trans. Biomed. Eng.* **71**, 477–483 (2024). 10.1109/TBME.2023.3307658 PMC10838522 37610893

[86] Khan Mamun, M. M. R. & Alouani, A. T. Cuffless blood pressure measurement using linear and nonlinear optimized feature selection. *Diagnostics* **12**, 408 (2022). 10.3390/diagnostics12020408 PMC8870879 35204499

[87] Ji, J., Dong, M., Lin, Q. & Tan, K. C. Noninvasive cuffless blood pressure estimation with dendritic neural regression. *IEEE Trans. Cybern.* **53**, 4162–4174 (2023). 10.1109/TCYB.2022.3141380 35113792

[88] Yen, C.-T., Chang, S.-N. & Liao, C.-H. Estimation of beat-by-beat blood pressure and heart rate from ECG and PPG using a fine-tuned deep CNN model. *IEEE Access* **10**, 85459–85469 (2022).

[89] Zhang, Y., Ren, X., Liang, X., Ye, X. & Zhou, C. A refined blood pressure estimation model based on single channel photoplethysmography. *IEEE J. Biomed. Health Inform.* **26**, 5907–5917 (2022). 10.1109/JBHI.2022.3206477 36103444

[90] Guo, P. et al. Cuffless blood pressure estimation model based on prior information of physiological data and LSTM. *IEEE Sens. J.* **24**, 34843–34850 (2024).

[91] Miao, F. et al. A novel continuous blood pressure estimation approach based on data mining techniques. *IEEE J. Biomed. Health Inform.* **21**, 1730–1740 (2017). 10.1109/JBHI.2017.2691715 28463207

[92] Wang, W., Mohseni, P., Kilgore, K. L. & Najafizadeh, L. *δ* bp-net: Monitoring “changes” in blood pressure using PPG with self-contrastive masking. *IEEE J. Biomed. Health Inform.* **28**, 7103–7115 (2024). 10.1109/JBHI.2024.3422023 PMC11969577 38954566

[93] Cisnal, A. et al. Robust feature selection for BP estimation in multiple populations: towards cuffless ambulatory BP monitoring. *IEEE J. Biomed. Health Inform.* **28**, 5768–5779 (2024). 10.1109/JBHI.2024.3411693 38857137

[94] Ma, C. et al. SMART-BP: SEM-ResNet and auto-regressor based on a two-stage framework for noninvasive blood pressure measurement. *IEEE Trans. Instrum. Meas.* **73**, 1–18 (2024).

[95] Baek, J.-H. et al. Cuffless blood pressure estimation model using clustering techniques. *IEEE Sens. J.* **24**, 32444–32454 (2024).

[96] Abdullah, S. & Kristoffersson, A. Machine learning approaches for cardiovascular hypertension stage estimation using photoplethysmography and clinical features. *Front. Cardiovasc. Med.* **10**, 1285066 (2023). 10.3389/fcvm.2023.1285066 PMC10725938 38111893

[97] Nishan, A. et al. A continuous cuffless blood pressure measurement from optimal PPG characteristic features using machine learning algorithms. *Heliyon* **10**, e27779 (2024). 10.1016/j.heliyon.2024.e27779 PMC10963242 38533045

[98] El-Hajj, C. & Kyriacou, P. Cuffless blood pressure estimation from PPG signals and its derivatives using deep learning models. *Biomed. Signal Process. Control* **70**, 102984 (2021).

[99] Li, P. & Laleg-Kirati, T.-M. Central blood pressure estimation from distal PPG measurement using semiclassical signal analysis features. *IEEE Access* **9**, 44963–44973 (2021).

[100] Sarkar, S. & Ghosh, A. Schrödinger spectrum based continuous cuff-less blood pressure estimation using clinically relevant features from PPG signal and its second derivative. *Comput. Biol. Med.* **166**, 107558 (2023). 10.1016/j.compbiomed.2023.107558 37806054

[101] Wang, W., Mohseni, P., Kilgore, K. L. & Najafizadeh, L. Cuff-less blood pressure estimation from photoplethysmography via visibility graph and transfer learning. *IEEE J. Biomed. Health Inform.* **26**, 2075–2085 (2022). 10.1109/JBHI.2021.3128383 34784289

[102] Rong, M. & Li, K. A multi-type features fusion neural network for blood pressure prediction based on photoplethysmography. *Biomed. Signal Process. Control.* **68**, 102772 (2021).

[103] Jamil, Z., Ting Lui, L. & Chan, R. H. M. Blood pressure estimation using self-attention mechanism built-In ResUNet on PulseDB: demographic fairness and generalization. *IEEE Sens. J.* **25**, 1694–1705 (2025).

[104] Chen, J. et al. rU-net, multi-scale feature fusion and transfer learning: unlocking the potential of cuffless blood pressure monitoring with PPG and ECG. *IEEE J. Biomed. Health Inform.* **29**, 166–176 (2025). 10.1109/JBHI.2024.3483301 39423074

[105] Liu, L., Zhang, Y.-T., Wang, W., Chen, Y. & Ding, X. Causal inference based cuffless blood pressure estimation: a pilot study. *Comput. Biol. Med.* **159**, 106900 (2023). 10.1016/j.compbiomed.2023.106900 37087777

[106] Qiao, M. et al. A two-branch framework for blood pressure estimation using photoplethysmography signals with deep learning and clinical prior physiological knowledge. *Physiol. Meas*. **46**, 025004 (2025). 10.1088/1361-6579/adae50 39854841

[107] Han, X. et al. Noncontact blood pressure estimation using bp-related cardiovascular knowledge: an uncalibrated method based on consumer-level camera. *IEEE Trans. Instrum. Meas.* **72**, 1–13 (2023).

[108] Wu, B.-F., Chiu, L.-W., Wu, Y.-C., Lai, C.-C. & Chu, P.-H. Contactless blood pressure measurement via remote photoplethysmography with synthetic data generation using generative adversarial network. In *2022 IEEE/CVF Conference on Computer Vision and Pattern Recognition Workshops (CVPRW)* 2129–2137 https://ieeexplore.ieee.org/document/9857123/ (IEEE, New Orleans, LA, USA, 2022). 10.1109/JBHI.2023.3265857 37037253

[109] De Marchi, B., Frigerio, M., De Nadai, S., Longinotti-Buitoni, G. & Aliverti, A. Blood Pressure continuous measurement through a wearable device: development and validation of a cuffless method. *Sensors* **21**, 7334 (2021). 10.3390/s21217334 PMC8588523 34770641

[110] Aguet, C. et al. Blood pressure monitoring during anesthesia induction using PPG morphology features and machine learning. *PLoS ONE* **18**, e0279419 (2023). 10.1371/journal.pone.0279419 PMC9897516 36735652

[111] Shoeibi, F., Najafiaghdam, E. & Ebrahimi, A. Nonlinear features of photoplethysmography signals for non-invasive blood pressure estimation. *Biomed. Signal Process. Control* **85**, 105067 (2023).

[112] Nour, M., Polat, K., Şentürk, Ü. & Arıcan, M. A novel cuffless blood pressure prediction: uncovering new features and new hybrid ML models. *Diagnostics* **13**, 1278 (2023). 10.3390/diagnostics13071278 PMC10093721 37046499

[113] Yang, S., Sohn, J., Lee, S., Lee, J. & Kim, H. C. Estimation and validation of arterial blood pressure using photoplethysmogram morphology features in conjunction with pulse arrival time in large open databases. *IEEE J. Biomed. Health Inform.* **25**, 1018–1030 (2021). 10.1109/JBHI.2020.3009658 32750963

[114] Gupta, S., Singh, A., Sharma, A. & Tripathy, R. K. Higher order derivative-based integrated model for cuff-less blood pressure estimation and stratification using PPG signals. *IEEE Sens. J.* **22**, 22030–22039 (2022).

[115] Simjanoska, M., Gjoreski, M., Gams, M. & Madevska Bogdanova, A. Non-invasive blood pressure estimation from ECG using machine learning techniques. *Sensors* **18**, 1160 (2018). 10.3390/s18041160 PMC5949031 29641430

[116] Liu, Z.-D. et al. Cuffless blood pressure estimation using pressure pulse wave signals. *Sensors* **18**, 4227 (2018). 10.3390/s18124227 PMC6308537 30513838

[117] Martinez, J., Passage, B., Mortazavi, B. J. & Jafari, R. Hypothesis scoring for confidence-aware blood pressure estimation with particle filters. *IEEE J. Biomed. Health Inform.* **27**, 4273–4284 (2023). 10.1109/JBHI.2023.3289192 PMC10567135 37363851

[118] Qiu, Z., Chen, D., Ling, B. W.-K., Liu, Q. & Li, W. Joint regression network and window function-based piecewise neural network for cuffless continuous blood pressure estimation only using single photoplethesmogram. *IEEE Trans. Consum. Electron.* **68**, 236–260 (2022).

[119] Yao, P. et al. Multi-dimensional feature combination method for continuous blood pressure measurement based on wrist PPG sensor. *IEEE J. Biomed. Health Inform.* **26**, 3708–3719 (2022). 10.1109/JBHI.2022.3167059 35417358

[120] Rao, A. et al. Clinical study of continuous non-invasive blood pressure monitoring in neonates. *Sensors* **23**, 3690 (2023). 10.3390/s23073690 PMC10098632 37050750

[121] Baek, S., Jang, J. & Yoon, S. End-to-end blood pressure prediction via fully convolutional networks. *IEEE Access* **7**, 185458–185468 (2019).

[122] Yen, C.-T., Liao, J.-X. & Huang, Y.-K. Applying a deep learning network in continuous physiological parameter estimation based on photoplethysmography sensor signals. *IEEE Sens. J.* **22**, 385–392 (2022).

[123] Mahardika T, N. Q., Fuadah, Y. N., Jeong, D. U. & Lim, K. M. PPG signals-based blood-pressure estimation using grid search in hyperparameter optimization of CNN–LSTM. *Diagnostics* **13**, 2566 (2023). 10.3390/diagnostics13152566 PMC10417316 37568929

[124] Leitner, J., Chiang, P.-H. & Dey, S. Personalized blood pressure estimation using photoplethysmography: a transfer learning approach. *IEEE J. Biomed. Health Inform.* **26**, 218–228 (2022). 10.1109/JBHI.2021.3085526 34077378

[125] Wu, D. et al. Continuous cuff-less blood pressure estimation based on combined information using deep learning approach. *J. Med. Imaging Health Inf.* **8**, 1290–1299 (2018).

[126] Hsu, Y.-C., Li, Y.-H., Chang, C.-C. & Harfiya, L. N. Generalized deep neural network model for cuffless blood pressure estimation with photoplethysmogram signal only. *Sensors* **20**, 5668 (2020). 10.3390/s20195668 PMC7582614 33020401

[127] Song, K., Chung, K.-Y. & Chang, J.-H. Cuffless deep learning-based blood pressure estimation for smart wristwatches. *IEEE Trans. Instrum. Meas.* **69**, 4292–4302 (2020).

[128] Fan, X., Wang, H., Xu, F., Zhao, Y. & Tsui, K.-L. Homecare-oriented intelligent long-term monitoring of blood pressure using electrocardiogram signals. *IEEE Trans. Ind. Inf.* **16**, 7150–7158 (2020).

[129] Panwar, M., Gautam, A., Biswas, D. & Acharyya, A. PP-Net: a deep learning framework for PPG-based blood pressure and heart rate estimation. *IEEE Sens. J.* **20**, 10000–10011 (2020).

[130] Chuang, C.-C., Lee, C.-C., Yeng, C.-H., So, E.-C. & Chen, Y.-J. Attention mechanism-based convolutional long short-term memory neural networks to electrocardiogram-based blood pressure estimation. *Appl. Sci.* **11**, 12019 (2021).

[131] Fan, X., Wang, H., Zhao, Y., Li, Y. & Tsui, K. L. An adaptive weight learning-based multitask deep network for continuous blood pressure estimation using electrocardiogram signals. *Sensors* **21**, 1595 (2021). 10.3390/s21051595 PMC7956522 33668778

[132] Bernard, D., Msigwa, C. & Yun, J. Toward IoT-based medical edge devices: PPG-based blood pressure estimation application. *IEEE Internet Things J.* **10**, 5240–5255 (2023).

[133] Jia, M., Qin, Y., Song, C., Yue, Z. & Ding, S. CEBPM: a cloud-edge collaborative noncontact blood pressure estimation model. *IEEE Trans. Instrum. Meas.* **71**, 1–12 (2022).

[134] Kim, D.-K., Kim, Y.-T., Kim, H. & Kim, D.-J. DeepCNAP: a deep learning approach for continuous noninvasive arterial blood pressure monitoring using photoplethysmography. *IEEE J. Biomed. Health Inform.* **26**, 3697–3707 (2022). 10.1109/JBHI.2022.3172514 35511844

[135] Roy, M. S., Gupta, R. & Sharma, K. D. BePCon: a photoplethysmography-based quality-aware continuous beat-to-beat blood pressure measurement technique using deep learning. *IEEE Trans. Instrum. Meas.* **71**, 1–9 (2022).

[136] Delfan, N. & Forouzanfar, M. Hybrid deep morpho-temporal framework for oscillometric blood pressure measurement. *IEEE J. Biomed. Health Inform.* **27**, 5293–5301 (2023). 10.1109/JBHI.2023.3310868 37651480

[137] Joung, J. et al. Continuous cuffless blood pressure monitoring using photoplethysmography-based PPG2BP-net for high intrasubject blood pressure variations. *Sci. Rep.* **13**, 8605 (2023). 10.1038/s41598-023-35492-y PMC10224938 37244974

[138] Kyung, J. et al. Deep-learning-based blood pressure estimation using multi channel photoplethysmogram and finger pressure with attention mechanism. *Sci. Rep.* **13**, 9311 (2023). 10.1038/s41598-023-36068-6 PMC10250382 37291140

[139] Shaikh, M. R. & Forouzanfar, M. Dual-stream CNN-LSTM architecture for cuffless blood pressure estimation from PPG and ECG Signals: a PulseDB study. *IEEE Sens. J.* **25**, 4006–4014 (2025).

[140] Sadrawi, M. et al. Genetic deep convolutional autoencoder applied for generative continuous arterial blood pressure via photoplethysmography. *Sensors* **20**, 3829 (2020). 10.3390/s20143829 PMC7412242 32660088

[141] Li, Z. & He, W. A continuous blood pressure estimation method using photoplethysmography by GRNN-based model. *Sensors* **21**, 7207 (2021). 10.3390/s21217207 PMC8587576 34770514

[142] Liu, Z., Zhang, Y. & Zhou, C. BiGRU-attention for continuous blood pressure trends estimation through single channel PPG. *Comput. Biol. Med.* **168**, 107795 (2024). 10.1016/j.compbiomed.2023.107795 38056206

[143] Liu, W. et al. Central aortic blood pressure waveform estimation with a temporal convolutional network. *IEEE J. Biomed. Health Inform.* **27**, 3622–3632 (2023). 10.1109/JBHI.2023.3268886 37079413

[144] Ma, C. et al. KD-Informer: a cuff-less continuous blood pressure waveform estimation approach based on single photoplethysmography. *IEEE J. Biomed. Health Inform.* **27**, 2219–2230 (2023). 10.1109/JBHI.2022.3181328 35700247

[145] Ma, C., Guo, L., Zhang, H., Liu, Z. & Zhang, G. DiffCNBP: lightweight diffusion model for IoMT-based continuous cuffless blood pressure waveform monitoring using PPG. *IEEE Internet Things J.* **12**, 61–80 (2025).

[146] Lee, N., Kim, S.-H., Lee, M. & Woo, J. Advancing continuous blood pressure estimation with transformer on photoplethysmography in operation room. *IEEE Access* **12**, 90486–90500 (2024).

[147] Miao, F. et al. Multi-sensor fusion approach for cuff-less blood pressure measurement. *IEEE J. Biomed. Health Inform.* **24**, 79–91 (2020). 10.1109/JBHI.2019.2901724 30892255

[148] Wang, C.-F. et al. Upper-arm photoplethysmographic sensor with one-time calibration for long-term blood pressure monitoring. *Biosensors* **13**, 321 (2023). 10.3390/bios13030321 PMC10046397 36979533

[149] Mukkamala, R. et al. Evaluation of the accuracy of cuffless blood pressure measurement devices: challenges and proposals. *Hypertension* **78**, 1161–1167 (2021). 10.1161/HYPERTENSIONAHA.121.17747 PMC8516718 34510915

[150] Association for the Advancement of Medical Instrumentation. American national standards for electronic or automated sphygmomanometers. *ANSI/AAMI SP 10-1987* (1987).

[151] Association, I. S. *IEEE Standard for Wearable, Cuffless Blood Pressure Measuring Devices—Amendment 1. IEEE Std 1708a-2019* (Amendment to IEEE Std 1708-2014) 1–35 (2019).

[152] O’Brien, E. et al. The British Hypertension Society protocol for the evaluation of blood pressure measuring devices. *J. Hypertens.* **11**, S43–s62 (1993). 10.1097/00004872-199306000-00013 8397248

[153] Mukkamala, R. et al. The microsoft research aurora project: important findings on cuffless blood pressure measurement. *Hypertension* **80**, 534–540 (2023). 10.1161/HYPERTENSIONAHA.122.20410 PMC9931644 36458550

[154] Harfiya, L. N., Chang, C.-C. & Li, Y.-H. Continuous blood pressure estimation using exclusively photopletysmography by LSTM-based signal-to-signal translation. *Sensors* **21**, 2952 (2021). 10.3390/s21092952 PMC8122812 33922447

[155] Whiting, P. F. et al. Quadas-2: a revised tool for the quality assessment of diagnostic accuracy studies. *Ann. Intern. Med.* **155**, 529–536 (2011). 10.7326/0003-4819-155-8-201110180-00009 22007046

[156] Wolff, R. F. et al. Probast: a tool to assess the risk of bias and applicability of prediction model studies. *Ann. Intern. Med.* **170**, 51–58 (2019). 10.7326/M18-1376 30596875

[157] Weber-Boisvert, G., Gosselin, B. & Sandberg, F. Intensive care photoplethysmogram datasets and machine-learning for blood pressure estimation: generalization not guarantied. *Front. Physiol.* **14**, 1126957 (2023). 10.3389/fphys.2023.1126957 PMC10017741 36935753

[158] Pielmus, A.-G. et al. Surrogate based continuous noninvasive blood pressure measurement. *Biomed. Eng.* **66**, 231–245 (2021). 10.1515/bmt-2020-0209 33565285

[159] Mehta, S., Kwatra, N., Jain, M. & McDuff, D. Examining the challenges of blood pressure estimation via photoplethysmogram. *Sci. Rep.* **14**, 18318 (2024). 10.1038/s41598-024-68862-1 PMC11306225 39112533

[160] Rovas, G., Bikia, V. & Stergiopulos, N. Quantification of the phenomena affecting reflective arterial photoplethysmography. *Bioengineering* **10**, 460 (2023). 10.3390/bioengineering10040460 PMC10136360 37106647

[161] Li, C. et al. PracticalBP: continuous cuffless blood pressure monitoring with only one record for calibration. In *Proc. ACM on Interactive, Mobile, Wearable and Ubiquitous Technologies* Vol. 9 (2025).

[162] Chandrasekhar, A., Yavarimanesh, M., Natarajan, K., Hahn, J.-O. & Mukkamala, R. Ppg sensor contact pressure should be taken into account for cuff-less blood pressure measurement. *IEEE Trans. Biomed. Eng.* **67**, 3134–3140 (2020). 10.1109/TBME.2020.2976989 PMC8856571 32142414

[163] Hickey, M., Phillips, J. P. & Kyriacou, P. A. Investigation of peripheral photoplethysmographic morphology changes induced during a hand-elevation study. *J. Clin. Monit. Comput.* **30**, 727–736 (2016). 10.1007/s10877-015-9761-0 26318315

[164] Shaltis, P. A., Reisner, A. T. & Asada, H. H. Cuffless blood pressure monitoring using hydrostatic pressure changes. *IEEE Trans. Biomed. Eng.* **55**, 1775–1777 (2008). 10.1109/tbme.2008.919142 18714843

[165] Colburn, D. A. M. et al. A method for blood pressure hydrostatic pressure correction using wearable inertial sensors and deep learning. *NPJ Biosens.* **2**, 5 (2025). 10.1038/s44328-024-00021-y PMC11785522 39897702

[166] Zhang, Y. et al. Artificial intelligence-enhanced wearable blood pressure monitoring in resource-limited settings: A co-design of sensors, model, and deployment. *Nanomicro Lett.* **18**, 164 (2026). 10.1007/s40820-025-02003-9 PMC12765800 41486315

[167] Abdollahzadeh, H. et al. Peak-independent cuffless blood pressure monitoring using a smart sock: The role of temporal lag modeling in foot-based PPG. *Sensors* **26**, 1269 (2026). 10.3390/s26041269 PMC12944016 41755207

[168] Carek, A. M. & Inan, O. T. Robust sensing of distal pulse waveforms on a modified weighing scale for ubiquitous pulse transit time measurement. *IEEE Trans. Biomed. Circuits Syst.* **11**, 765–772 (2017). 10.1109/TBCAS.2017.2683801 PMC5571434 28541911

[169] Mohammadpoor Faskhodi, M. et al. On the use of fractional calculus to improve the pulse arrival time (PAT) detection when using photoplethysmography (PPG) and electrocardiography (ECG) signals. *PLoS ONE* **19**, 1–25 (2024). 10.1371/journal.pone.0298354 PMC10871495 38363753

[170] Newlin, D. B. & Levenson, R. W. Pre-ejection period: measuring beta-adrenergic influences upon the heart. *Psychophysiology* **16**, 546–553 (1979). 10.1111/j.1469-8986.1979.tb01519.x 229507

[171] Guo, Z. et al. Sigma-ppg: Statistical-prior informed generative masking architecture for PPG foundation model. arxiv 10.48550/arXiv.2601.21031 (2026).

[172] Buxi, D., Redouté, J.-M. & Yuce, M. R. A survey on signals and systems in ambulatory blood pressure monitoring using pulse transit time. *Physiol. Meas.* **36**, R1–26 (2015). 10.1088/0967-3334/36/3/R1 25694235

[173] Mitchell, M. et al. Model cards for model reporting. In *Proc. Conference on Fairness, Accountability, and Transparency*, FAT* ’19, 220–229 10.1145/3287560.3287596 (ACM, 2019).

[174] Elgendi, M. et al. Photoplethysmography features correlated with blood pressure changes. *Diagnostics* **14**, https://www.mdpi.com/2075-4418/14/20/2309 (2024). 10.3390/diagnostics14202309 PMC11506471 39451632

[175] Stergiou, G. et al. European society of hypertension recommendations for the validation of cuffless blood pressure measuring devices: European society of hypertension working group on blood pressure monitoring and cardiovascular variability. *J. Hypertens.* **41**, 2074–2087 (2023). 10.1097/HJH.0000000000003483 37303198

[176] Elgendi, M. et al. Recommendations for evaluating photoplethysmography-based algorithms for blood pressure assessment. *Commun. Med.* **4**, 140 (2024). 10.1038/s43856-024-00555-2 PMC11245506 38997447

[177] Hodson, T. O. Root mean square error (RMSE) or mean absolute error (MAE): when to use them or not. *Geosci. Model Dev. Discuss.* **15**, 1–10 (2022).

[178] Podder, I. et al. Transfer learning for monitoring continuous blood pressure changes: Personalizing for SCI individuals. Authorea Preprints (2025).

[179] Rizal, S. & Ana Rahma, Y. GRU-based fusion models for enhanced blood pressure estimation from PPG signals. *IEEE Access* **12**, 80317–80326 (2024).

[180] Welykholowa, K. et al. Multimodal photoplethysmography-based approaches for improved detection of hypertension. *J. Clin. Med.* https://www.mdpi.com/2077-0383/9/4/1203 (2020). 10.3390/jcm9041203 PMC7230564 32331360

[181] Bird, K. et al. Assessment of hypertension using clinical electrocardiogram features: a first-ever review. *Front. Med.* **7**, 10.3389/fmed.2020.583331 (2020). PMC7746856 33344473

[182] Qin, K., Huang, W., Zhang, T. & Tang, S. Machine learning and deep learning for blood pressure prediction: a methodological review from multiple perspectives. *Artif. Intell. Rev.* **56**, 8095–8196 (2023).

[183] Sastimoglu, Z. et al. Wearable PPG based BP estimation methods: a systematic review and meta-analysis. *IEEE J. Biomed. Health Inform.* **29**, 2439–2452 (2024). 10.1109/JBHI.2024.3499834 40030275

[184] Cohen, J. B. et al. Cuffless devices for the measurement of blood pressure: a scientific statement from the American Heart Association. *Hypertension* **83**, e00254 (2026). 10.1161/HYP.0000000000000254 PMC13335599 41376592

[185] Huang, Y. et al. Camera-based blood pressure monitoring based on multisite and multiwavelength pulse transit time features. *IEEE Trans. Instrum. Meas.* **73**, 1–14 (2024).

[186] Sultan, M. A. & Saadeh, W. Continuous patient-independent estimation of respiratory rate and blood pressure using robust spectro-temporal features derived from photoplethysmogram only. *IEEE Open J. Eng. Med. Biol.* **5**, 637–649 (2024). 10.1109/OJEMB.2023.3329728 PMC11342923 39184965

