# Supplementary material for: Towards trustworthy AI-driven cuffless blood pressure monitoring
Source: NPJ Digit Med. 2026 Jun 25;9:794. doi: 10.1038/s41746-026-02898-7 (PMC13669345; doi:10.1038/s41746-026-02898-7)
Supplement: Supplementary file 1 [file 41746_2026_2898_MOESM1_ESM.pdf]

Supplementary Information

Towards Trustworthy AI-driven Cuffless Blood Pressure Monitoring

Ana Cisnal[1,2,+], Itilekha Podder[1,3,+], Leoni Grossmann[1,4], Kanika Dheman[1,5], Mohamed Elgendi[6,7], Marco Valgimigli[8], Diego Paez-Granados[1,9,*]

[1] Spinal Cord Artificial Intelligence (SCAI) Lab, D-HEST, ETH Zürich, 8092 Zürich, Switzerland
[2]Institute of Advanced Production Technologies, Valladolid University, Paseo Prado de la Magdalena 3-5, Valladolid, 47011, Spain
[3]Faculty of Informatics, Eötvös Loránd University, Egyetem tér 1-3, Budapest, 1053, Hungary
[4]Department of Clinical Neuroscience, Karolinska Institutet, Retzius väg 8, Stockholm, 171 65, Sweden
[5]Center for Project Based Learning Lab, ETH Zurich, Gloriastrasse 35, Zurich, 8092, Switzerland
[6]Department of Biomedical Engineering and Bi otechnology, Khalifa University of Science and Technology, Abu Dhabi, UAE
[7]Healthcare Engineering Innovation Group (HEIG), Khalifa University of Science and Technology, Abu Dhabi, UAE
[8]Department of Biomedical Sciences, University of Italian Switzerland, Cardiocentro Ticino Institute, Ente Ospedaliero Cantonale, Via Tesserete 46, Lugano, 6900, Switzerland
[9]Digital Health Care and Rehabilitation, Swiss Paraplegic Research (SPF), Guido A Zach. 4, Notwill, 6207, Switzerland

[+]These authors contributed equally
[*]Correspondence: diego.paez@hest.ethz.ch

Supplementary Note 1: PRISMA Checklist

Section and Topic	Item #	Checklist item	Location where item is reported (Line number)
**TITLE**			
Title	1	Identify the report as a systematic review.	1
**ABSTRACT**			
Abstract	2	See the PRISMA 2020 for Abstracts checklist.	-
**INTRODUCTION**			
Rationale	3	Describe the rationale for the review in the context of existing knowledge.	20-44
Objectives	4	Provide an explicit statement of the objective(s) or question(s) the review addresses.	45-51
**METHODS**			
Eligibility criteria	5	Specify the inclusion and exclusion criteria for the review and how studies were grouped for the syntheses.	592-596
Information sources	6	Specify all databases, registers, websites, organisations, reference lists and other sources searched or consulted to identify studies. Specify the date when each source was last searched or consulted.	591-592
Search strategy	7	Present the full search strategies for all databases, registers and websites, including any filters and limits used.	597-598
Selection process	8	Specify the methods used to decide whether a study met the inclusion criteria of the review, including how many reviewers screened each record and each report retrieved, whether they worked independently, and if applicable, details of automation tools used in the process.	596-597
Data collection process	9	Specify the methods used to collect data from reports, including how many reviewers collected data from each report, whether they worked independently, any processes for obtaining or confirming data from study investigators, and if applicable, details of automation tools used in the process.	596-597
Data items	10a	List and define all outcomes for which data were sought. Specify whether all results that were compatible with each outcome domain in each study were sought (e.g. for all measures, time points, analyses), and if not, the methods used to decide which results to collect.	592-593
	10b	List and define all other variables for which data were sought (e.g. participant and intervention characteristics, funding sources). Describe any assumptions made about any missing or unclear information.	-
Study risk of bias assessment	11	Specify the methods used to assess risk of bias in the included studies, including details of the tool(s) used, how many reviewers assessed each study and whether they worked independently, and if applicable, details of automation tools used in the process.	399-410
Effect	12	Specify for each outcome the effect measure(s)	-

1

Section and Topic	Item #	Checklist item	Location where item is reported (Line number)
measures		(e.g. risk ratio, mean difference) used in the synthesis or presentation of results.	
Synthesis methods	13a	Describe the processes used to decide which studies were eligible for each synthesis (e.g. tabulating the study intervention characteristics and comparing against the planned groups for each synthesis (item #5)).	-
	13b	Describe any methods required to prepare the data for presentation or synthesis, such as handling of missing summary statistics, or data conversions.	-
	13c	Describe any methods used to tabulate or visually display results of individual studies and syntheses.	-
	13d	Describe any methods used to synthesize results and provide a rationale for the choice(s). If meta-analysis was performed, describe the model(s), method(s) to identify the presence and extent of statistical heterogeneity, and software package(s) used.	605-616
	13e	Describe any methods used to explore possible causes of heterogeneity among study results (e.g. subgroup analysis, meta-regression).	610-612
	13f	Describe any sensitivity analyses conducted to assess robustness of the synthesized results.	605-606
Reporting bias assessment	14	Describe any methods used to assess risk of bias due to missing results in a synthesis (arising from reporting biases).	-
Certainty assessment	15	Describe any methods used to assess certainty (or confidence) in the body of evidence for an outcome.	612-616
**RESULTS**			
Study selection	16a	Describe the results of the search and selection process, from the number of records identified in the search to the number of studies included in the review, ideally using a flow diagram.	599-601
	16b	Cite studies that might appear to meet the inclusion criteria, but which were excluded, and explain why they were excluded.	-
Study characteristics	17	Cite each included study and present its characteristics.	59-61
Risk of bias in studies	18	Present assessments of risk of bias for each included study.	383-416
Results of individual	19	For all outcomes, present, for each study: (a) summary statistics for each group (where	93-110, 360-369

Section and Topic	Item #	Checklist item	Location where item is reported (Line number)
studies		appropriate) and (b) an effect estimate and its precision (e.g. confidence/credible interval), ideally using structured tables or plots.	
Results of syntheses	20a	For each synthesis, briefly summarise the characteristics and risk of bias among contributing studies.	399-410
	20b	Present results of all statistical syntheses conducted. If meta-analysis was done, present for each the summary estimate and its precision (e.g. confidence/credible interval) and measures of statistical heterogeneity. If comparing groups, describe the direction of the effect.	383-416
	20c	Present results of all investigations of possible causes of heterogeneity among study results.	406-416
	20d	Present results of all sensitivity analyses conducted to assess the robustness of the synthesized results.	383-416
Reporting biases	21	Present assessments of risk of bias due to missing results (arising from reporting biases) for each synthesis assessed.	406-410
Certainty of evidence	22	Present assessments of certainty (or confidence) in the body of evidence for each outcome assessed.	383-398
**DISCUSSION**			
Discussion	23a	Provide a general interpretation of the results in the context of other evidence.	418-434
	23b	Discuss any limitations of the evidence included in the review.	418-434
	23c	Discuss any limitations of the review processes used.	-
	23d	Discuss implications of the results for practice, policy, and future research.	550-587
**OTHER INFORMATION**			
Registration and protocol	24a	Provide registration information for the review, including register name and registration number, or state that the review was not registered.	-
	24b	Indicate where the review protocol can be accessed, or state that a protocol was not prepared.	-
	24c	Describe and explain any amendments to information provided at registration or in the protocol.	-
Support	25	Describe sources of financial or non-financial support for the review, and the role of the funders or sponsors in the review.	620-622
Competing interests	26	Declare any competing interests of review authors.	629-630
Availability of data, code and other materials	27	Report which of the following are publicly available and where they can be found: template data collection forms; data extracted from included studies; data used for all analyses; analytic code; any other materials	-

3

Section and Topic	Item #	Checklist item	Location where item is reported (Line number)
		used in the review.	

From: Page MJ, McKenzie JE, Bossuyt PM, Boutron I, Hoffmann TC, Mulrow CD, et al. The PRISMA 2020 statement: an updated guideline for reporting systematic reviews. BMJ 2021;372:n71. doi: 10.1136/bmj.n71. This work is licensed under CC BY 4.0. To view a copy of this license, visit https://creativecommons.org/licenses/by/4.0/

Supplementary Note 2: Search strategies

Database-specific full search strategies

- PubMed: *continu* AND (Cuffless OR cuff-less OR PPG OR Photoplethysmogra*) AND ("Blood pressure" OR BP OR hypertension OR hypotension OR hypertensive OR hypotensive) AND (learning OR "artificial intelligence" OR algorithm OR estimation OR prediction OR regression) AND valid**

- IEEE Xplore: *"Full Text & Metadata":continu* AND (Cuffless OR "Full Text & Metadata":cuff-less OR "Full Text & Metadata":PPG OR "Full Text & Metadata":Photoplethysmogra*) AND ("Blood pressure" OR "Full Text & Metadata":BP OR "Full Text & Metadata":hypertension OR "Full Text & Metadata":hypotension OR "Full Text & Metadata":hypertensive OR "Full Text & Metadata":hypotensive) AND (learning OR "Full Text & Metadata":"artificial intelligence" OR "Full Text & Metadata":algorithm OR "Full Text & Metadata":estimation OR "Full Text & Metadata":prediction OR "Full Text & Metadata":regression) AND "Full Text & Metadata":valid**

Supplementary Table 1: List of acronyms and their meanings

Acronym	Meaning
AAMI	Association for the Advancement of Medical Instrumentation
ABP	Arterial Blood Pressure
ANN	Artificial Neural Network
ANSI	American National Standards Institute
APG	Acceleration Plethysmogram
BHS	British Hypertension Society
BiGRU	Bidirectional Gated Recurrent Unit
BiLSTM	Bidirectional Long Short-Term Memory
BP	Blood Pressure
BPMD	Blood Pressure Measuring Device
CI	Confidence Interval
CNN	Convolutional Neural Network
CP	Cumulative Percentages
CV	Cross-Validation
CVD	Cardiovascular Disease
DBP	Diastolic Blood Pressure
DNN	Deep Neural Network
DL	Deep Learning
DL RE	DerSimonian–Laird Random-Effects
ECG	Electrocardiogram
GAN	Generative Adversarial Network
GPR	Gaussian Process Regression
GRNN	General Regression Neural Network
KNN	K-Nearest Neighbors
HK	Hartung–Knap
HTN	Hypertension
iPPG	Imaging Photoplethysmography
IPG	Impedance Plethysmography
ISO	International Organization for Standardization
LOOCV	Leave-One-Out Cross-Validation
LSTM	Long Short-Term Memory
MAD	Mean Absolute Difference
MAP	Mean Arterial Pressure
MAPD	Mean Absolute Percentage Difference
MD	Mean Difference
ME	Mean Error
MEC	Mechano-Electric Coupling
MIMIC	Medical Information Mart for Intensive Care
ML	Machine Learning
MWPPG	Multi-Wavelength Photoplethysmography
PAT	Pulse Arrival Time
PEESE	Precision-effect Estimate with Standard Error
PET	Precision-effect Test
PPG	Photoplethysmography
PPW	Pulse Pressure Waveform
PRISMA	Reporting Items for Systematic Reviews and Meta-Analyses
PTT	Pulse Transit Time
PWA	Pulse Wave Analysis
PWV	Pulse Wave Velocity
PZT	Piezoelectric Transducer

RE	Random Effect
REML	Restricted Maximum Likelihood
RMSE	Root Mean Square Error
RNN	Recurrent Neural Network
rPPG	Remote Photoplethysmography
SBP	Systolic Blood Pressure
SCI	Spinal Cord Injury
SE	Standard Error
SD	Standard Deviation
SpO_2	Peripheral Capillary Oxygen Saturation
SVR	Support Vector Regression
TF	Time-Frequency
TPS	Two-pulse Synthesis
UCI	University of California, Irvine
UQVS	University of Queensland Vital Signs
VPG	Velocity Plethysmogram

Supplementary Figure 1: BP-Model-Card for Calibration-free Models

BP-Model-Card for Calibration-free Methods

MODULE 0: CLINICAL PURPOSE AND USE CONTEXT

Model name / version: __________
Estimation family: [ ] Hemodynamics-derived [ ] Data-driven BP regression [ ] ABP waveform reconstruction
Calibration paradigm: [ ] Calibration-free [ ] Calibration-based
Model type: [ ] Machine learning (ML) [ ] Deep learning (DL) [ ] Hybrid [ ] Other: __________
Input data type: [ ] Handcrafted features [ ] Time series [ ] Images [ ] Demographic [ ] Other: __________
Output variable: [ ] SBP [ ] DBP [ ] MAP
Signal modality: [ ] PPG [ ] ECG [ ] BCG [ ] BioZ [ ] IPG [ ] iPPG [ ] Other: __________
Sensor specification: __________
Sensor location: __________
Intended use: [ ] Research use only [ ] Home / ambulatory monitoring [ ] Adjunctive clinical monitoring [ ] Clinical pilot / prospective evaluation

MODULE 1: DATASET AND COHORT REPORTING

Dataset source: [ ] Private [ ] Public: __________
Participants (number of subjects): ______ (at least 85 participants)
Recording sessions: ______ **Total paired BP samples:** ______

Population type: [ ] Healthy [ ] Hypertensive [ ] ICU [ ] Other: __________
Age (years): mean ± SD ____ ; range ____
Sex: % female ____ ; % male ____
BMI (kg/m^2): mean ± SD ____
Height (cm): mean ± SD ____
Weight (kg): mean ± SD ____
Ethnicity / geographic region: __________
Medical history / comorbidities reported: [ ] Yes [ ] No
Medication reported: [ ] Yes [ ] No; if yes: class / dose / regimen _______
Known sensitivity: [ ] Motion [ ] Low perfusion [ ] Arrhythmia [ ] Sensor displacement [ ] Skin tone [ ] Other: __________
Skin tone / Fitzpatrick: __________ (if PPG-based)
Ambient light conditions: __________ (if PPG-based)

Measurement phases (%):
Ambulatory: ___ | In-clinic: ___ | Dynamic: ___ | Long-term: ___ |Sleep: ___
BP profiles (%):
Hypotension: ___ | Normal: ___ | Elevated: ___ | Stage 1: ___ | Stage 2: ____
SBP (mmHg): mean ± SD ____ ; range ______
DBP (mmHg): mean ± SD ____ ; range ______
Baseline SBP (mmHg): mean ± SD ____ ; range ______
Baseline DBP (mmHg): mean ± SD ____ ; range ______
ΔSBP (mmHg): mean ± SD ____ [ ] Subject-specific [ ] Population-based
ΔDBP (mmHg): mean ± SD ____ [ ] Subject-specific [ ] Population-based
(a) Example BP distribution histogram
Preprocessing pipeline: __________
Signal quality filtering / exclusion criteria: __________
Fiducial points / feature extraction: __________
Excluded samples after cleaning: ______
BP distribution before/after cleaning reported: [ ] Yes [ ] No

BP distribution example:

x-axis: Reference BP (mmHg)
y-axis: Frequency / Count

MODULE 2. VALIDATION STRATEGY

Evaluation method: [ ] Subject-wise hold-out [ ] Subject-wise CV [ ] LOSO / LOOCV [ ] Record-wise split [ ] Sample-wise split
Data split details: __________
Unseen-subject test set: [ ] Yes [ ] No
External validation (independent cohort / site): [ ] Yes [ ] No
Reference standard: __________
Reference device (manufacturer/model): _______

Measurement conditions: [ ] Resting [ ] Ambulatory [ ] Dynamic [ ] Long-term [ ] ICU monitoring [ ] Other: ______
Body position: __________
Activity state: __________
Rest period prior to measurement: __________
Baseline comparator: __________
Comparator result: __________

Minimum acceptable standard met: _______ [ ] Subject-wise validation

MODULE 3. PERFORMANCE REPORTING ***(Calibration-free models)***

Models evaluated: [] Baseline model [] Proposed / final model

Standardized performance reporting table:

Group	ΔBP±SD (mmHg)	MD±SD (mmHg)	MAD (mmHg)	MAPD (%)	CP5 (%)	CP10 (%)	CP15 (%)	R
All								
Normal								
Prehypertension								
Hypertension Stage 1								
Hypertension Stage 2								

Recommended visualization formats:

(b) Example scatter plot

x-axis: Reference BP (mmHg)
y-axis: Estimated cuffless BP (mmHg)

(c) Example Bland--Altman plot

x-axis: Mean of reference and estimated BP (mmHg)
y-axis: Estimated BP - Reference BP (mmHg)

Supplementary Figure 1: **Module 0** defines the clinical purpose and use context. **Module 1** specifies dataset and cohort reporting requirements, including BP distribution, preprocessing, and exclusion criteria, with an example histogram of reference BP values. **Module 2** summarizes the validation strategy, including evaluation protocol, reference standard, and baseline comparator. **Module 3** provides a standardized performance reporting format for calibration-free models, together with recommended scatter and Bland–Altman plots for comparing estimated and reference BP.

Supplementary Figure 2: BP-Model-Card for Calibration-based Models

BP-Model-Card for Calibration-based Methods

MODULE 0: CLINICAL PURPOSE AND USE CONTEXT

Model name / version: _________
Estimation family: [ ] Hemodynamics-derived [ ] Data-driven BP regression [ ] ABP waveform reconstruction
Calibration paradigm: [ ] Calibration-free [ ] Calibration-based
Model type: [ ] Machine learning (ML) [ ] Deep learning (DL) [ ] Hybrid [ ] Other: _________
Input data type: [ ] Handcrafted features [ ] Time series [ ] Images [ ] Demographic [ ] Other: _________
Output variable: [ ] SBP [ ] DBP [ ] MAP
Signal modality: [ ] PPG [ ] ECG [ ] BCG [ ] BioZ [ ] IPG [ ] iPPG [ ] Other: _________
Sensor specification: _________
Sensor location: _________
Intended use: [ ] Research use only [ ] Home / ambulatory monitoring [ ] Adjunctive clinical monitoring [ ] Clinical pilot / prospective evaluation

MODULE 1: DATASET AND COHORT REPORTING

Dataset source: [ ] Private [ ] Public: _________
Participants (number of subjects): _____ (at least 85 participants)
Recording sessions: _____ **Total paired BP samples:** _____

Population type: [ ] Healthy [ ] Hypertensive [ ] ICU [ ] Other: _________
Age (years): mean ± SD ____; range ____
Sex: % female ____; % male ____
BMI (kg/m²): mean ± SD ____
Height (cm): mean ± SD ____
Weight (kg): mean ± SD ____
Ethnicity / geographic region: _________
Medical history / comorbidities reported: [ ] Yes [ ] No
Medication reported: [ ] Yes [ ] No; if yes: class / dose / regimen ______
Known sensitivity: [ ] Motion [ ] Low perfusion [ ] Arrhythmia [ ] Sensor displacement [ ] Skin tone [ ] Other: _________
Skin tone / Fitzpatrick: _________ (if PPG-based)
Ambient light conditions: _________ (if PPG-based)

Measurement phases (%):
Ambulatory: ___ | In-clinic: ___ | Dynamic: ___ | Long-term: ___ |Sleep:___
BP profiles (%):
Hypotension: ___ | Normal: ___ | Elevated: ___ | Stage 1: ___ | Stage 2: ____
SBP (mmHg): mean ± SD ____; range _____
DBP (mmHg): mean ± SD ____; range _____
Baseline SBP (mmHg): mean ± SD ____; range _____
Baseline DBP (mmHg): mean ± SD ____; range _____
ΔSBP (mmHg): mean ± SD ____ [ ] Subject-specific [ ] Population-based
ΔDBP (mmHg): mean ± SD ____ [ ] Subject-specific [ ] Population-based

Preprocessing pipeline: _________
Signal quality filtering / exclusion criteria: _________
Fiducial points / feature extraction: _________
Excluded samples after cleaning: _________
BP distribution before/after cleaning reported: [ ] Yes [ ] No

BP distribution example:

(a) Example BP distribution histogram

x-axis: Reference BP (mmHg)
y-axis: Frequency / Count

(b) Example calibration-relative BP distribution

x-axis: ΔBP from calibration (mmHg)
y-axis: Frequency / Count

MODULE 2. VALIDATION STRATEGY

Evaluation method: [ ] Subject-wise hold-out [ ] Subject-wise CV [ ] LOSO / LOOCV [ ] Record-wise split [ ] Sample-wise split
Data split details: _________
Unseen-subject test set: [ ] Yes [ ] No
External validation (independent cohort / site): [ ] Yes [ ] No
Reference standard: _________
Reference device (manufacturer/model): ______

Measurement conditions: [ ] Resting [ ] Ambulatory [ ] Dynamic [ ] Long-term [ ] ICU monitoring [ ] Other: _____
Body position: _________
Activity state: _________
Rest period prior to measurement: _________
Baseline comparator: _________
Comparator result: _________

Minimum acceptable standard met: ______ [ ] Subject-wise validation

MODULE 3. PERFORMANCE REPORTING *(Calibration-based models)*

Models evaluated: [] Baseline model [] Proposed / final model

Calibration condition: _________
Calibration type: [ ] One-point [ ] Two-point [ ] Multi-point [ ] Other:_____
Static test: [ ] Yes [ ] No **Dynamic test:** [ ] Yes [ ] No
Time since calibration evaluated: [ ] Yes [ ] No; if yes: **Time interval** (days): ______
Recalibration trigger (if any): ________

Standardized performance reporting table:

Condition	ΔBP±SD (mmHg)	MD±SD (mmHg)	MAD (mmHg)	MAPD (%)	CP5 (%)	CP10 (%)	CP15 (%)	R
Static								
Dynamic								
Time after calibration								

Recommended visualization formats:

(e) Example scatter plot

x-axis: Reference ΔBP from calibration (mmHg)
y-axis: Estimated ΔBP from calibration (mmHg)

(f) Example Bland--Altman plot

x-axis: Mean of reference and estimated ΔBP (mmHg)
y-axis: Estimated Δ BP - Reference ΔBP (mmHg)

Supplementary Figure 2: **Module 0** defines the clinical purpose and use context. **Module 1** specifies dataset and cohort reporting, including BP distributions and calibration-relative BP changes. **Module 2** summarizes the validation strategy, including subject-wise evaluation, measurement conditions (resting, ambulatory, dynamic), and baseline comparator. **Module 3** provides standardized performance reporting for calibration-based models across static, dynamic, and post-calibration conditions, with recommended calibration-relative visualizations: (a) scatter plot (ΔBP estimated vs. ΔBP reference) and (b) Bland–Altman plot (difference vs. mean of ΔBP).

Supplementary Note 3: Representative model-card examples

(Qiao, et al., 2025): A two-branch framework for BP estimation using PPG signals

MODULE 0: CLINICAL PURPOSE AND USE CONTEXT

Model name / version: Two-branch PPG BP Estimation Framework
Estimation family: [ ] Hemodynamics-derived [x] Data-driven BP regression [ ] ABP waveform reconstruction
Calibration paradigm: [ ] Calibration-free [x] Calibration-based
Model type: [ ] Machine learning (ML) [ ] Deep learning (DL) [x] Hybrid [ ] Other
Input data type: [x] Handcrafted features [x] Time series [x] Images [ ] Demographic [ ] Other
Output variable: [x] SBP [x] DBP [ ] MAP
Signal modality: [x] PPG [ ] ECG [ ] BCG [ ] BioZ [ ] IPG [ ] iPPG [ ] Other:
Sensor specification: NA
Sensor location: left middle finger PPG
Intended use: [x] Research use only [ ] Home / ambulatory monitoring [ ] Adjunctive clinical monitoring [ ] Clinical pilot / prospective evaluation

MODULE 1: DATASET AND COHORT REPORTING

Dataset source: [x] Private: HRSD [x] Public: MIMIC-IV
Participants (number of subjects): HRSD: 30; MIMIC-IV: 198
Recording sessions: 1 **Total paired BP samples:** 139 (HRSD)

Population type: [x] Healthy (HSRD) [ ] Hypertensive [x] ICU (MIMIC)
Age (years): range HRSD: 20-50
Sex: HRSD: % female 50 ; % male 50; MIMIC-IV: not reported
BMI (kg/m²): NA
Height (cm): NA
Weight (kg): NA
Ethnicity / geographic region: NA
Medical history / comorbidities reported: [ ] Yes [x] No
Medication reported: [ ] Yes [x] No
Known sensitivity: [ ] Motion [ ] Low perfusion [ ] Arrhythmia [ ] Sensor displacement [ ] Skin tone [ ] Other
Skin tone / Fitzpatrick: Asian skin tones (HRSD) (if PPG-based)
Ambient light conditions: NA (if PPG-based)

Measurement phases (%):
Ambulatory: 0 | In-clinic: 100 | Dynamic: 0 | Long-term: 0 |Sleep: 0
BP profiles (%):
Hypotension: NA | Normal: NA | Elevated: NA | Stage 1: NA | Stage 2: NA
SBP (mmHg): range 80-165
DBP (mmHg): range 40-100
Baseline SBP (mmHg): NA
Baseline DBP (mmHg): NA
ΔSBP (mmHg): NA [ ] Subject-specific [ ] Population-based
ΔDBP (mmHg): NA [ ] Subject-specific [ ] Population-based

Preprocessing pipeline: 5th order Butterworth bandpass filter, 0.5-5 Hz passband, motion/artifact rejection, peak detection, abnormal waveform detection, visibility graph transformation, fine-grained cycle segmentation
Signal quality filtering / exclusion criteria: discontinuous signal, saturated amplitude, insufficient systolic peaks
Fiducial points / feature extraction: 72 features (time-domain, morphological and statistical)
Excluded samples after cleaning: NA
BP distribution before/after cleaning reported: [ ] Yes [x] No

BP distribution:
(a) BP distribution:

Not provided

(b) Calibration-relative BP distribution:

Not provided

MODULE 2. VALIDATION STRATEGY

Evaluation method: [x] Subject-wise hold-out [ ] Subject-wise CV [ ] LOSO / LOOCV [ ] Record-wise split [ ] Sample-wise split
Data split details: 85%/15% (train/test); CV-based tunning (only train)
Unseen-subject test set: [x] Yes [ ] No
External validation (independent cohort / site): [x] Yes [ ] No (MIMIC)
Reference standard: cuff BP reference (HRSD); invasive ABP (MIMIC)
Reference device (manufacturer/model): NA

Measurement conditions: [x] Resting (HSRD) [ ] Ambulatory [ ] Dynamic [ ] Long-term [x] ICU monitoring (MIMIC) [ ] Other
Body position: Seat/Lying
Activity state: Resting
Rest period prior to measurement: NA
Baseline comparator: NA
Comparator result: Two-branch framework with MobileViTv2 backbone for SBP and VGG19 backbone for DBP

Minimum acceptable standard met: AAMI, Grade A in BHS [x] Subject-wise validation

MODULE 3. PERFORMANCE REPORTING

Models evaluated: [] Baseline model [x] Proposed / final model

Calibration condition: Resting/Seated
Calibration type: [ ] One-point [ ] Two-point [ ] Multi-point [x] Other: Population-level supervised model training with intermittent cuff-reference labeling
Static test: [x] Yes [ ] No **Dynamic test:** [ ] Yes [x] No
Time since calibration evaluated: [ ] Yes [x] No; if yes: **Time interval** (days): -
Recalibration trigger (if any): None specified

Performance reporting table:

SBP / Condition	ΔBP±SD (mmHg)	MD±SD (mmHg)	MAD (mmHg)	MAPD (%)	CP5 (%)	CP10 (%)	CP15 (%)	R
Static (HRSD)	-	0.59±7.47	6.28	-	-	-	-	-
Static (MIMIC-IV)	-	-0.13±6.33	4.34	-	-	-	-	-
Dynamic	-	-	-	-	-	-	-	-
Time after calibration	-	-	-	-	-	-	-	-

DBP / Condition	ΔBP±SD (mmHg)	MD±SD (mmHg)	MAD (mmHg)	MAPD (%)	CP5 (%)	CP10 (%)	CP15 (%)	R
Static (HRSD)	-	0.45±6.77	4.98	-	-	-	-	-
Static (MIMIC-IV)	-	0.18±4.52	3.11	-	-	-	-	-
Dynamic				-	-	-	-	-
Time after calibration				-	-	-	-	-

Performance visualization (HRSD):

(e) Scatter plot

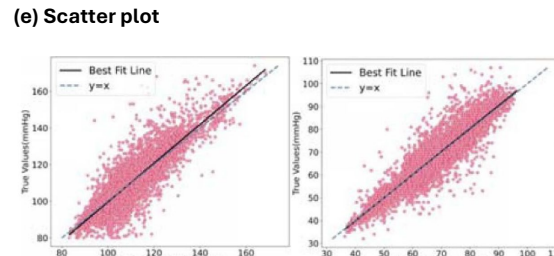

(f) Bland-Altman plot

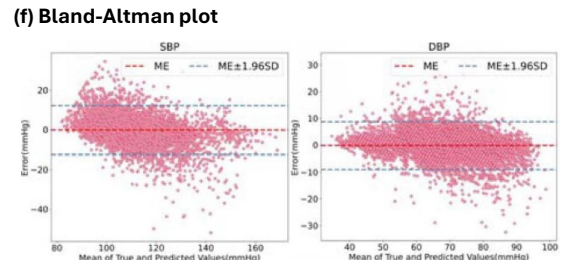

Supplementary Figure 3: Calibration-based model card corresponding to the study by Qiao et al. (2025): A two-branch framework for blood pressure estimation using photoplethysmography signals with deep learning and clinical prior physiological knowledge.

(Lee et al., 2024): Advancing Continuous Blood Pressure Estimation with Transformer on PPG

MODULE 0: CLINICAL PURPOSE AND USE CONTEXT

Model name / version: Transformer-based continuous BP estimation from PPG
Estimation family: [ ] Hemodynamics-derived [ ] Data-driven BP regression [x] ABP waveform reconstruction
Calibration paradigm: [x] Calibration-free [ ] Calibration-based
Model type: [ ] Machine learning (ML) [x] Deep learning (DL) [ ] Hybrid [ ] Other
Input data type: [ ] Handcrafted features [x] Time series [ ] Images [ ] Other
Output variable: [x] SBP [x] DBP [x] MAP
Signal modality: [x] PPG [ ] ECG [ ] BCG [ ] BioZ [ ] IPG [ ] iPPG [ ] Other
Sensor specification: Bedside patient monitoring systems
Sensor location: Finger
Intended use: [x] Research use only [ ] Home / ambulatory monitoring [ ] Adjunctive clinical monitoring [ ] Clinical pilot / prospective evaluation

MODULE 1: DATASET AND COHORT REPORTING

Dataset source: [x] Private: SCH BC [x] Public: MIMIC II & VitalDB
Participants (number of subjects): SCH BC: 273; MIMIC II: 8,746; VitalDB: 100
Recording sessions: Single perioperative/ICU monitoring sessions
Total paired BP samples: SCH BC: 47,320 segments; MIMIC II: 698,000 segments; VitalDB: not reported

Population type: [ ] Healthy [ ] Hypertensive [x] ICU [x] Other: surgical
Age (years): NA
Sex: NA
BMI (kg/m²): NA
Height (cm): NA
Weight (kg): NA
Ethnicity / geographic region: Republic of Korea + USA
Medical history / comorbidities reported: [ ] Yes [x] No
Medication reported: [ ] Yes [x] No;
Known sensitivity: [X] Motion [ ] Low perfusion [ ] Arrhythmia [X] Sensor displacement [ ] Skin tone [ ] Other
Skin tone / Fitzpatrick: NA (if PPG-based)
Ambient light conditions: NA (if PPG-based)

Measurement phases (%):
Ambulatory: 0| In-clinic: 100 | Dynamic: 0| Long-term: 0 |Sleep: 0
BP profiles (%): NA
SBP (mmHg): SCH BC:120.14 ±16.42 [75.39, 143.70]; MIMIC II: 129.63 ± 2.71 [55.56 – 84.28]
DBP (mmHg): SCH BC:64.79 ±5.71 [55.56, 84.28]; MIMIC II: 69.98 ± 2.28 [52.36 – 77.56]
Baseline SBP (mmHg): NA
Baseline DBP (mmHg): NA
ΔSBP (mmHg): NA [ ] Subject-specific [ ] Population-based
ΔDBP(mmHg): NA [ ] Subject-specific [ ] Population-based

Preprocessing pipeline: Visual inspection for motion/noise artifacts. Physiological threshold filtering. Savitzky-Golay filtering. Discrete wavelet transform denoising. Segmentation into 10-s windows.
Signal quality filtering / exclusion criteria: SBP < 80 or > 180 mmHg. DBP <50 or > 130 mmHg. HR<30 or > 300 bpm. Noise or motion corrupted recordings.
Fiducial points / feature extraction: No handcrafted features
Excluded samples after cleaning: SCH169;
BP distribution before/after cleaning reported: [x] Yes [ ] No

(a) BP distribution:

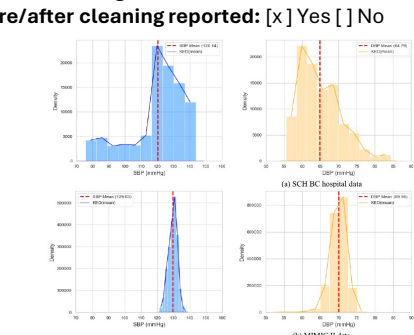

MODULE 2. VALIDATION STRATEGY

Evaluation method: [x] Subject-wise hold-out [ ] Subject-wise CV [ ] LOSO / LOOCV [ ] Record-wise split [ ] Sample-wise split
Data split details: 7:1:2 (train/validation/test)
Unseen-subject test set: [x] Yes [ ] No
External validation (independent cohort / site): [x] Yes [ ] No (MIMIC II and VitalDB)
Reference standard: ABP
Reference device (manufacturer/model): Bx50 monitor

Measurement conditions: [] Resting [ ] Ambulatory [ ] Dynamic [ ] Long-term [x] ICU monitoring (MIMIC) [x] Other: Under anesthesia (SCH BC)
Body position: supine or semi-recumbent ICU bed position
Activity state: sedated / perioperative monitoring
Rest period prior to measurement: NA
Baseline comparator: NA
Comparator result: Transformer

Minimum acceptable standard met: AAMI, IEEE, achieved Grade A under BHS standard [x] Subject-wise validation

MODULE 3. PERFORMANCE REPORTING

Models evaluated: [ ] Baseline model [x] Proposed / final model

Performance table (transformer model on SCH BC):

SBP/Group	ΔBP±SD (mmHg)	MD±SD (mmHg)	MAD (mmHg)	MAPD (%)	CP5 (%)	CP10 (%)	CP15 (%)	R
All	-	0.12±0.91	1.03	-	99.6	100	100	1.0
Normal	-	-	-	-	-	-	-	-
Prehypertension	-	-	-	-	-	-	-	-
HNT Stage 1	-	-	-	-	-	-	-	-
HTN Stage 2	-	-	-	-	-	-	-	

DBP/Group	ΔBP±SD (mmHg)	MD±SD (mmHg)	MAD (mmHg)	MAPD (%)	CP5 (%)	CP10 (%)	CP15 (%)	R
All	-	-0.64±0.68	0.92	-	99.9	100	100	0.99
Normal	-	-	-	-	-	-	-	-
Prehypertension	-	-	-	-	-	-	-	-
HNT Stage 1	-	-	-	-	-	-	-	-
HTN Stage 2	-	-	-	-	-	-	-	

Performance table (transformer model – external validation)

Group	ΔBP±SD (mmHg)	MD±SD (mmHg)	MAD (mmHg)	MAPD (%)	CP5 (%)	CP10 (%)	CP15 (%)	R
• SBP								
MIMIC II (all)		0.10±1.14	1.31	-	98.2	100	100	0.93
Vital DB (all)		-	6.62	-	-	-	-	-
• DBP		-	6.62	-	-	-	-	-
MIMIC II (all)		-0.42±0.82	1.30		99.9	100	100	0.75
Vital DB (all)		-	0.80	-	-	-	-	-

Performance visualization:

(b) Scatter plot (transformer model on SCH BC)

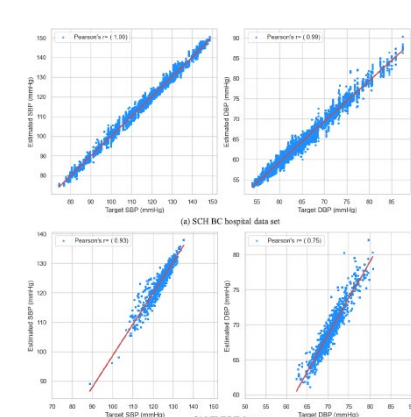

(c) Bland-Altman plot

Not provided

Supplementary Figure 4: Calibration-free model card corresponding to the study by Lee et al. (2024): Advancing Continuous Blood Pressure Estimation with Transformer on Photoplethysmography in Operating Room.

(Jamil et al., 2025): BP estimation using self-attention mechanism built-in ResUNet on PulseDB

MODULE 0: CLINICAL PURPOSE AND USE CONTEXT

Model name / version: Self-Attention ResUNet
Estimation family: [ ] Hemodynamics-derived [x] Data-driven BP regression [ ] ABP waveform reconstruction
Calibration paradigm: [x] Calibration-free [ ] Calibration-based
Model type: [ ] Machine learning (ML) [x] Deep learning (DL) [ ] Hybrid [ ] Other
Input data type: [ ] Handcrafted features [x] Time series [ ] Images [ ] [ ] Demographic [ ] Other
Output variable: [x] SBP [x] DBP [ ] MAP
Signal modality: [x] PPG [x] ECG [ ] BCG [ ] BioZ [ ] IPG [ ] iPPG [ ] Other
Sensor specification: Sampling rate 125 Hz
Sensor location: PPG fingertip sensor, Chest ECG (Lead-II)
Intended use: [x] Research use only [ ] Home / ambulatory monitoring [ ] Adjunctive clinical monitoring [ ] Clinical pilot / prospective evaluation

MODULE 1: DATASET AND COHORT REPORTING

Dataset source: [ ] Private [x] Public: PulseDB (based on MIMIC-III and VitalDB)
Participants (number of subjects): 5,361
Recording sessions: NA **Total paired BP samples:** NA

Population type: [ ] Healthy [ ] Hypertensive [x] ICU [x] Other: Surgical
Age (years): range 17-92
Sex: NA
BMI (kg/m²): NA
Height (cm): NA
Weight (kg): NA
Ethnicity / geographic region: South Korea (VitalDB)
Medical history / comorbidities reported: [x] Yes [ ] No
Medication reported: [ ] Yes [x] No;
Known sensitivity: [ ] Motion [ ] Low perfusion [ ] Arrhythmia [ ] Sensor displacement [ ] Skin tone [ ] Other
Skin tone / Fitzpatrick: NA (if PPG-based)
Ambient light conditions: NA (if PPG-based)

Measurement phases (%):
Ambulatory: 0 | In-clinic: 100 | Dynamic: 0| Long-term: 0| Sleep: 0
BP profiles (%):
Hypotension: NA | Normal: NA | Elevated: NA | Stage 1: NA | Stage 2: NA
SBP (mmHg): NA
DBP (mmHg): NA
Baseline SBP: NA
Baseline DBP: NA
ΔSBP: NA [ ] Subject-specific [ ] Population-based
ΔDBP: NA [ ] Subject-specific [ ] Population-based

Preprocessing pipeline: 10-s windows. ECG + PPG synchronized segments; outlier filtering
Signal quality filtering / exclusion criteria: subjects younger than 17 years; physiological implausible BP values
Fiducial points / feature extraction: No handcrafted features
Excluded samples after cleaning: NA
BP distribution before/after cleaning reported: [ ] Yes [x] No

BP distribution:

Not provided

MODULE 2. VALIDATION STRATEGY

Evaluation method: [x] Subject-wise hold-out [ ] Subject-wise CV [ ] LOSO / LOOCV [ ] Record-wise split [ ] Sample-wise split
Data split details: Intra-dataset hold-out; test designed according to AAMI.
Unseen-subject test set: [x] Yes [ ] No
External validation (independent cohort / site): [x] Yes [ ] No
Reference standard: intermittent noninvasive BP measurements
Reference device (manufacturer/model): NA

Measurement conditions: [ ] Resting [ ] Ambulatory [ ] Dynamic [ ] Long-term [x] ICU monitoring [x] Other: under anesthesia during surgery
Body position: supine or semi-recumbent ICU bed position
Activity state: sedated / resting / low-mobility
Rest period prior to measurement: NA
Baseline comparator: NA
Comparator result: Self-Attention ResUNet

Minimum acceptable standard met: partially pass AAMI and IEEE [x] Subject-wise validation

MODULE 3. PERFORMANCE REPORTING

Models evaluated: [ ] Baseline model [x] Proposed / final model

Performance reporting table (test-subset-3 train on MIMIC-III):

SBP/Group	ΔBP±SD (mmHg)	MD±SD (mmHg)	MAD (mmHg)	MAPD (%)	CP5 (%)	CP10 (%)	CP15 (%)	R
All	-	0.27±11.83	6.59	-	-	-	-	0.38
Normal	-	-	-	-	-	-	-	-
Prehypertension	-	-	-	-	-	-	-	-
HTN Stage 1	-	-	-	-	-	-	-	-
HTN Stage 2	-	-	-	-	-	-	-	-
**DBP/Group**	**ΔBP±SD (mmHg)**	**MD±SD (mmHg)**	**MAD (mmHg)**	**MAPD (%)**	**CP5 (%)**	**CP10 (%)**	**CP15 (%)**	**R**
All	-	0.21±9.37	6.46	-	-	-	-	0.72
Normal	-	-	-	-	-	-	-	-
Prehypertension	-	-	-	-	-	-	-	-
HTN Stage 1	-	-	-	-	-	-	-	-
HTN Stage 2	-	-	-	-	-	-	-	-

Performance visualization:

(b) Scatter plot **(c) Bland-Altman plot**

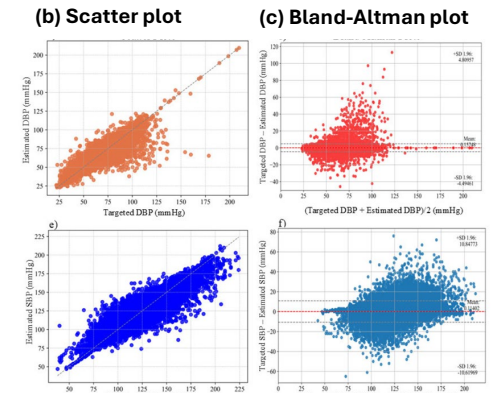

Supplementary Figure 5: Calibration-free model card corresponding to the study by Jamil et al. (2025): Blood Pressure Estimation Using Self-Attention Mechanism Built-In ResUNet on PulseDB: Demographic Fairness and Generalization.

(Baek et al., 2024): Cufless BP estimation model using clustering techniques

MODULE 0: CLINICAL PURPOSE AND USE CONTEXT

Model name / version: Clustered ANN + RNN BP estimation framework
Estimation family: [ ] Hemodynamics-derived [x] Data-driven BP regression [ ] ABP waveform reconstruction
Calibration paradigm: [ ] Calibration-free [x] Calibration-based
Model type: [ ] Machine learning (ML) [ ] Deep learning (DL) [ ] Hybrid [ ] Other
Input data type: [x] Handcrafted features [ ] Time series [ ] Images [ ] Demographic [ ] Other
Output variable: [x] SBP [x] DBP [ ] MAP
Signal modality: [x] PPG [x] ECG [ ] BCG [ ] BioZ [ ] IPG [ ] iPPG [ ] Multimodal [ ] Other
Sensor specification: NA
Sensor location: Chest ECG (lead II), finger PPG
Intended use: [x] Research use only [ ] Home / ambulatory monitoring [ ] Adjunctive clinical monitoring [ ] Clinical pilot / prospective evaluation

MODULE 1: DATASET AND COHORT REPORTING

Dataset source: [ ] Private [x] Public: VIitalDB and MIMIC-III subset
Participants (number of subjects): 6388 (VitalDB)
Recording sessions: 236 (MIMIC subset) **Total paired BP samples:** NA

Population type: [ ] Healthy [ ] Hypertensive [x] ICU [ ] Other
Age (years): 53 ± 12; range [8-92]
Sex: % female 46; % male 54
BMI (kg/m²): 22 ±4
Height (cm): 165 ± 8
Weight (kg): 62 ±10
Ethnicity / geographic region: NA
Medical history / comorbidities reported: [ ] Yes [x] No
Medication reported: [ ] Yes [x] No;
Known sensitivity: [ ] Motion [ ] Low perfusion [ ] Arrhythmia [ ] Sensor displacement [ ] Skin tone [ ] Other
Skin tone / Fitzpatrick: NA (if PPG-based)
Ambient light conditions: NA (if PPG-based)

Measurement phases (%):
Ambulatory: 0 | In-clinic: 100 | Dynamic: 0 | Long-term: 0 |Sleep: 0
BP profiles (%):
Hypotension: NA | Normal: NA | Elevated: NA | Stage 1: NA | Stage 2: NA
SBP (mmHg): 112 ± 14
DBP (mmHg): 65 ± 10
Baseline SBP (mmHg): NA
Baseline DBP (mmHg): NA
ΔSBP (mmHg): 34 ± 21 [x] Subject-specific [ ] Population-based
ΔDBP (mmHg): 21 ± 13 [x] Subject-specific [ ] Population-based

Preprocessing pipeline: ECG and PPG bandpass filter; ensemble averaging of 10 cardiac cycles; artifact rejection; feature extraction; calibration normalization; cubic spline interpolation; frequency decomposition
Signal quality filtering / exclusion criteria: missing ECG/PPG/ABP; distorted signals; insufficient clean cardiac cycle; SBP < 50 or > 250 mmHg; DBP < 30 or > 160 mmHg; BP changes > 30 mmHg within 5 s
Fiducial points / feature extraction: 29 handcrafted ECG/PPG features
Excluded samples after cleaning: VitalDB1675 (patients) / 1,051,462 cardiac cycles
BP distribution before/after cleaning reported: [ ] Yes [x] No

BP distribution :
(a) BP distribution:

Not provided

(b) Calibration-relative BP distribution:

Not provided

MODULE 2. VALIDATION STRATEGY

Evaluation method: [ ] Subject-wise hold-out [ ] Subject-wise CV [ ] LOSO / LOOCV [ ] Record-wise split [x] Sample-wise split
Data split details: random hold-out split 80%/20% (train/test)
Unseen-subject test set: [x] Yes [ ] No
External validation (independent cohort): [x] Yes (MIMIC-III) [ ] No
Reference standard: Invasive ABP reference
Reference device (manufacturer/model): NA

Measurement conditions: [ ] Resting [ ] Ambulatory [ ] Dynamic [ ] Long-term [x] ICU monitoring [ ] Other
Body position: supine or semi-recumbent ICU bed position
Activity state: sedated / perioperative monitoring
Rest period prior to measurement: Not reported
Baseline comparator: NA
Comparator result: clustered ANN + RNN framework

Minimum acceptable standard met: AAMI, Grade A BHS, IEEE [ ] Subject-wise validation

MODULE 3. PERFORMANCE REPORTING

Models evaluated: [] Baseline model [x] Proposed / final model

Calibration condition: Subject-specific offset calibration
Calibration type: [ ] One-point [ ] Two-point [ ] Multi-point [x] Other: Subject mean normalization / subject-offset calibration
Static test: [x] Yes [ ] No **Dynamic test:** [ ] Yes [x] No
Time since calibration evaluated: [ ] Yes [x] No; if yes: **Time interval** (days): -
Recalibration trigger (if any): NA

Performance reporting table:

SBP Condition	ΔBP±SD (mmHg)	MD±SD (mmHg)	MAD (mmHg)	MAPD (%)	CP5 (%)	CP10 (%)	CP15 (%)	R
Static	34± 21	-0.006±7.01	5.25	-	61	88	97	-
Dynamic	-	-	-	-	-	-	-	-
Time after calibration	-	-	-	-	-	-	-	-

DBP Condition	ΔBP±SD (mmHg)	MD±SD (mmHg)	MAD (mmHg)	MAPD (%)	CP5 (%)	CP10 (%)	CP15 (%)	R
Static	21± 13	0.051±4.84	4.47	-	82	93	98	
Dynamic	-	-	-	-	-	-	-	-
Time after calibration	-	-	-	-	-	-	-	-

Performance visualization:

(e) Scatter plot

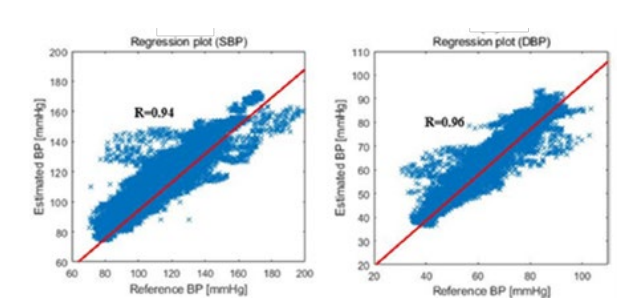

(f) Bland-Altman plot

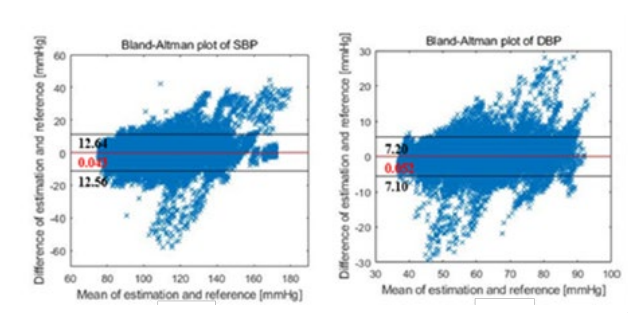

Supplementary Figure 6: Calibration-based model card corresponding to the study by Baek et al. (2024): Cuffless Blood Pressure Estimation Model Using Clustering Techniques.

(Song et al., 2020): Cufless Deep Learning-based BP Estimation

MODULE 0: CLINICAL PURPOSE AND USE CONTEXT

Model name / version: Two stage stacked DNN BP estimation framework
Estimation family: [ ] Hemodynamics-derived [x] Data-driven BP regression [ ] ABP waveform reconstruction
Calibration paradigm: [ ] Calibration-free [x] Calibration-based
Model type: [ ] Machine learning (ML) [x] Deep learning (DL) [ ] Hybrid [ ] Other
Input data type: [x] Handcrafted features [ ] Time series [ ] Images [x] Demographic [ ] Other
Output variable: [x] SBP [x] DBP [ ] MAP
Signal modality: [x] PPG [x] ECG [ ] BCG [ ] BioZ [ ] IPG [ ] iPPG [ ] Other
Sensor specification: InBody smartwatch (ECG sensor AD8233, PPG sensor ADPD174GGI, sample rate 500 Hz)
Sensor location: Wrist-worn smartwatch
Intended use: [x] Research use only [x] Home / ambulatory monitoring [] Adjunctive clinical monitoring [ ] Clinical pilot / prospective evaluation

MODULE 1: DATASET AND COHORT REPORTING

Dataset source: [x] Private [ ] Public
Participants (number of subjects): 110
Recording sessions: 3 times per subject **Total paired BP samples:** 20 s recording

Population type: [x] Healthy [ ] Hypertensive [ ] ICU [ ] Other
Age (years): 36.2 ± 10.0 (train); 37.1 ± 11.6 (test)
Sex: 37.9 % male (train); 21.2 % male (test);
BMI (kg/m^2): 23.8 ± 3.5 (train)/ 22.9 ± 3.0 (test)
Height (cm): NA
Weight (kg): NA
Ethnicity / geographic region: NA
Medical history / comorbidities reported: [ ] Yes [x] No
Medication reported: [ ] Yes [x] No
Known sensitivity: [ ] Motion [ ] Low perfusion [ ] Arrhythmia [ ] Sensor displacement [ ] Skin tone [ ] Other
Skin tone / Fitzpatrick: NA (if PPG-based)
Ambient light conditions: NA (if PPG-based)

Measurement phases (%):
Ambulatory: 0| In-clinic: 85 | Dynamic: 0 | Long-term: 15 |Sleep: 0
BP profiles (n):
Train: Hypotension: 0 | Normal: 233 | Elevated: 53 | Stage 1: 19| Stage 2: 0
Test: Hypotension: 0 | Normal: 140| Elevated: 47 | Stage 1: 3| Stage 2: 0
SBP (mmHg): 106.8 ± 15.2 (train)/ 107.8 ± 10.3 (test)
DBP (mmHg): 67.6 ± 11.9 (train)/68.1 ± 9.4 (test)
Baseline SBP: NA
Baseline DBP: NA
ΔSBP: NA
ΔDBP: NA

Preprocessing pipeline: low-pass filter for PPG and ECG; peak/valley detection; feature extraction; median filtering; feature selection; normalization
Signal quality filtering / exclusion criteria: NA
Fiducial points / feature extraction: 12 handcrafted features
Excluded samples after cleaning: NA
BP distribution before/after cleaning reported: [ ] Yes [x] No

BP distribution:

(a) BP distribution histogram

Not provided

(b) Calibration-relative BP distribution

Not provided

MODULE 2. VALIDATION STRATEGY

Evaluation method: [x] Subject-wise hold-out [ ] Subject-wise CV [ ] LOSO / LOOCV [ ] Record-wise split [ ] Sample-wise split
Data split details: 6/4 (train/test)
Unseen-subject test set: [x] Yes [ ] No
External validation (independent cohort / site): [ ] Yes [x] No
Reference standard: mercury sphygmomanometer
Reference device (manufacturer/model): Desk type 0320 Baumanometer

Measurement conditions: [x] Resting [ ] Ambulatory [ ] Dynamic [ ] Long-term [ ] ICU monitoring [ ] Other
Body position: Sitting
Activity state: Resting state
Rest period prior to measurement: 4 minutes
Baseline comparator: NA
Comparator result: Stacked DNN

Minimum acceptable standard met: AAMI, IEEE and grade A BHS [x] Subject-wise validation

MODULE 3. PERFORMANCE REPORTING

Models evaluated: [ ] Baseline model [x] Proposed / final model

Calibration condition: Resting
Calibration type: [ ] One-point [ ] Two-point [ ] Multi-point [x] Other: Subject-specific personal adaptation / fine-tuning using repeated cuff BP measurements (7-17 days)
Static test: [x] Yes [ ] No **Dynamic test:** [x] Yes [] No (after drinking coffee)
Time since calibration evaluated: [] Yes [x] No
Recalibration trigger (if any): -

Performance reporting table:

SBP / Condition	ΔBP±SD (mmHg)	MD±SD (mmHg)	MAD (mmHg)	MAPD (%)	CP5 (%)	CP10 (%)	CP15 (%)	R
Static	-	-2.8±4.9	4.2	-	-	-	-	-
Dynamic	-	-10.5±5.0	14.3	-	-	-	-	-
Time after calibration	-	-	-	-	-	-	-	-

DBP / Condition	ΔBP±SD (mmHg)	MD±SD (mmHg)	MAD (mmHg)	MAPD (%)	CP5 (%)	CP10 (%)	CP15 (%)	R
Static	-	-0.7±4.0	2.8	-	-	-	-	-
Dynamic	-	14.3±10.8	11.0	-	-	-	-	
Time after calibration				-	-	-	-	-

Visualization formats:

(e) Scatter plot

Not provided

(f) Bland-Altman plot

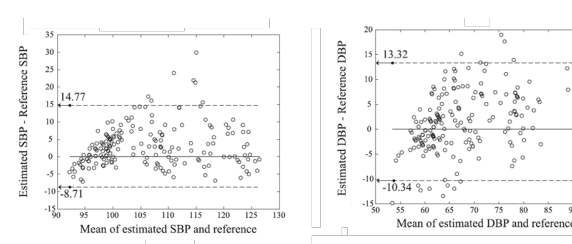

Supplementary Figure 7: Calibration-free model card corresponding to the study by Song et al. (2020): Cuffless Deep Learning-Based BP Estimation for Smart Wristwatches.

(Ma et al., 2025): DiffCNBP: Lightweight Diffusion Model

MODULE 0: CLINICAL PURPOSE AND USE CONTEXT

Model name / version: DiffCNBP
Estimation family: [ ] Hemodynamics-derived [ ] Data-driven BP regression [x] ABP waveform reconstruction
Calibration paradigm: [x] Calibration-free [ ] Calibration-based
Model type: [ ] Machine learning (ML) [x] Deep learning (DL) [ ] Hybrid [ ] Other
Input data type: [ ] Handcrafted features [x] Time series [ ] Images [ ] Demographic [ ] Other
Output variable: [x] SBP [x] DBP [x] MAP
Signal modality: [x] PPG [ ] ECG [ ] BCG [ ] BioZ [ ] IPG [ ] iPPG [ ] Other
Sensor specification: NA
Sensor location: Finger PPG
Intended use: [x] Research use only [x] Home / ambulatory monitoring [ ] Adjunctive clinical monitoring [ ] Clinical pilot / prospective evaluation

MODULE 1: DATASET AND COHORT REPORTING

Dataset source: [ ] Private [x] Public: Mindray and MIMIC-III
Participants (number of subjects): 701 Mindray; 815 MIMIC)
Recording sessions: NA **Total paired BP samples:** Mindray: 600,185; MIMIC: 711,258

Population type: [ ] Healthy [ ] Hypertensive [x] ICU [x] Other: surgical
Age (years): 60.6 ± 14.7 (Mindray); 57.3 ± 16.3 (MIMIC);
Sex: 76.1 % male (Mindray); 45.8% male (MIMIC);
BMI (kg/m²): NA
Height (cm): NA
Weight (kg): NA
Ethnicity / geographic region: NA
Medical history / comorbidities reported: [ ] Yes [x] No
Medication reported: [ ] Yes [x] No;
Known sensitivity: [ ] Motion [ ] Low perfusion [ ] Arrhythmia [ ] Sensor displacement [ ] Skin tone [ ] Other
Skin tone / Fitzpatrick: NA (if PPG-based)
Ambient light conditions: NA (if PPG-based)

Measurement phases (%):
Ambulatory: 0| In-clinic: 100 | Dynamic: 0| Long-term: 0 |Sleep: 0
BP profiles (%):
Hypotension NA | Normal NA | Elevated NA | Stage 1 NA | Stage 2 NA
SBP (mmHg): 131.25 ± 26.30 (Mindray); 125.38 ± 24.77 (MIMIC)
DBP (mmHg): 73.28± 21.12 (Mindray); 73.65± 22.15 (MIMIC)
Baseline SBP (mmHg): NA
Baseline DBP (mmHg): NA
ΔSBP (mmHg): NA
ΔDBP (mmHg): NA

Preprocessing pipeline: wavelet denoising; baseline drift removal; phase alignment; signal normalization; outlier removal; sliding-window segmentation; beat alignment
Signal quality filtering / exclusion criteria: distorted PPG; horizontal BP lines; excessive motion artifacts; BP < 20 mmHg; low-correlation segments; abnormal waveform morphology
Excluded samples after cleaning: 1415 subjects (683 Mindray; 732 MIMIC); 1,255,663 samples (Mindray: 581,293; MIMIC: 674,370)
BP distribution before/after cleaning reported: [ ] Yes [x] No

(a) BP distribution histogram:

Not reported

MODULE 2. VALIDATION STRATEGY

Evaluation method: [ ] Subject-wise hold-out [x] Subject-wise CV [ ] LOSO / LOOCV [ ] Record-wise split [ ] Sample-wise split
Data split details: subject-wise 5-fold cross-validation
Unseen-subject test set: [x] Yes [ ] No
External validation (independent cohort / site): [x] Yes [ ] No (MIMIC)
Reference standard: Invasive arterial blood pressure waveform
Reference device (manufacturer/model): ICU bedside model

Measurement conditions: [ ] Resting [ ] Ambulatory [ ] Dynamic [ ] Long-term [x] ICU monitoring [x] Other: under anesthesia
Body position: supine or semi-recumbent ICU bed position
Activity state: resting/low-mobility ICU monitoring
Rest period prior to measurement: NA
Baseline comparator: NA
Comparator result: DiffCNBP

Minimum acceptable standard met: pass AAMI, IEEE, Grade A BHS [x] Subject-wise validation

MODULE 3. PERFORMANCE REPORTING

Models evaluated: [] Baseline model [x] Proposed / final model

Performance reporting table:

SBP/Group	ΔBP±SD (mmHg)	MD±SD (mmHg)	MAD (mmHg)	MAPD (%)	CP5 (%)	CP10 (%)	CP15 (%)	R
**All**	-	0.67±4.29	2.98	-	92.48	87.20	91.00	0.97
**Normal**	-	-	-	-	-	-	-	-
**Prehypertension**	-	-	-	-	-	-	-	-
**HTN Stage 1**	-	-	-	-	-	-	-	-
**HTN Stage 2**	-	-	-	-	-	-	-	-

DBP/Group	ΔBP±SD (mmHg)	MD±SD (mmHg)	MAD (mmHg)	MAPD (%)	CP5 (%)	CP10 (%)	CP15 (%)	R
**All**	-	0.37±2.49	1.71	-	90.35	97.30	98.94	0.95
**Normal**	-	-	-	-	-	-	-	-
**Prehypertension**	-	-	-	-	-	-	-	-
**HTN Stage 1**	-	-	-	-	-	-	-	-
**HTN Stage 2**	-	-	-	-	-	-	-	-

Performance visualization:

(b) Scatter plot

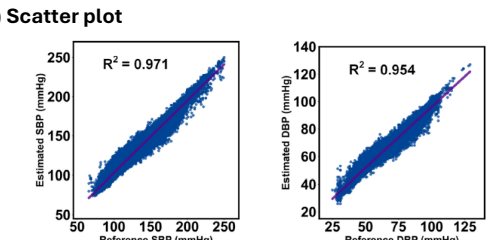

(c) Bland-Altman plot

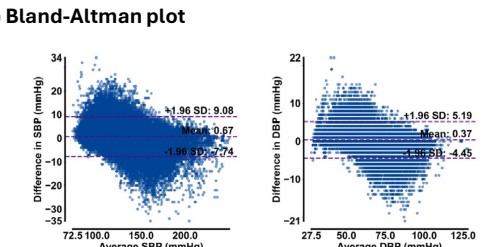

Supplementary Figure 8: Calibration-free model card corresponding to the study by Ma et al. (2025): DiffCNBP: Lightweight Diffusion Model for IoMT-Based Continous Cuffless Blood Pressure Waveform Monitoring using PPG.

(Baek et al., 2019): End-to-End BP Prediction via Fully Convolutional Networks

MODULE 0: CLINICAL PURPOSE AND USE CONTEXT

Model name / version: End-to-End Blood Pressure Prediction via Fully Convolutional Networks
Estimation family: [ ] Hemodynamics-derived [x] Data-driven BP regression [ ] ABP waveform reconstruction
Calibration paradigm: [ ] Calibration-free [x] Calibration-based
Model type: [ ] Machine learning (ML) [x] Deep learning (DL) [ ] Hybrid [ ] Other
Input data type: [ ] Handcrafted features [x] Time series [ ] Images [ ] Demographic [ ] Other
Output variable: [x] SBP [x] DBP [ ] MAP
Signal modality: [x] PPG [x] ECG [ ] BCG [ ] BioZ [ ] IPG [ ] iPPG [ ] Other
Sensor specification: NA
Sensor location: ECG chest (lead II); fingertip PPG
Intended use: [x] Research use only [ ] Home / ambulatory monitoring [ ] Adjunctive clinical monitoring [ ] Clinical pilot / prospective evaluation

MODULE 1: DATASET AND COHORT REPORTING

Dataset source: [ ] Private [x] Public: MIMIC II
Participants (number of subjects): 942 (final)
Recording sessions: NA **Total paired BP samples:** NA

Population type: [ ] Healthy [ ] Hypertensive [x] ICU [ ] Other
Age (years): NA
Sex: NA
BMI (kg/m²): NA
Height (cm): NA
Weight (kg): NA
Ethnicity / geographic region: NA
Medical history / comorbidities reported: [ ] Yes [x] No
Medication reported: [ ] Yes [x] No
Known sensitivity: [ ] Motion [ ] Low perfusion [ ] Arrhythmia [ ] Sensor displacement [ ] Skin tone [ ] Other
Skin tone / Fitzpatrick: NA (if PPG-based)
Ambient light conditions: NA (if PPG-based)

Measurement phases (%):
Ambulatory: 0 | In-clinic: 100 | Dynamic: 0 | Long-term: 0 |Sleep 0:
BP profiles (%):
Hypotension: NA | Normal: NA | Elevated: NA | Stage 1: NA | Stage 2: NA
SBP (mmHg): NA
DBP (mmHg): NA
Baseline SBP (mmHg): NA
Baseline DBP (mmHg): NA
ΔSBP (mmHg): NA [ ] Subject-specific [ ] Population-based
ΔDBP (mmHg): NA [ ] Subject-specific [ ] Population-based

Preprocessing pipeline: Random segment sampling; random cropping; FFT transformation; concatenation of first and second signal derivatives
Signal quality filtering / exclusion criteria: SBP range: 90-180 mmHg; DBP range: 60-120 mmHg; peak consistency filtering based on peak count and variance
Fiducial points / feature extraction: No handcrafted fiducial features;
Excluded samples after cleaning: 12,000 records (initial)/1,912 (final)
BP distribution before/after cleaning reported: [ ] Yes [x] No

BP distribution:
(a) BP distribution histogram

Not reported

(b) Calibration-relative BP distribution

Not reported

MODULE 2. VALIDATION STRATEGY

Evaluation method: [x] Subject-wise hold-out [ ] Subject-wise CV [ ] LOSO / LOOCV [ ] Record-wise split [ ] Sample-wise split
Data split details: 70% training / 10% validation / 20% testing
Unseen-subject test set: [x] Yes [ ] No
External validation (independent cohort / site): [ ] Yes [x] No
Reference standard: Invasive arterial blood pressure (ABP) waveform
Reference device (manufacturer/model): MIMIC II ICU monitoring system (specific device not reported)

Measurement conditions: [ ] Resting [ ] Ambulatory [ ] Dynamic [ ] Long-term [x] ICU monitoring [ ] Other
Body position: supine
Activity state: ICU resting/monitoring conditions
Rest period prior to measurement: NA
Baseline comparator: NA
Comparator result: End-to-end CNN

Minimum acceptable standard met: AAMI pass; BHS: SBP grade B and DBP grade A [x] Subject-wise validation

MODULE 3. PERFORMANCE REPORTING

Models evaluated: [] Baseline model [x] Proposed / final model

Calibration condition: Personalized recalibration using partial subject data from the test set
Calibration type: [ ] One-point [ ] Two-point [x] Multi-point [ ] Other
Static test: [x] Yes [ ] No **Dynamic test:** [ ] Yes [x] No
Time since calibration evaluated: [ ] Yes [x] No
Recalibration trigger (if any): NA

Performance reporting table:

SBP/ Condition	ΔBP±SD (mmHg)	MD±SD (mmHg)	MAD (mmHg)	MAPD (%)	CP5 (%)	CP10 (%)	CP15 (%)	R
**Static**	-	-1.29±7.58	5.32	NR	64.7	89.4	96.8	0.89
**Dynamic**	-	-	-	-	-	-	-	-
**Time after calibration**	-	-	-	-	-	-	-	-

DBP/ Condition	ΔBP±SD (mmHg)	MD±SD (mmHg)	MAD (mmHg)	MAPD (%)	CP5 (%)	CP10 (%)	CP15 (%)	R
**Static**	-	-0.48±5.08	3.38	-	80.5	96.0	98.9	0.8
**Dynamic**	-	-	-	-	-	-	-	-
**Time after calibration**	-	-	-	-	-	-	-	-

Visualization formats:

(e) Scatter plot

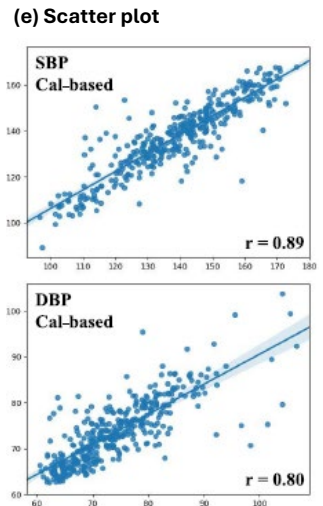

(f) Bland-Altman plot

Not reported

Supplementary Figure 9: Calibration-based model card corresponding to the study by Baek et al. (2019): End-to-End BP Prediction via Fully Convolutional Networks.

(Rizal et al., 2024): GRU-Based Fusion Models for Enhanced BP Estimation From PPG Signals

MODULE 0: CLINICAL PURPOSE AND USE CONTEXT

Model name / version: Dual GRU + ANN fusion model
Estimation family: [ ] Hemodynamics-derived [ ] Data-driven BP regression [ ] ABP waveform reconstruction
Calibration paradigm: [x] Calibration-free [ ] Calibration-based
Model type: [ ] Machine learning (ML) [ ] Deep learning (DL) [ ] Hybrid [ ] Other
Input data type: [ ] Handcrafted features [x] Time series [ ] Images [x] Other: Demographic
Output variable: [ ] SBP [ ] DBP [ ] MAP
Signal modality: [x] PPG [ ] ECG [ ] BCG [ ] BioZ [ ] IPG [ ] iPPG [ ] Other
Sensor specification: Custom portable PPG hardware platform
Sensor location: Fingertip PPG
Intended use: [x] Research use only [x] Home / ambulatory monitoring [ ] Adjunctive clinical monitoring [ ] Clinical pilot / prospective evaluation

MODULE 1: DATASET AND COHORT REPORTING

Dataset source: [ ] Private [x] Public: PPGBP and MIMIC III subset
Participants (number of subjects): 244 (PPGBP: 219; MIMIC III: 25)
Recording sessions: NA **Total paired BP samples:** PPGBP: 657

Population type: [x] Healthy (PPGBP) [ ] Hypertensive [X] ICU (MIMIC) [ ] Other
Age (years): NA
Sex: NA
BMI (kg/m²): NA
Height (cm): NA
Weight (kg): NA
Ethnicity / geographic region: NA
Medical history / comorbidities reported: [x] Yes: hypertension and diabetes (PPGBP) [] No
Medication reported: [ ] Yes [x] No
Known sensitivity: [ ] Motion [ ] Low perfusion [ ] Arrhythmia [ ] Sensor displacement [ ] Skin tone [ ] Other
Skin tone / Fitzpatrick: NA (if PPG-based)
Ambient light conditions: NA (if PPG-based)

Measurement phases (%):
Ambulatory: 0 | In-clinic: 100 | Dynamic: 0 | Long-term: 0 |Sleep: 0
BP profiles (%):
Hypotension NA | Normal NA | Elevated NA | Stage 1 NA | Stage 2 NA
SBP (mmHg): NA
DBP (mmHg): NA
Baseline SBP: NA
Baseline DBP: NA
ΔSBP: NA [ ] Subject-specific [ ] Population-based
ΔDBP: NA [ ] Subject-specific [ ] Population-based

Preprocessing pipeline: Butterworth bandpass filter; high-pass and low-pass filter; signal normalization; noise/artifact reduction
Signal quality filtering / exclusion criteria: minimal noise/artifacts; complete heartbeat cycles required
Fiducial points / feature extraction: No handcrafted features
Excluded samples after cleaning: NA
BP distribution before/after cleaning reported: [x] Yes [ ] No

BP distribution :

Not provided

MODULE 2. VALIDATION STRATEGY

Evaluation method: [x] Subject-wise hold-out [ ] Subject-wise CV [ ] LOSO / LOOCV [ ] Record-wise split [ ] Sample-wise split
Data split details: 60/25/15 (train/validation/test – PPGBP)
Unseen-subject test set: [x] Yes [ ] No
External validation (independent cohort / site): [x] Yes [ ] No (MIMIC)
Reference standard: Custom acquisition platform with simultaneous arterial BP recording.
Reference device (manufacturer/model): Not specified

Measurement conditions: [x] Resting (PPGBP) [ ] Ambulatory [ ] Dynamic [ ] Long-term [x] ICU monitoring (MIMIC) [ ] Other
Body position: NA (PPGBP) / supine or semi-recumbent (MIMIC)
Activity state: resting (PPGBP) / resting/low-mobility ICU monitoring (MIMIC)
Rest period prior to measurement: NA
Baseline comparator: NA
Comparator result: Dual GRU + ANN fusion model

Minimum acceptable standard met: IEEE Grade A, pass AAMI, Grade A BHS [x] Subject-wise validation

MODULE 3. PERFORMANCE REPORTING

Models evaluated: [] Baseline model [x] Proposed / final model

Performance reporting table:

SBP / Group	ΔBP±SD (mmHg)	MD±SD (mmHg)	MAD (mmHg)	MAPD (%)	CP5 (%)	CP10 (%)	CP15 (%)	R
**All**	-	0.84±2.66	1.459	1.114	96.36	96.36	100.0	-
**Normal**	-	-	-	-	-	-	-	-
**Prehypertension**	-	-	-	-	-	-	-	-
**HTN Stage 1**	-	-	-	-	-	-	-	-
**HTN Stage 2**	-	-	-	-	-	-	-	-

DBP / Group	ΔBP±SD (mmHg)	MD±SD (mmHg)	MAD (mmHg)	MAPD (%)	CP5 (%)	CP10 (%)	CP15 (%)	R
**All**	-	0.04±1.63	1.165	1.687	98.78	100.0	100.0	-
**Normal**	-	-	-	-	-	-	-	-
**Prehypertension**	-	-	-	-	-	-	-	-
**HTN Stage 1**	-	-	-	-	-	-	-	-
**HTN Stage 2**	-	-	-	-	-	-	-	-

Visualization formats:
(b) Scatter plot

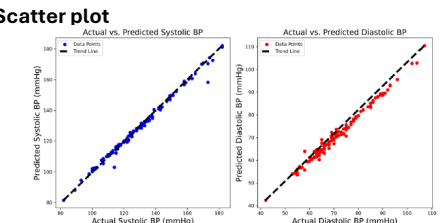

(c) Blan-Altman plot

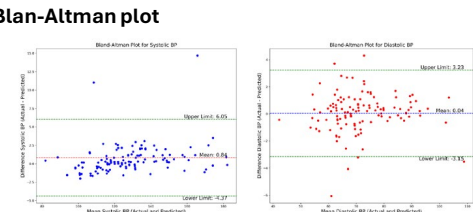

Supplementary Figure 10: Calibration-free model card corresponding to the study by Rizal et al. (2024): GRU-Based Fusion Models for Enhanced BP Estimation From PPG Signals.

(Ma et al., 2023): KD-Infomer: Cuff-Less Continuous BP Waveform Estimation Approach based on Single PPG

MODULE 0: CLINICAL PURPOSE AND USE CONTEXT

Model name / version: KD-Informer
Estimation family: [ ] Hemodynamics-derived [ ] Data-driven BP regression [x] ABP waveform reconstruction
Calibration paradigm: [x] Calibration-free [ ] Calibration-based
Model type: [ ] Machine learning (ML) [x] Deep learning (DL) [ ] Hybrid [ ] Other
Input data type: [x] Handcrafted features [x] Time series [ ] Images [ ] Demographic [ ] Other
Output variable: [x] SBP [x] DBP [ ] MAP
Signal modality: [x] PPG [ ] ECG [ ] BCG [ ] BioZ [ ] IPG [ ] iPPG [ ] Other
Sensor specification: NA
Sensor location: NA
Intended use: [x] Research use only [ ] Home / ambulatory monitoring [ ] Adjunctive clinical monitoring [ ] Clinical pilot / prospective evaluation

MODULE 1: DATASET AND COHORT REPORTING

Dataset source: [x] Private: Mindray [x] Public: MIMIC
Participants (number of subjects): 467 (Mindray); NA (MIMIC)
Recording sessions: NA **Total paired BP samples:** NA

Population type: [ ] Healthy [ ] Hypertensive [x] ICU (MIMIC) [x] Other: Multicenter clinical population (Mindray)
Age (years): NA
Sex: NA
BMI (kg/m^2): NA
Height (cm): NA
Weight (kg): NA
Ethnicity / geographic region: NA
Medical history / comorbidities reported: [ ] Yes [X] No
Medication reported: [ ] Yes [x] No
Known sensitivity: [ ] Motion [ ] Low perfusion [ ] Arrhythmia [ ] Sensor displacement [ ] Skin tone [ ] Other
Skin tone / Fitzpatrick: NA (if PPG-based)
Ambient light conditions: NA (if PPG-based)

Measurement phases (%):
Ambulatory: 0 | In-clinic: 100 | Dynamic: 0 | Long-term: 0 |Sleep: 0
BP profiles (%): NA
SBP (mmHg): 123.93 ± 26.40 (Mindray); 131.26 ± 30.15 (MIMCI)
DBP (mmHg): 67.70 ± 17.91 (Mindray); 69.56 ± 18.84 (MIMCI)
Baseline SBP (mmHg): NA
Baseline DBP (mmHg): NA
ΔSBP (mmHg): NA [ ] Subject-specific [ ] Population-based
ΔDBP (mmHg): NA [ ] Subject-specific [ ] Population-based

Preprocessing pipeline: Resampling; wavelet denoising; sym4 wavelet decomposition; segmentation; phase matching; z-score normalization; data augmentation; morphological feature extraction
Signal quality filtering / exclusion criteria: NA
Fiducial points / feature extraction: 75 features (32 optimal subset)
Excluded samples after cleaning: NA
BP distribution before/after cleaning reported: [ ] Yes [x] No

BP distribution :

Not provided

MODULE 2. VALIDATION STRATEGY

Evaluation method: [x] Subject-wise hold-out [ ] Subject-wise CV [ ] LOSO / LOOCV [ ] Record-wise split [ ] Sample-wise split
Data split details: 70/15/15 (train/validation/test)
Unseen-subject test set: [x] Yes [ ] No
External validation (independent cohort / site): [x] Yes [ ] No (transfer learning: pertained on Mindray, evaluated on MIMIC)
Reference standard: NA
Reference device (manufacturer/model): NA

Measurement conditions: [x] Resting [ ] Ambulatory [ ] Dynamic [ ] Long-term [x] ICU monitoring [ ] Other
Body position: Supine
Activity state: Resting
Rest period prior to measurement: NA
Baseline comparator: NA
Comparator result: KD-Infomer with morphological features on Mindray

Minimum acceptable standard met: AAMI and IEEE [x] Subject-wise validation

MODULE 3. PERFORMANCE REPORTING

Models evaluated: [] Baseline model [x] Proposed / final model

Performance reporting table (KD-Infomer with morphological features on Mindray)

SBP/Group	ΔBP±SD (mmHg)	MD±SD (mmHg)	MAD (mmHg)	MAPD (%)	CP5 (%)	CP10 (%)	CP15 (%)	R
All	-	0.013±6.237	4.178	-	-	-	-	0.90
Normal	-	-	-	-	-	-	-	-
Prehypertension	-	-	-	-	-	-	-	-
Hypertension Stage 1	-	-	-	-	-	-	-	-
Hypertension Stage 2	-	-	-	-	-	-	-	-

DBP/Group	ΔBP±SD (mmHg)	MD±SD (mmHg)	MAD (mmHg)	MAPD (%)	CP5 (%)	CP10 (%)	CP15 (%)	R
All	-	0.011±4.453	2.997	-	-	-	-	0.94
Normal	-	-	-	-	-	-	-	-
Prehypertension	-	-	-	-	-	-	-	-
Hypertension Stage 1	-	-	-	-	-	-	-	-
Hypertension Stage 2	-	-	-	-	-	-	-	-

Visualization formats:

(c) Bland-Altman plot **(b) Satter plot**

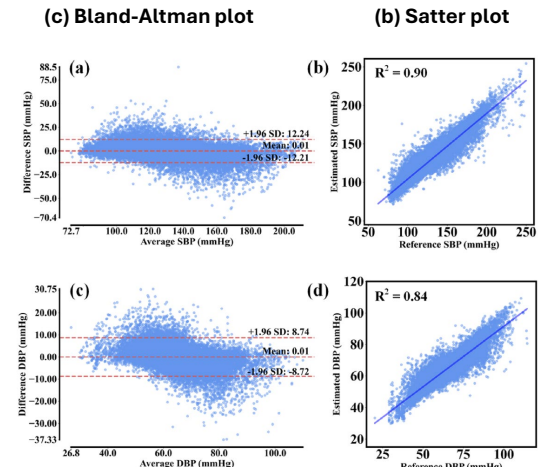

Supplementary Figure 11: Calibration-free model card corresponding to the study by Ma et al. (2023): KD-Informer:A Cuff-Less Continuous BP Waveform Estimation Approach Based on Single Photoplethysmography.

(Miao et al., 2020): Multi-sensor Fusion Approach for Cuff-less Blood Pressure Measurement

MODULE 0: CLINICAL PURPOSE AND USE CONTEXT

Model name / version: Multi-sensor fusion BP estimation model
Estimation family: [ ] Hemodynamics-derived [x] Data-driven BP regression [ ] ABP waveform reconstruction
Calibration paradigm: [ ] Calibration-free [x] Calibration-based
Model type: [x] Machine learning (ML) [ ] Deep learning (DL) [ ] Hybrid [ ] Other
Input data type: [x] Handcrafted features [ ] Time series [ ] Images [ ] [ ] Demographic [ ] Other
Output variable: [x] SBP [x] DBP [ ] MAP
Signal modality: [ ] PPG [x] ECG [ ] BCG [ ] BioZ [ ] IPG [ ] iPPG [ ] Multimodal [x] Other: PPW (pulse pressure waveform)
Sensor specification: PPW: HK-2000B
Sensor location: 2 PPW (radial and ankle artery); ECG (bilateral arm electrodes + right leg)
Intended use: [x] Research use only [ ] Home / ambulatory monitoring [ ] Adjunctive clinical monitoring [ ] Clinical pilot / prospective evaluation

MODULE 1: DATASET AND COHORT REPORTING

Dataset source: [x] Private [ ] Public
Participants (number of subjects): 85
Recording sessions: NA **Total paired BP samples:** 32

Population type: [x] Healthy [x] Hypertensive [ ] ICU [ ] Other
Age (years): 49.94 ± 9.01
Sex: 52 female; 33 male
BMI (kg/m²): NA
Height (cm): NA
Weight (kg): NA
Ethnicity / geographic region: NA
Medical history / comorbidities reported: [ ] Yes [x] No
Medication reported: [ ] Yes [x] No
Known sensitivity: [ ] Motion [ ] Low perfusion [ ] Arrhythmia [ ] Sensor displacement [ ] Skin tone [ ] Other
Skin tone / Fitzpatrick: NA (if PPG-based)
Ambient light conditions: NA (if PPG-based)

Measurement phases (%):
Ambulatory: 0| In-clinic: 100 | Dynamic: 0 | Long-term: 0 |Sleep: 0
BP profiles (n):
Hypotension : 12 | Normal: 56 | Elevated: 17 | Stage 1: 0 | Stage 2: 0
SBP (mmHg): 120.91 ± 17.84
DBP (mmHg): 72.15 ± 10.36 ;
Baseline SBP: NA
Baseline DBP: NA
ΔSBP: 26 [x] Subject-specific [ ] Population-based
ΔDBP: 18 [x] Subject-specific [ ] Population-based

Preprocessing pipeline: Band-pass filtering, waveform denoising, heartbeat segmentation
Signal quality filtering / exclusion criteria: Excessive motion artifacts, unstable physiological conditions or unreliable waveform morphonology
Fiducial points / feature extraction: Weakly supervised feature selection (WSF): 35 features (24 physiological, 11 informative)
Excluded samples after cleaning: NA
BP distribution before/after cleaning reported: [ ] Yes [x] No

BP distribution :

(a) **BP distribution histogram**

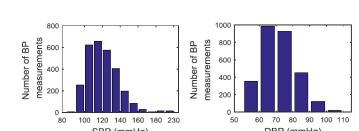

(b) Calibration-relative BP distribution

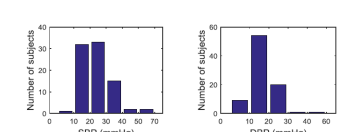

MODULE 2. VALIDATION STRATEGY

Evaluation method: [ ] Subject-wise hold-out [ ] Subject-wise CV [ ] LOSO / LOOCV [ ] Record-wise split [ ] Sample-wise split [x] Other: Subject-specific longitudinal hold-out
Data split details: For each subject: Day D: calibration/training set D+1, D+3, D+6, D+8: independent test sets D+60: long-term robustness evaluation (10 subjects)
Unseen-subject test set: [ ] Yes [x] No (models individualized per subject)
External validation (independent cohort / site): [ ] Yes [x] No
Reference standard: Oscillometric cuff BP
Reference device (manufacturer/model): OMRON HEM-7200

Measurement conditions: [x] Resting [ ] Ambulatory [ ] Dynamic [ ] Long-term [ ] ICU monitoring [ ] Other
Body position: Supine/lying
Activity state: Static resting
Rest period prior to measurement: 5 minutes
Baseline comparator: NA
Comparator result: Multi-sensor fusion BP estimation model

Minimum acceptable standard met: AAMI and grade B BHS [x] Subject-wise validation

MODULE 3. PERFORMANCE REPORTING

Models evaluated: [] Baseline model [x] Proposed / final model

Calibration condition: Subject-specific calibration using an initial per-subject training session (16 cuff-based reference measurements)
Calibration type: [ ] One-point [ ] Two-point [x] Multi-point [ ] Other
Static test: [x] Yes [ ] No **Dynamic test:** [ ] Yes [x] No
Time since calibration evaluated: [x] Yes [ ] No; if yes: **Time interval** (days): 1, 3, 6 8 and 60 days
Recalibration trigger (if any): NA

Standarized performance reporting table:

SBP/Condition	ΔBP±SD (mmHg)	MD±SD (mmHg)	MAD (mmHg)	MAPD (%)	CP5 (%)	CP10 (%)	CP15 (%)	R
Static	26	1.62±7.76	6.13	-	51	81	94	0.90
Dynamic	-	-	-	-	-	-	-	-
Time after calibration	Not numerically reported (presented graphically)							

DBP/Condition	ΔBP±SD (mmHg)	MD±SD (mmHg)	MAD (mmHg)	MAPD (%)	CP5 (%)	CP10 (%)	CP15 (%)	R
Static	18	1.49±5.52	4.54	-	62	92	99	0.84
Dynamic	-	-	-	-	-	-	-	-
Time after calibration	Not numerically reported (presented graphically)							

Visualization formats:

(e) Scatte r plot **(f) Bland-Altman plot**

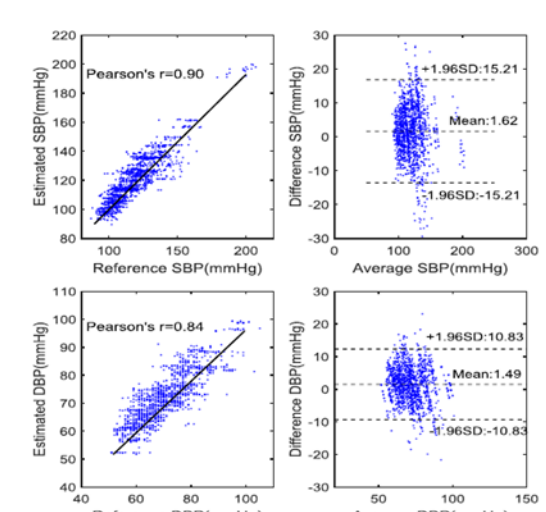

Supplementary Figure 12: Calibration-based model card corresponding to the study by Miao et al. (2020): Multi-sensor Fusion Approach for Cuff-less Blood Pressure Measurement.

(Panwar et al., 2020): PP-NET: Deep Learning Framework for PPG-based Blood Pressure and Heart Rate Estimation

MODULE 0: CLINICAL PURPOSE AND USE CONTEXT

Model name / version: PP-Net
Estimation family: [ ] Hemodynamics-derived [ ] Data-driven BP regression [x] ABP waveform reconstruction
Calibration paradigm: [x] Calibration-free [ ] Calibration-based
Model type: [ ] Machine learning (ML) [x] Deep learning (DL) [ ] Hybrid [ ] Other
Input data type: [ ] Handcrafted features [x] Time series [ ] Images [ ] Demographic [ ] Other
Output variable: [x] SBP [x] DBP [ ] MAP
Signal modality: [x] PPG [ ] ECG [ ] BCG [ ] BioZ [ ] IPG [ ] iPPG [ ] Other
Sensor specification: NA
Sensor location: NA
Intended use: [x] Research use only [ ] Home / ambulatory monitoring [ ] Adjunctive clinical monitoring [ ] Clinical pilot / prospective evaluation

MODULE 1: DATASET AND COHORT REPORTING

Dataset source: [ ] Private [x] Public: MIMIC-II/UCI
Participants (number of subjects): 12,000
Recording sessions: NA **Total paired BP samples:** NA

Population type: [ ] Healthy [ ] Hypertensive [x] ICU [ ] Other
Age (years): NA
Sex: NA
BMI (kg/m²): NA
Height (cm): NA
Weight (kg): NA
Ethnicity / geographic region: NA
Medical history / comorbidities reported: [ ] Yes [x] No
Medication reported: [ ] Yes [x No
Known sensitivity: [ ] Motion [ ] Low perfusion [ ] Arrhythmia [ ] Sensor displacement [ ] Skin tone [ ] Other
Skin tone / Fitzpatrick: NA (if PPG-based)
Ambient light conditions: NA (if PPG-based)

Measurement phases (%):
Ambulatory: 0| In-clinic: 100 | Dynamic: 0 | Long-term: 0 |Sleep: 0
BP profiles (%):
Hypotension: NA | Normal: NA | Elevated: NA | Stage 1: NA | Stage 2: NA
SBP (mmHg): 134.0 ± 23.2 ; range 80-180
DBP (mmHg): 72.5 ± 12.3 ; range 60-130
Baseline SBP: NA
Baseline DBP: NA
ΔSBP: NA [ ] Subject-specific [ ] Population-based
ΔDBP: NA [ ] Subject-specific [ ] Population-based

Preprocessing pipeline: removal recordings < 8 min; 8-s segmentation; 75% overlap; exclusion NaN values; down sampling (Fator 4); signal normalization
Signal quality filtering / exclusion criteria: SBP > 180 or < 80; DBP < 130 or < 60; HR> 220 or < 40 bpm.
Fiducial points / feature extraction: No handcrafted features
Excluded samples after cleaning: 83 % removal after duration filtering (<8 min), additional 20 % reduction after quality filtering. 1,557 subjects
BP distribution before/after cleaning reported: [ ] Yes [x] No

(a) BP distribution :

Not provided

MODULE 2. VALIDATION STRATEGY

Evaluation method: [ ] Subject-wise hold-out [x] Subject-wise CV [ ] LOSO / LOOCV [ ] Record-wise split [ ] Sample-wise split
Data split details: 10-fold CV
Unseen-subject test set: [x] Yes [ ] No
External validation (independent cohort / site): [ ] Yes [x] No
Reference standard: Invasive ABP monitoring system
Reference device (manufacturer/model): NA

Measurement conditions: [ ] Resting [ ] Ambulatory [ ] Dynamic [ ] Long-term [x] ICU monitoring [ ] Other
Body position: supine
Activity state: Static ICU monitoring condition
Rest period prior to measurement: NA
Baseline comparator: NA
Comparator result: PP-Net

Minimum acceptable standard met: Grade A BHS, pass AAMI and IEEE
[x] Subject-wise validation

MODULE 3. PERFORMANCE REPORTING

Models evaluated: [] Baseline model [x] Proposed / final model

Performance reporting table:

SBP/Group	ΔBP±SD (mmHg)	MD±SD (mmHg)	MAD (mmHg)	MAPD (%)	CP5 (%)	CP10 (%)	CP15 (%)	R
**All**	-	1.55±5.41	3.97	-	75	91	96	-
**Normal**	-	-	-	-	-	-	-	-
**Prehypertension**	-	-	-	-	-	-	-	-
**HTN Stage 1**	-	-	-	-	-	-	-	-
**HTN Stage 2**	-	-	-	-	-	-	-	-

DBP/Group	ΔBP±SD (mmHg)	MD±SD (mmHg)	MAD (mmHg)	MAPD (%)	CP5 (%)	CP10 (%)	CP15 (%)	R
**All**	-	-1.25±5.65	2.30	-	90	98	99	-
**Normal**	-	-	-	-	-	-	-	-
**Prehypertension**	-	-	-	-	-	-	-	-
**HTN Stage 1**	-	-	-	-	-	-	-	-
**HTN Stage 2**	-	-	-	-	-	-	-	-

Visualization formats

(b) Scatter plot

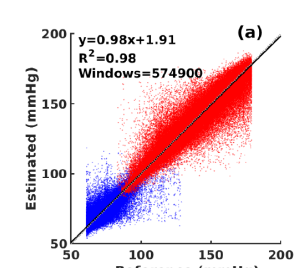

(c) Bland-Altman plot

Not provided

Supplementary Figure 13: Calibration-free model card corresponding to the study by Panwar et al. (2020): PP-NET: A Deep Learning Framework for PPG-based Blood Pressure and Heart Rate estimation.

(Ma et al., 2024): SMART-BP: SEM-ResNet and Auto-Regressor

MODULE 0: CLINICAL PURPOSE AND USE CONTEXT

Model name / version: SMART-BP
Estimation family: [ ] Hemodynamics-derived [ ] Data-driven BP regression [ ] ABP waveform reconstruction
Calibration paradigm: [x] Calibration-free [ ] Calibration-based
Model type: [ ] Machine learning (ML) [x] Deep learning (DL) [ ] Hybrid [ ] Other
Input data type: [x] Handcrafted features [x] Time series [ ] Images [] Demographic [ ] Other
Output variable: [ ] SBP [ ] DBP [ ] MAP
Signal modality: [x] PPG [ ] ECG [ ] BCG [ ] BioZ [ ] IPG [ ] iPPG [ ] Other
Sensor specification: Transmissive finger PPG sensor
Sensor location: Finger
Intended use: [x] Research use only [ ] Home / ambulatory monitoring [ ] Adjunctive clinical monitoring [ ] Clinical pilot / prospective evaluation

MODULE 1: DATASET AND COHORT REPORTING

Dataset source: [x] Private: Mindray [x] Public: MIMIC
Participants (number of subjects): Mindray: 683
Recording sessions: NA **Total paired BP samples:** MIMIC: 20,000

Population type: [ ] Healthy [ ] Hypertensive [x] ICU [ ] Other
Age (years): NA
Sex: NA
BMI (kg/m²): NA
Height (cm): NA
Weight (kg): NA
Ethnicity / geographic region: NA
Medical history / comorbidities reported: [ ] Yes [x] No
Medication reported: [ ] Yes [x] No
Known sensitivity: [ ] Motion [ ] Low perfusion [ ] Arrhythmia [ ] Sensor displacement [ ] Skin tone [ ] Other
Skin tone / Fitzpatrick: NA (if PPG-based)
Ambient light conditions: NA (if PPG-based)

Measurement phases (%):
Ambulatory: 0 | In-clinic: 100 | Dynamic: 0 | Long-term: 0 |Sleep: 0
BP profiles* (%):
Mindray: Hypotension 10.25 % | Normal 76.68% | Hypertension 13.07 %
MIMIC: Hypotension 10.03 % | Normal 71.42% | Hypertension 18.55 %
SBP (mmHg): Only reported by BP subgroup
DBP (mmHg): Only reported by BP subgroup
Baseline SBP (mmHg): NA
Baseline DBP (mmHg): NA
ΔSBP (mmHg): NA [ ] Subject-specific [ ] Population-based
ΔDBP (mmHg): NA [ ] Subject-specific [ ] Population-based

Preprocessing pipeline: Resampling to 125 Hz; sym4 wavelet filtering; decomposition level 5; soft-threshold coefficient 0.5; 8 s segment window; 3 s sliding step; cross-correlation alignment; normalization
Signal quality filtering / exclusion criteria: NA
Fiducial points / feature extraction: 75 handcrafted features
Excluded samples after cleaning: NA
BP distribution before/after cleaning reported: [ ] Yes [x] No

(a) BP distribution :

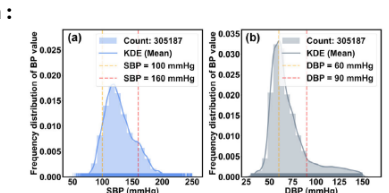

MODULE 2. VALIDATION STRATEGY

Evaluation method: [x] Subject-wise hold-out [ ] Subject-wise CV [ ] LOSO / LOOCV [ ] Record-wise split [ ] Sample-wise split
Data split details: 80/10/10 (train/vallation/test)
Unseen-subject test set: [x] Yes [ ] No
External validation (independent cohort / site): [x] Yes [ ] No (MIMIC)
Reference standard: Invasive arterial blood pressure (ABP)
Reference device (manufacturer/model): NA

Measurement conditions: [ ] Resting [ ] Ambulatory [ ] Dynamic [ ] Long-term [x] ICU monitoring [ ] Other
Body position: supine
Activity state: resting
Rest period prior to measurement: NA
Baseline comparator: NA
Comparator result: SMART-BP

Minimum acceptable standard met: AAMI and IEEE [x] Subject-wise validation

MODULE 3. PERFORMANCE REPORTING

Models evaluated: [] Baseline model [x] Proposed / final model

Performance reporting table (SMART-BP on Mindray dataset):

SBP/ Group*	ΔBP±SD (mmHg)	MD±SD (mmHg)	MAD (mmHg)	MAPD (%)	CP5 (%)	CP10 (%)	CP15 (%)	R
**All**	-	0.05±4.61	3.402	-	-	-	-	-
**Hypotensive**	-	-0.11±2.90	-	-	-	-	-	-
**Normal**	-	0.03±3.18	-	-	-	-	-	-
**Hypertension**	-	-0.07±6.74	-	-	-	-	-	-

DBP/ Group*	ΔBP±SD (mmHg)	MD±SD (mmHg)	MAD (mmHg)	MAPD (%)	CP5 (%)	CP10 (%)	CP15 (%)	R
**All**	-	-0.01±2.12	1.615	-	-	-	-	-
**Hypotensive**	-	-0.01±1.38	-	-	-	-	-	-
**Normal**	-	-0.01±1.75	-	-	-	-	-	-
**Hypertension**	-	0.03±2.93	-	-	-	-	-	-

*BP profiles not classified based on ACC/AHA

Visualization formats:

(c) Bland-Altman plot **(b) Scatter plot**

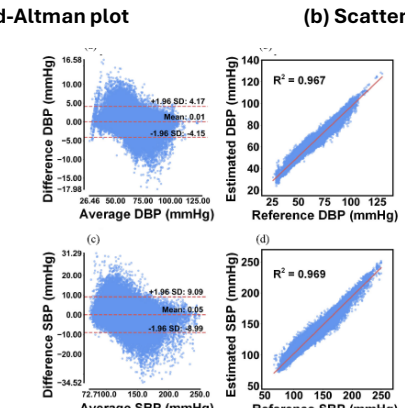

Supplementary Figure 14: Calibration-free model card corresponding to the study by Ma et al. (2024): SMART-BP: SEM-ResNet and Auto-Regressor Based on a Two-Stage Framewrok for Noninvasive Blood Pressure Measurement.

(Cisnal et al., 2024): Robust Feature Selection for BP Estimation in Multiple Populations

MODULE 0: CLINICAL PURPOSE AND USE CONTEXT

Model name / version: Robust-Feature Selection XGBoost model
Estimation family: [ ] Hemodynamics-derived [x] Data-driven BP regression [ ] ABP waveform reconstruction
Calibration paradigm: [ ] Calibration-free [x] Calibration-based
Model type: [x] Machine learning (ML) [ ] Deep learning (DL) [ ] Hybrid [ ] Other
Input data type: [x] Handcrafted features [ ] Time series [ ] Images [x] Demographic [ ] Other
Output variable: [x] SBP [x] DBP [ ] MAP
Signal modality: [x] PPG [ ] ECG [ ] BCG [ ] BioZ [ ] IPG [ ] iPPG [ ] Other
Sensor specification: PPG optical MAX30101
Sensor location: PPG wrist (opposite to the cuff-based BP)
Intended use: [x] Research use only [x] Home / ambulatory monitoring [ ] Adjunctive clinical monitoring [ ] Clinical pilot / prospective evaluation

MODULE 1: DATASET AND COHORT REPORTING

Dataset source: [ ] Private [x] Public: AURORA-BP
Participants (number of subjects): 534
Recording sessions: 24-hour recording + in-clinic sessions
Total paired BP samples: 55.70 ± 4.11 per subject
Population type: [x] Healthy [x] Hypertensive [ ] ICU [x] Other: General population, ambulatory cohort
Age (years): NA
Sex: NA
BMI (kg/m²): NA
Height (cm): NA
Weight (kg): NA
Ethnicity / geographic region: NA
Medical history / comorbidities reported: [ ] Yes [x] No
Medication reported: [ ] Yes [x] No
Known sensitivity: [x] Motion [ ] Low perfusion [ ] Arrhythmia [x] Sensor displacement [ ] Skin tone [x] Other: ambulatory noise (light sensitivity)
Skin tone / Fitzpatrick: NA (if PPG-based)
Ambient light conditions: NA (if PPG-based)

Measurement phases (n, total paired BP samples per subject):
Ambulatory 41.55 ± 4.07 | In-clinic 14.15 ± 0.68 | Dynamic: 0| Long-term: 0 |Sleep: 0
BP profiles (%):
Hypotension: 0| Normal: 16.8| Elevated: 23.9| Stage 1: 35.9| Stage 2: 23.3
SBP (mmHg): NA
DBP (mmHg): NA
Baseline SBP (mmHg): 131.15 ± 15.53
Baseline DBP (mmHg): 86.21 ± 10.88
ΔSBP (mmHg): 54.03 ± 16.25 [x] Subject-specific [ ] Population-based
ΔDBP (mmHg): 40.72 ± 11.87 [x] Subject-specific [ ] Population-based

Preprocessing pipeline: 4-th order Butterworth bandpass filter, baseline removal (airPLS), pulse segmentation with adaptive thresholds
Signal quality filtering / exclusion criteria: morphology-based and template matching, 30-s segments discarded if < 5 valid pulses
Fiducial points / feature extraction: 9 fiducial points, > 500 features
Excluded samples after cleaning: Post-processing ratio of available measurements: 56.97 ± 7.22%; signal retention rate: 35.22 ± 35.00%
BP distribution before/after cleaning reported: [ ] Yes [ ] No

BP distribution :

(a) BP distribution histogram

Not reported

(b) calibration-relative BP distribution

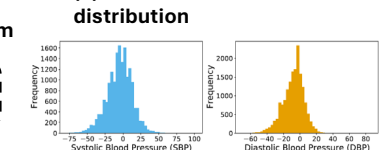

MODULE 2. VALIDATION STRATEGY

Evaluation method: [ ] Subject-wise hold-out [ ] Subject-wise CV [x] LOSO / LOOCV [ ] Record-wise split [ ] Sample-wise split
Data split details: outer loop: LOOCV; inner loop: 10-fold CV hyperparameter tunning
Unseen-subject test set: [x] Yes [ ] No
External validation (independent cohort / site): [ ] Yes [x] No
Reference standard: Oscillometric cuff-based BP monitor
Reference device (manufacturer/model): Spacelabs Healthcare OnTrak 90227 ABP

Measurement conditions: [x] Resting [x] Ambulatory [x] Dynamic [ ] Long-term [ ] ICU monitoring [ ] Other
Body position: Sitting arm down/lap/up
Activity state: Walking/running/cooling-down activities included
Rest period prior to measurement: NA
Baseline comparator: NA
Comparator result: XGBoost model

Minimum acceptable standard met: No; only in-clinic DBP met IEEE criteria [x] Subject-wise validation

MODULE 3. PERFORMANCE REPORTING

Models evaluated: [] Baseline model [x] Proposed / final model

Calibration condition: Resting
Calibration type: [x] One-point [ ] Two-point [ ] Multi-point [ ] Other
Static test: [x] Yes [ ] No **Dynamic test:** x] Yes [ ] No
Time since calibration evaluated: [ ] Yes [x] No;
Recalibration trigger (if any): NA

Standarized performance reporting table:

SBP/ Condition	ΔBP±SD (mmHg)	MD±SD (mmHg)	MAD (mmHg)	MAPD (%)	CP5 (%)	CP10 (%)	CP15 (%)	R
In-clinic	-	0.63 ± 11.73	8.49	6.34	-	-	-	-
Dynamic (Ambulatory)	-	-0.33 ± 14.54	11.35	9.54	-	-	-	-
Time after calibration	-	-	-	-	-	-	-	-

DBP/ Condition	ΔBP±SD (mmHg)	MD±SD (mmHg)	MAD (mmHg)	MAP D (%)	CP5 (%)	CP10 (%)	CP15 (%)	R
In-clinic	-	0.01 ± 6.58	5.03	5.03	-	-	-	-
Dynamic (ambulatory)	-	-0.22 ± 10.10	7.85	11.15	-	-	-	-
Time after calibration	-	-	-	-	-	-	-	-

Visualization formats:

(e) Scatter plot

Not reported

(f) Bland-Altman plot

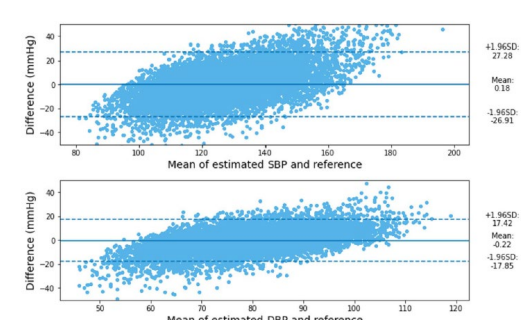

Supplementary Figure 15: Calibration-based model card corresponding to the study by Cisnal et al. (2024): Robust Feature Selection for BP Estimation in Multiple Populations: Towards Cuffless Ambulatory BP Monitoring.

(Cisnal et al., 2025): Are PAT-based Models Suitable for Continuous Blood Pressure Monitoring in Ambulatory Settings?

MODULE 0: CLINICAL PURPOSE AND USE CONTEXT

Model name / version: PAT-based models
Estimation family: [x] Hemodynamics-derived [ ] Data-driven BP regression [ ] ABP waveform reconstruction
Calibration paradigm: [x] Calibration-free [ ] Calibration-based
Model type: [x] Machine learning (ML) [ ] Deep learning (DL) [ ] Hybrid [ ] Other
Input data type: [x] Handcrafted features [ ] Time series [ ] Images [ ] Demographic [ ] Other
Output variable: [x] SBP [x] DBP [ ] MAP
Signal modality: [x] PPG [x] ECG [ ] BCG [ ] BioZ [ ] IPG [ ] iPPG [ ] Other
Sensor specification: NA
Sensor location: NA
Intended use: [x] Research use only [ ] Home / ambulatory monitoring [ ] Adjunctive clinical monitoring [ ] Clinical pilot / prospective evaluation

MODULE 1: DATASET AND COHORT REPORTING

Dataset source: [ ] Private [x] Public: AuroraBP
Participants (number of subjects): 537 (after processing 102)
Recording sessions: 3 (initial and final in-clinic visit and 24-hour ambulatory recording) **Total paired BP samples:** 52 per subject

Population type: [x] Healthy [ ] Hypertensive [ ] ICU [ ] Other: general population, ambulatory cohort
Age (years): NA
Sex: NA
BMI (kg/m²): NA
Height (cm): NA
Weight (kg): NA
Ethnicity / geographic region: NA
Medical history / comorbidities reported: [ ] Yes [x] No
Medication reported: [ ] Yes [x] No
Known sensitivity: [ ] Motion [ ] Low perfusion [ ] Arrhythmia [ ] Sensor displacement [ ] Skin tone [ ] Other
Skin tone / Fitzpatrick: NA (if PPG-based)
Ambient light conditions: NA (if PPG-based)

Measurement phases (n, per subject):
Ambulatory: 36-48 | Static: 11 | Dynamic: 4 | Long-term: 0 |Sleep: 0
BP profiles (%):
Hypotension: 0| Normal: 0| Elevate : 0 | Stage 1: 0 | Stage 2: 0
SBP (mmHg): 131.27 ± 15.44 (initial); 128.84 ± 16.31 (after processing)
DBP (mmHg): 86.31 ± 10.87 (initial); 85.96 ± 10.84 (after processing)
Baseline SBP (mmHg): NA
Baseline DBP (mmHg): NA
ΔSBP (mmHg): 48.00±12.12 (final) [x] Subject-specific [ ] Population-based
ΔDBP (mmHg): 34.98±10.06 (final) [x] Subject-specific [ ] Population-based

Preprocessing pipeline: bandpass filter; baseline correction; beat-to-beat waveform extraction; pulse normalization
Signal quality filtering / exclusion criteria: pulse morphology and template matching
Fiducial points / feature extraction: derivative-based analysis (5 fiducial points PPG and R-peak in ECG)
Excluded samples after cleaning: 435 participants
BP distribution before/after cleaning reported: [x] Yes [ ] No

(a) BP distribution histogram

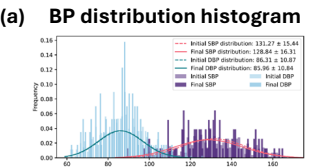

(b) Calibration-relative BP distribution

Not provided

MODULE 2. VALIDATION STRATEGY

Evaluation method: [x] Subject-wise hold-out [ ] Subject-wise CV [ ] LOSO / LOOCV [ ] Record-wise split [ ] Sample-wise split
Data split details: at least 5 pair values for training (static and dynamic in-clinic conditions) and at least 10 pair values for testing (ambulatory and 24-hour later static in-clinic conditions)
Unseen-subject test set: [ ] Yes [x] No
External validation (independent cohort / site): [ ] Yes [x] No
Reference standard: clinically validated ambulatory blood pressure (ABP) monitor
Reference device (manufacturer/model): NA

Measurement conditions: [x] Resting [x] Ambulatory [x] Dynamic [ ] Long-term [ ] ICU monitoring [x] Other: after 24-hour
Body position: seated, supine, standing, ambulatory
Activity state: resting, post-exercise, ambulatory
Rest period prior to measurement: NA
Baseline comparator: BP when sitting and arm down (in-clinic measurement)
Comparator result: PAT-based model

Minimum acceptable standard met: No [x] Subject-wise validation

MODULE 3. PERFORMANCE REPORTING *(Calibration-based models)*

Models evaluated: [x] Baseline model [x] Proposed / final model

Calibration condition: at least 5 pair values for training (static and dynamic In-clinic conditions) and at least 10 pair values for testing (ambulatory and static And dynamic in-clinic conditions)
Calibration type: [ ] One-point [ ] Two-point [x] Multi-point [ ] Other
Static test: [ ] Yes [x] No **Dynamic test:** [ ] Yes [x] No
Time since calibration evaluated: [ ] Yes [x] No
Recalibration trigger (if any): NA

Performance reporting table (SBP estimation: M5-PATs; DBP estimation: M2-PATo)

SBP/ Condition	ΔBP±SD (mmHg)	MD±SD (mmHg)	MAD (mmHg)	MAPD (%)	CP5 (%)	CP10 (%)	CP15 (%)	R
Static	-	-	-	-	-	-	-	-
Dynamic	48.00±12.12	10.15±14.60	14.23	12	6.79	20.54	38.72	
Time after calibration	-	-	-	-	-	-	-	-

DBP/ Condition	ΔBP±SD (mmHg)	MD±SD (mmHg)	MAD (mmHg)	MAPD (%)	CP5 (%)	CP10 (%)	CP15 (%)	R
Static	-	-	-	-	-	-	-	-
Dynamic	34.98±10.06	-5.33±9.79	9.67	14	11.29	32.94	53.75	
Time after calibration	-	-	-	-	-	-	-	-

Performance reporting table (baseline model**)**

SBP/ Condition	ΔBP±SD (mmHg)	MD±SD (mmHg)	MAD (mmHg)	MAPD (%)	CP5 (%)	CP10 (%)	CP15 (%)	R
Static	-	-	-	-	-	-	-	-
Dynamic	48.00±12.12	-5.34±12.58	13.04	-	-	-	-	-
Time after calibration	-	-	-	-	-	-	-	-

DBP/ Condition	ΔBP±SD (mmHg)	MD±SD (mmHg)	MAD (mmHg)	MAPD (%)	CP5 (%)	CP10 (%)	CP15 (%)	R
Static	-	-	-	-	-	-	-	-
Dynamic	34.98±10.06	-7.83±9.48	10.91	-	-	-	-	-
Time after calibration	-	-	-	-	-	-	-	-

Visualization formats:

(e) Scatter plot (SBP proposed model)

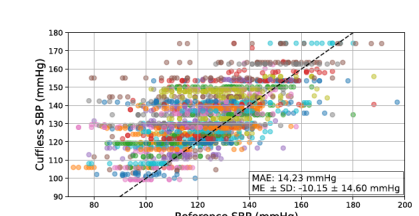

(f) Bland-Altman plot

Not provided

(e) Scatter plot (SBP baseline model)

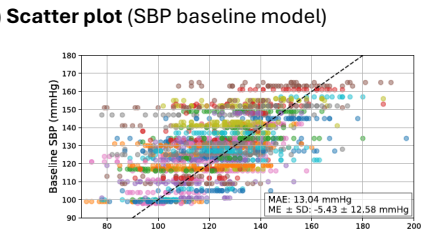

(f) Bland-Altman plot

Not provided

Supplementary Figure 16: Calibration-based model card corresponding to the study by Cisnal et al. (2025): Are PAT-based Models Suitable for Continuous Blood Pressure Monitoring in Ambulatory Settings?.
